# Pathology Tissue-quantitative Mass Spectrometry Analysis to Profile Histone Post-translational Modification Patterns in Patient Samples* [Fn FN2]

**DOI:** 10.1074/mcp.M115.054510

**Published:** 2015-10-13

**Authors:** Roberta Noberini, Andrea Uggetti, Giancarlo Pruneri, Saverio Minucci, Tiziana Bonaldi

**Affiliations:** From the ‡Center for Genomic Science of IIT@SEMM, Istituto Italiano di Tecnologia, Via Adamello 16, 20139 Milan, Italy;; §Biobank for Translational Medicine Unit, Department of Pathology, European Institute of Oncology, Via Ripamonti 435, 20141 Milano;; ¶School of Medicine, University of Milan, 20122 Milan, Italy;; ‖Department of Experimental Oncology, European Institute of Oncology, Via Adamello 16, 20139 Milan, Italy;; **Drug Development Program, European Institute of Oncology, Via Adamello 16, 20139 Milan, Italy;; ‡‡Department of Bioscience, University of Milan, 20133 Milan, Italy

## Abstract

Histone post-translational modifications (hPTMs) generate a complex combinatorial code that has been implicated with various pathologies, including cancer. Dissecting such a code in physiological and diseased states may be exploited for epigenetic biomarker discovery, but hPTM analysis in clinical samples has been hindered by technical limitations. Here, we developed a method (PAThology tissue analysis of Histones by Mass Spectrometry - PAT-H-MS) that allows to perform a comprehensive, unbiased and quantitative MS-analysis of hPTM patterns on formalin-fixed paraffin-embedded (FFPE) samples. In pairwise comparisons, histone extracted from formalin-fixed paraffin-embedded tissues showed patterns similar to fresh frozen samples for 24 differentially modified peptides from histone H3. In addition, when coupled with a histone-focused version of the super-SILAC approach, this method allows the accurate quantification of modification changes among breast cancer patient samples. As an initial application of the PAThology tissue analysis of Histones by Mass Spectrometry method, we analyzed breast cancer samples, revealing significant changes in histone H3 methylation patterns among Luminal A-like and Triple Negative disease subtypes. These results pave the way for retrospective epigenetic studies that combine the power of MS-based hPTM analysis with the extensive clinical information associated with formalin-fixed paraffin-embedded archives.

Histone post-translational modifications (hPTMs)^1^ generate a complex combinatorial code that plays a critical role during the physiological and pathological regulation of gene expression (1). Alterations in histone modification patterns have been linked with various diseases, including cancer, often as a result of the aberrant expression or localization of histone modifying enzymes (2). Therefore, accurately dissecting hPTM patterns in normal and diseased tissues could yield epigenetic biomarkers useful for prognostic, diagnostic, and therapeutic purposes. Immunohistochemistry studies have shown the potential of this strategy (3, 4), but they were limited to the analysis of only a few hPTMs. In addition, despite their sensitivity and ease of use, antibody-based assays are hindered by issues such as the difficulty in detecting adjacent modifications and the limited linearity of the signal. As an alternative to traditional antibody-based methods, in recent years MS has become the elective method to analyze hPTMs, thanks to its unbiased nature, accuracy and its ability to quantitate modifications and detect their combinations. Various MS-based workflows optimized for hPTM analysis have been developed (5), but most of the studies focused on cell lines and animal tissue, whereas the potential offered by the analysis of clinical samples has been left largely unexploited. In particular, the MS-based analysis of hPTMs from formalin-fixed paraffin-embedded (FFPE) samples has never been addressed.

Paraffin embedding following fixation in buffered formalin is the storage method of choice for clinical specimens, thus representing an invaluable source of clinical samples linked to retrospective patient information. Large formalin-fixed paraffin embedded (FFPE) archives, which are available in many hospitals, have been successfully exploited for DNA and RNA analyses, including chromatin immunoprecipitation (6, 7). However, the extensive protein cross-linking generated by formaldehyde fixation has hindered the proteomic study of this type of tissue. This problem has been addressed and overcome only recently in global proteomic studies by taking advantage of extraction protocols based on heat-induced antigen retrieval techniques derived from immunohistochemistry (8, 9). Moreover, a few studies showed the possibility to globally analyze protein post-translational modifications, such as glycosylation and phosphorylation, from fixed and embedded tissues (10–12).

Here, we report for the first time the successful application of MS-based analysis of hPTMs to human clinical samples, focusing in particular on the development and validation of a method (PAT-H-MS) to extract histones from FFPE tissues in yield and purity sufficient to enable the subsequent use of a proteomic workflow optimized for hPTM analysis (13). By using this method we were able to profile in a quantitative manner 24 distinct modified histone peptides from human FFPE breast cancer samples belonging to different subtypes, identifying differences in histone methylation patterns of potential clinical relevance. Thus, PAT-H-MS represents a valid approach for hPTM analysis of clinical samples.

## EXPERIMENTAL PROCEDURES

### 

#### 

##### Preparation of Frozen and FFPE Tissues

Leukemic blasts were isolated from acute promyelocytic leukemia transgenic mice and 1 × 10^6^ cells were i.v. injected in a syngenic recipient mouse to induce secondary leukemia (14). After massive splenomegaly was established, the mouse was sacrificed and its spleen was divided into two portions. One portion was washed and homogenized in 5 ml ice-cold phosphate buffered saline (PBS: 0.8% NaCl; 0.02% KCl; 0.02% KH_2_PO_4_; 0.2% Na_2_HPO_4_, pH 7.4) using a Dounce homogenizer, obtaining spleen cells that were counted, pelleted by centrifugation, rapidly frozen, and stored at −80 °C until use. The other half of the spleen was washed in PBS and incubated for 16 h at room temperature in a 4% paraformaldehyde solution. The fixed spleen was then routinely dehydrated with increasing concentrations of ethanol (70%, 80%, 90 and 100%) and subsequently included in paraffin using a tissue processor (Leica ASP300). Frozen cells and FFPE tissues were prepared similarly from mouse liver. Experimental procedures involving animals complied with the Guidelines of the Italian National Institute of Health, and were approved by the Institutional Ethical Committee.

Invasive breast cancer specimens were obtained from 23 patients with duct invasive carcinoma not otherwise specified (supplemental Fig. S3*A* and supplemental Table S3), who were subjected to mastectomy or breast conserving surgery. The patients provided informed consent and this study was approved by the Ethical Committee of the European Institute of Oncology. Tumor samples were collected and snap frozen or fixed 4% formalin and embedded in paraffin. Tumor cells accounted for 50% or more of the samples, as evaluated histologically. The assessment of hormone receptors and Her-2 status, as well as the *K_i_*-67 labeling index was ascertained by immunohistochemistry, according to the ASCO/CAP recommendations, using the FDA-approved anti-ER/PgR (PharmDX, Dako) and Her-2 (Herceptest, Dako), as well as the anti-*K_i_*-67 antibody MIB-1. Breast cancer subtypes were defined according to the 2013 S. Gallen consensus conference recommendations, using immunohistochemical surrogates as follows: Luminal A-like: ER and/or PgR(+), HER2(-), Ki67 < 20%; Luminal B-like: ER and/or PgR(+), HER2(-), Ki67 ≥ 20; Triple Negative: ER, PgR and HER2(-), irrespective of Ki67 score; HER2-positive: HER2(+), irrespective of ER, PgR or Ki67. ER/PgR positivity was defined as ≥1% of immunoreactive neoplastic cells; HER2 positivity was defined as >10% of neoplastic cells with strong and continuous staining of the cell membrane (3+ by immunohistochemistry) and/or amplified by *in situ* hybridization techniques, in accordance to the ASCO/CAP guidelines.

##### Histone Isolation from FFPE Tissue (Fig. S1A)

Four 10-μm tissue sections (corresponding to ∼20–60 mg of tissue, as measured by weighting the tissue after paraffin removal) were deparaffinized by adding 1 ml of hystolemon (Dasit Group Carlo Erba), vortexing for 30 s and centrifuging at 18,000 × *g* for 3 min (5 times). The samples were rehydrated with incubations in decreasing concentrations of ethanol (100%, 95%, 70%, 50%, 20%, and water) for 3 min at room temperature, followed by centrifugation for 3–5 min at 18,000 × *g* for each step. Rehydrated FFPE sections were permeabilized in 0.5 ml of Tris-buffered saline (TBS) containing 0.5% Tween20 and protease inhibitors for 20 min at room temperature in a rotating platform, followed by a 5 min centrifugation at 18,000 × *g*. The samples were then resuspended in 200 μl of 20 mm Tris pH 7.4 containing 2% SDS and sonicated in a Branson Digital Sonifier 250 with a 3 mm microtip until tissues pieces were homogenized. Proteins were extracted and de-crosslinked with a 45 min incubation at 95 °C, followed by a 4 h incubation at 65 °C. Protein concentration was estimated with the Bio-Rad DC protein assay kit (Bio-Rad, Segrate, Italy) and 16–20 μg of proteins were run on a 17% SDS-PAGE gel following protein detection with colloidal Coomassie staining (Expedeon, San Diego, CA) to quantify histone concentration based on a comparison with known amounts of recombinant histone H3.1 (New England Biolabs, Ipswich, MA). Acidic extraction, a step typically performed to purify histones, was omitted because it cannot be performed in the presence of detergents. Nevertheless, histones represented a good portion of the FFPE protein extract (supplemental Fig. S3*B*). The reproducibility of the histone isolation protocol was assessed by analyzing adjacent FFPE sections from the same mouse spleen (supplemental Fig. S2).

##### Histone Isolation from Fresh-frozen Mouse Spleen and Liver Tissue and Breast Cancer Cell Lines (supplemental Fig. S1C-D)

Cells were obtained from fresh mouse spleen and liver as described above and histones were isolated as previously described (15) (supplemental Fig. S1*C*). Briefly, 30 × 10^6^ cells were resuspended in lysis buffer (10% sucrose; 0.5 mm EGTA, pH 8.0; 15 mm NaCl; 60 mm KCl; 15 mm HEPES; 0.5% Triton; 0.5 mm PMSF; 1 mm DTT; 5 mm NaF; 5 mm Na_3_VO_4_; 5 mm Na-butyrate; protease inhibitors) and nuclei were separated from the cytoplasm by centrifugation on a sucrose cushion. Histones were extracted by a 4 hours incubation in 0.4 N HCl at 4 °C and dialyzed overnight against 100 mm CH_3_COOH, using dialysis membranes with a 6–8 kDa cutoff (Spectrum Laboratories, Inc, Rancho Dominguez, CA). The dialyzed samples were lyophilized and stored at −80 °C. The same procedure was used to isolate histones from the breast cancer cell lines used to generate the super-SILAC mix (supplemental Fig.S1D, see below).

##### Histone Isolation from Fresh-frozen Human Breast Cancer Tissue (supplemental Fig. S1B)

Because breast tissue cannot be readily homogenized to single cells as described above for spleen and liver tissues and is available in limited amounts, we developed and alternative protocol to obtain histones. Twenty to seventy mg of frozen breast cancer tissue were thawed on ice, cut in pieces as small as possible with scissors and then homogenized in PBS containing 0.1% Triton X-100 and protease inhibitors using a Dounce homogenizer. Tissue debris were removed by filtering the homogenate through a 100 μm cell strainer and nuclei were isolated with a 10 min centrifugation at 2300 × *g*. Nuclei were resuspended in 100–200 μl of the same buffer containing 0.1% SDS and incubated for few minutes at 37 °C in the presence of 250 U of benzonase (Merk Millipore, Darmstadt, Germany) to digest nucleic acids. No acidic extraction was performed to avoid samples loss. The concentration of purified histones and nuclei extracts was measured using the Bradford protein assay kit (Thermo Fisher Scientific) and their purity was assessed by SDS-PAGE. Yield ranged between 0.5 and 1.3 μg of histone octamer per 10 mg of starting tissue.

##### Histone Digestion

About 5 μg of histones per run per sample were separated on a 17% SDS-PAGE gel and bands corresponding to histones H3 and H4 were excised and in-gel digested as previously described (15). Briefly, gel bands were cut in pieces and destained with repeated washes in 50% acetonitrile (ACN) in H_2_O, alternated with dehydration steps in 100% ACN. Gel pieces were then in-gel chemically alkylated with D_6_-acetic anhydride (Sigma-Aldrich) 1:9 in 1 m NH_4_HCO_3_, using CH_3_COONa as catalyzer. After shaking for 3 h at 37 °C, chemically modified gel slices were washed with NH_4_HCO_3_, alternated with ACN at increasing percentages (from 50 to 100). The in-gel digestion was performed overnight with 100 ng/μl trypsin (Promega) in 50 mm NH_4_HCO_3_ at 37 °C, in order to obtain an “Arg-C like” in-gel digestion that originates histone peptides of optimal length for MS analysis by cleaving at the C-terminal of arginine residues. Finally, digested peptides were extracted using 5% formic acid alternated with ACN 100%. In SILAC experimental set-ups, unlabeled and heavy-labeled histones were mixed in equal amounts prior to gel separation, and then processed as described above. Digested peptides were desalted and concentrated using a combination of reversed-phase C_18_/C and strong cation exchange (SCX) chromatography on handmade nanocolumns (StageTips). Digested peptides were then eluted with 80% ACN/0.5% acetic acid and 5% NH_4_OH/30% methanol from C_18_/C and SCX StageTips, respectively. Eluted peptides were lyophilized, resuspended in 1% TFA, pooled and subjected to LC-MS/MS analysis.

##### LC-MS/MS

Peptide mixtures were separated by reversed-phase chromatography on an in-house-made 25 cm column (inner diameter 75 μm, outer diameter 350 μm outer diameter, 1.9 μm ReproSil, Pur C18AQ medium), using a ultra nanoflow high-performance liquid chromatography (HPLC) system (EASY-nLC™ 1000, Thermo Fisher Scientic) connected online to a Q Exactive instrument (Thermo Fisher Scientific) through a nanoelectrospray ion source. Solvent A was 0.1% formic acid (FA) in ddH_2_O and solvent B was 80% ACN plus 0.1% FA. Peptides were injected in an aqueous 1% TFA solution at a flow rate of 500 nl/min and were separated with a 100 min linear gradient of 0–40% solvent B, followed by a 5 min gradient of 40–60% and a 5 min gradient of 60–95% at a flow rate of 250 nl/min. The Q Exactive instrument was operated in the data-dependent acquisition (DDA) mode to automatically switch between full scan MS and MS/MS acquisition. Survey full scan MS spectra (*m*/*z* 300–1650) were analyzed in the Orbitrap detector with resolution of 35,000 at *m*/*z* 400. The five most intense peptide ions with charge states ≥ 2 were sequentially isolated to a target value for MS1 of 3 × 10^6^ and fragmented by HCD with a normalized collision energy setting of 25%. The maximum allowed ion accumulation times were 20 msec for full scans and 50 msec for MS/MS and the target value for MS/MS was set to 1 × 10^6^. The dynamic exclusion time was set to 20 s and the standard mass spectrometric conditions for all experiments were as follows: spray voltage of 2.4 kV, no sheath and auxiliary gas flow.

One technical replicate of the breast cancer cell lines profiled to set up the super-SILAC strategy (see below) was analyzed through HPLC in combination with a LTQ-Velos Orbitrap. Samples were separated by nano-liquid chromatography using an EASY-nLC system (Proxeon Biosystems, Odense, Denmark) on an in-house-made 50 cm column (inner diameter 75 μm, outer diameter 350 μm outer diameter, 3 μm ReproSil, Pur C18AQ medium) and analyzed using an LTQ-Velos Orbitrap instrument (Thermo Fisher Scientific). The solvent composition were as described above, and peptides were separated with a shallow gradient at a flow rate of 300 nl/min: 10–50% solvent B over 95 min, 50–60% over 5 min and 60–80% over 5 min. The LTQ-Velos Orbitrap was operated in DDA mode and MS spectra (m/z range 300–1650) were analyzed with resolution *r* = 30,000 at *m*/*z* 400 (transient time = 250 ms). The 10 most intense peptide ions with charge states ≥ 2 were sequentially isolated to a target value of 1 × 10^6^ and fragmented by CID with a normalized collision energy setting of 35%. MS/MS settings were: maximum ion injection time 150 ms, minimum signal threshold 500; isolation width 2 Da; ion target value 1e4, dynamic exclusion time 25 s. Results obtained using the two LC/MS setups generated very similar results (supplemental Fig. S4*C*).

##### Data Analysis

Acquired RAW data were analyzed by the integrated MaxQuant software v.1.3.0.5, which performed peak list generation and protein identification using the Andromeda search engine (16). The Uniprot MOUSE 1301 (33202 entries) and HUMAN 1301 (43990 entries) databases were used for peptide identification. Enzyme specificity was set to Arg-C. The estimated false discovery rate of all peptide identifications was set at a maximum of 1%. The mass tolerance was set to 6 ppm for precursor and fragment ions. Three missed cleavages were allowed, and the minimum peptide length was set to 6 amino acids. We focused on lysine methylation and acetylation, including as variable modifications lysine D_3_-acetylation (+45.0294 Da), lysine monomethylation (+ 59.0454, corresponding to the sum of D_3_-acetylation (+45.0294) and monomethylation (+14.016 Da)), dimethylation (+28.031 Da), trimethylation (+42.046 Da), and lysine acetylation (+42.010 Da). To reduce the search time and the rate of false positives, which increase with increasing the number of variable modifications included in the database search (17), the raw data were analyzed through multiple parallel MaxQuant jobs (18), setting different combinations of variable modifications: (1) D_3_-acetylation, lysine monomethylation with D_3_-acetylation, dimethylation and lysine acetylation, (2) D_3_-acetylation, lysine monomethylation with D_3_-acetylation, dimethylation and trimethylation, (3) D_3_-acetylation, lysine monomethylation with D_3_-acetylation, trimethylation, and lysine acetylation. In addition, for the mouse samples we included in the search lysine modifications that may be induced by formalin fixation (19): formylation (+27.9949 Da), methylene adducts (+12 Da) and methylol adducts (+30.0106 Da), which were included in a fourth search job together with D3-acetylation. MaxQuant search results from different jobs were exported and combined; peptides with Andromeda score less than 60 (corresponding to a Mascot score of 15 (16), which has been previously used as a cut-off value (20)) and localization probability score less than 0.75 (21, 22), were removed. Modified peptide parameters and representative MS/MS spectra for each modified peptide are reported in supplemental Table S4 and supplemental Fig. S8, respectively. Identifications and retention times were used to guide the manual quantification of each modified peptide using QualBrowser version 2.0.7 (ThermoFisher Scientific, Waltham, MA). Site assignment was evaluated using QualBrowser and MaxQuant Viewer 1.3.0.5. The mass spectrometry proteomics data have been deposited to the ProteomeXchange Consortium (23) via the PRIDE partner repository with the data set identifier PXD002669. Extracted ion chromatograms (XIC) were constructed for each doubly charged precursor based on its *m/z* value, using a mass tolerance of 10 ppm and a mass precision up to four decimals. For each histone modified peptide, the relative abundance (RA) was estimated by dividing the area under the curve (AUC) of each modified peptide for the sum of the areas corresponding to all the observed forms of that peptide (24). For SILAC experiments, Arg10 was selected as heavy label (multiplicity = 2) in MaxQuant. The heavy form of each modified peptide was quantified from its XIC and the relative abundance quantified. L/H ratios of relative abundances were calculated for each modified peptide (supplemental Fig. S5*A*). Because we assume that the sum of all the possible forms of the peptide should be constant in all the samples, using ratios of relative abundances corrects for possible errors in mixing light and heavy histones. To better visualize differences among biopsies the ratio of one sample relative to the standard was divided by the average ratios across the samples (supplemental Fig. S5*C*). As an alternative normalization strategy, SILAC ratios were obtained from light and heavy AUC values and were corrected based on the SILAC ratio of an unmodified histone H3 peptides (supplemental Fig. S5*B* and S5*D*). The results obtained using the two normalization strategies were very similar, suggesting that other possible modifications not specifically indicated in the database search do not substantially affect the quantification based on the estimation of %RA. Visualization of hPTM ratios and unsupervised hierarchical clustering were performed using Perseus, setting correlation distance and complete linkage as parameters (http://www.perseus-framework.org/).

##### Experimental Design and Statistical Rationale

FFPE and frozen samples from mouse tissues were acquired in duplicate (technical replicates for all samples with the exception of spleen samples, for which two biological replicates were analyzed, see supplemental Data set S1). FFPE and frozen samples from human breast cancer biopsies 1–3 were acquired in triplicate (technical replicates, see supplemental Data set S2). The analysis of five patient biopsies for each subtype allowed assessing differences among breast cancer subtypes (supplemental Data set S3). Differences among L/H ratios for breast cancer subtypes were analyzed by one-way ANOVA followed by Bonferroni's Post-hoc test using GraphPad Prism. Differences between Luminal A-like and Triple Negative samples were assessed by T-Test analysis.

##### Set Up of the Super-SILAC Mix Approach

Breast cancer and MCF10A cell lines were obtained from ATCC. MCF10A cells were grown DMEM/Ham's F12 (1:1) (Lonza, Basel, Switzerland), supplemented with 5% dialyzed fetal bovine serum (FBS) (Thermo Fisher Scientific), 2 mm l-glutamine, 20 ng/ml human epidermal growth factor, 50 ng/ml cholera toxin, 10 ng/ml human insulin and 0.5 g/ml human transferrin. Breast cancer cells were grown in SILAC-DMEM (Euroclone, Pero, Italy) supplemented with 2 mm l-glutamine, 146 mg/l of lysine (Sigma-Aldrich), 84 mg/l l-^13^C_6_^15^N_4_-arginine (Arg-10, Sigma-Aldrich), 10% dialyzed serum (Thermo Fisher Scientific) and penicillin/streptomycin. Breast cancer cells were cultured for ∼8 doublings in SILAC medium to obtain complete labeling. To identify a pool of cell lines that would appropriately represent the heterogeneous range of modifications possibly found in breast tumor samples, we analyzed the hPTM patterns of five breast cancer cell lines belonging to different subtypes and with different immunoprofiles (supplemental Fig. S4*A*). For this purpose we used a previously described spike-in approach (15), where unlabeled normal breast MCF10A cells are spiked into each of the five heavy-labeled breast cancer cell line samples and used as an internal standard for quantitation. (supplemental Fig. S4*B*). We evaluated histone H3 hPTM patterns of acetylations and methylations of lysine residues based on H/L ratios of relative abundances (supplemental Fig. S4*C*–S4*D*). Because they showed the most divergent patterns, we chose MDA-MB-231, MDA-MB-468, MDA-MB-453 and MDA-MB-361 to generate a super-SILAC mix that can be spiked into breast cancer samples prior to GeLC-MS analysis (supplemental Fig. S4*E*). Two set-ups were used for data acquisition (HPLC coupled with a LTQ-Velos Orbitrap or a uHPLC combined with a Q Exactive), which gave the same results (supplemental Fig. S4*C*), proving the reproducibility of the results across different instrument setups. Equal amounts of histones from MDA-MB-231, MDA-MB-468, MDA-MB-453 and MDA-MB-361 were mixed, aliquoted, lyophilized and stored at −80 °C until use.

##### Immunoblot Analysis

FFPE or frozen extracts were separated by SDS-PAGE, transferred on PVDF membranes and probed by immunoblotting with anti-H3 K27me3 (Millipore 07–449) and anti-H3 K9me3 (Abcam 8898). An anti-histone H3 antibody (Abcam 1791) was used as loading control. Images were acquired on a ChemiDoc XRS instrument (Bio-Rad) and quantified using the ImageJ software (rsb.info.nih.gov).

## RESULTS

### 

#### 

##### Set-up of the PAT-H-MS Protocol in Mouse Tissues

**Fig. 1.**
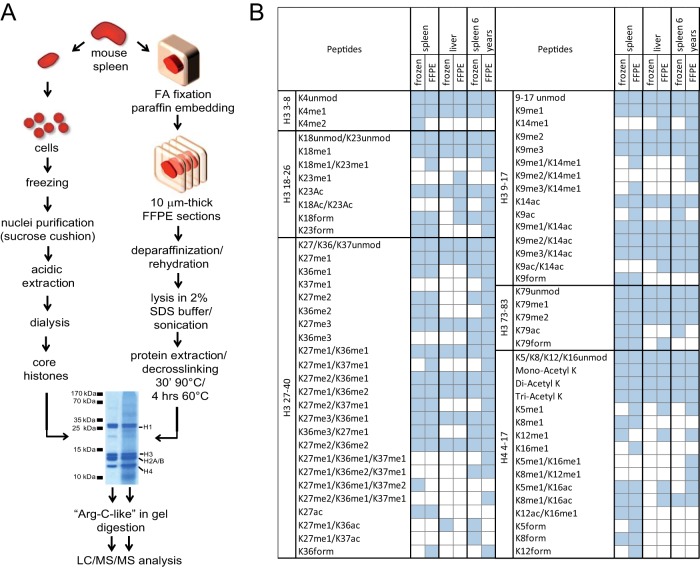
**Proteomic analysis of hPTMs from mouse FFPE tissues.** *A*, Schematic representation of the procedures used to isolate histones from mouse frozen spleen cells or FFPE mouse spleen. Five microgram of core histones purified from frozen cells and 20 μg of protein extract obtained from four 10 μm-thick FFPE sections were separated by SDS-PAGE and Comassie-stained. Gel bands corresponding to histone H3 and H4 were then excised, subjected to an in-gel Arg-C like digestion and analyzed by LC/MS/MS. A similar procedure was used for mouse frozen liver cells or FFPE mouse liver. FA: formalin. *B*, List of H3 and H4 peptides identified from frozen or FFPE samples for mouse spleen and liver stored for few weeks or mouse spleen stored for 6 years. The spleen tissues stored for few weeks or 6 years derive from different mice.

To set-up the technology to profile by mass spectrometry hPTMs from FFPE tissue we used different murine tissues, which can be obtained in large amounts and offer the possibility to compare FFPE and frozen tissue from the same samples stored for up to several years. We analyzed samples obtained from mouse spleen or liver (to prove the applicability of the protocol to different tissues) and that were stored in paraffin for short periods (few weeks) or an extended time (6 years), to evaluate the effect of storage time. To verify that the hPTMs found in the FFPE samples are representative of the real epigenetic status of the tissue, for each sample we used as control histones purified through a standard protocol from frozen cells derived exactly from the same tissue and stored for the same amount of time (Fig. 1*A*).

Proteins were extracted from FFPE samples using a protocol that relies on the presence of high percentages of detergent and the incubation at high temperatures, two factors that have been shown to be critical to solubilize proteins from FFPE tissues and to reverse the cross-links generated by formaldehyde fixation (9). In particular, high concentrations of SDS have been shown to be the most effective way to promote the solubilization of proteins from FFPE tissues and their de-crosslinking. Histones can be obtained from four 10 μm-thick paraffin sections through few straightforward steps: (1) de-paraffinization and rehydration using standard protocols, (2) lysis and homogenization by sonication in a high detergent buffer, (3) protein extraction and de-crosslinking at high temperatures and (4) SDS-PAGE, which removes contaminants and excess detergent and separates histones from other proteins present in the sample. No further purification steps are required, as histones represent a considerable portion of the sample (at least 30% based on protein concentration, Fig. 1*A*). Based on the comparison with known amounts of histones on a Coomassie-stained SDS-PAGE gel, extraction from four 10 μm-thick paraffin sections of mouse spleen typically yields 50–90 μg of histone octamer (supplemental Table S1). Histone yield varies considerably among different tissues (10–120 μg from ∼30 samples analyzed, supplemental Table S1), but for all the types of tissues tested the extraction protocol provided enough material for a full MS-based characterization of hPTMs, which usually requires 3–4 μg of histone octamer per MS run. We cut gel bands corresponding to histones H3 and H4 and in-gel digested them, using a standard procedure that involves chemical alkylation with deuterated acetic anhydride, followed by trypsin digestion (25). This procedure allows to obtain from gel bands an “Arg-C like” digestion that generates histone peptides of optimal length for the following analysis by LC/MS/MS, which was performed through reversed-phase chromatography coupled to a QExactive instrument. By analyzing the MS raw files in the software environment MaxQuant we identified all the most common lysine modifications, plus various less common ones (Fig. 1*B*). To examine possible artifacts resulting from formalin fixation, in addition to acetylation and methylation the raw files were searched for lysine formylation and other lysine modifications induced by formalin (see Experimental Procedures). Although all modifications observed in frozen samples are maintained in FFPE tissues, we observed the appearance of spurious methylations on histone H3 K14, K23 and K37, and on the histone H4 N-terminal tail, and few formylation sites on peptide H3 27–40 and peptide H4 4–17, which were absent in the frozen samples.

**Fig. 2.**
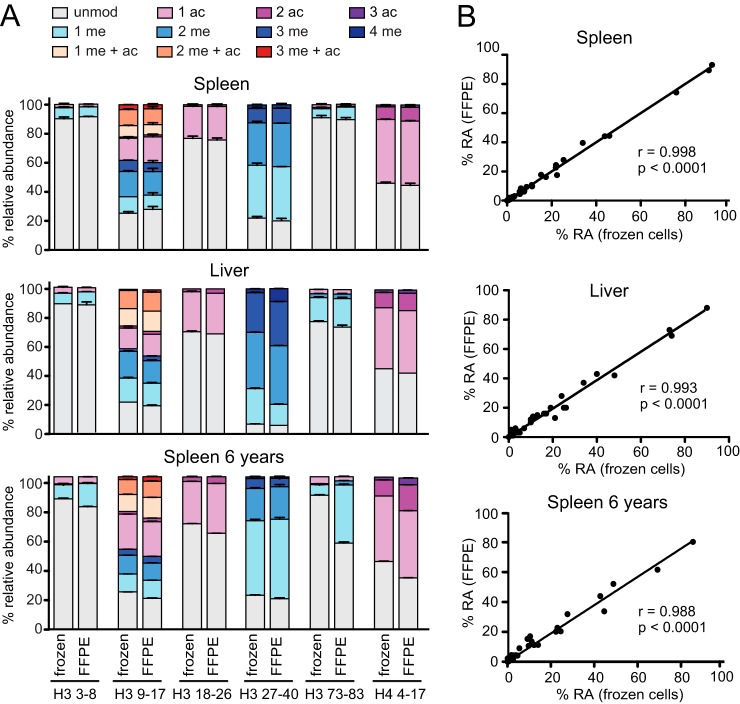
**Proteomic quantification of hPTMs from mouse FFPE tissues.** *A*, Percent relative abundances (%RA) profiles for H3 and H4 peptides from frozen or FFPE samples for mouse spleen (top panel) or liver (middle panel) stored for few weeks or mouse spleen stored for 6 years (bottom panel). Error bars represent the standard error from two independent experiments (top panel) or duplicate measurements (middle and bottom panels). *B*, hPTM %RA correlation between frozen cells and FFPE samples obtained from the mouse and liver spleen samples shown in panel *A*. Pearson correlation coefficients (r) and *p* values are shown. The spleen tissues stored for few weeks or 6 years derive from different mice.

To estimate the levels of these modifications we quantified the raw abundance of each modified peptide form using MS-extracted ion chromatograms and converted it to relative abundance percentages (%RA) by dividing the area under the curve of each modified peptide for the sum of the areas corresponding to all the observed forms identified for that peptide (24) (supplemental Data set S1, supplemental Table S2). Out of the fifteen modified peptides present only in FFPE samples, three had such a low intensity that they could not be measured, six have a relative abundance < 0.25% and four < 1%, thus representing a very minor fraction of the total. Accordingly, the comparison between FFPE and frozen tissues showed almost identical relative abundances for the most common and functionally relevant histone modifications found in four peptides from histone H3 (3–8, 9–17, 18–26 and 27–49, Fig. 2*A*). Remarkably, the %RA correlation between FFPE and frozen tissues for these peptides (*r* = 0.998, *p* < 0.0001, Fig. 2*B*) was comparable to the correlation between histone preparations obtained from adjacent FFPE sections from the same mouse spleen (*r* = 0.991, *p* < 0.0001, supplemental Fig. S2), indicating that the variability between frozen and FFPE preparations is similar to the technical variability observed in independently prepared FFPE samples. Analysis of FFPE samples after long-term storage (6 years) showed (in comparison to frozen tissues stored for the same time) an increase in K79me1 and me2 abundance and the appearance of methylated residues on histone H4 that account for ∼20% of the total (supplemental Table S2 and Fig. 2 bottom panel). These results are likely caused by the storage conditions and are in accordance with a recent analysis showing that lysine methylation is the most frequent modification induced by FFPE preservation (19). Therefore, we excluded peptides H3 73–83 and H4 4–17 from our following analysis, focusing on four histone H3 peptides carrying functionally relevant post-translational modifications (3–8, 9–17, 18–26 and 27–40), which are not affected by long-term storage.

##### Set-up of a Histone-focused Version of the Super-SILAC Approach

**Fig. 3.**
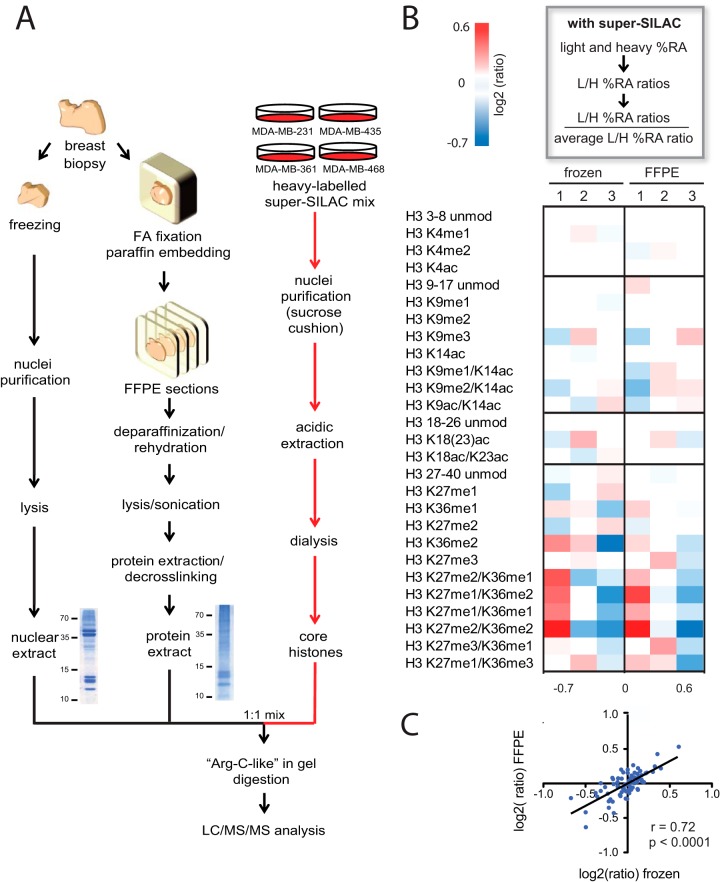
**Quantification of hPTMs from human breast cancer FFPE tissues using a super-SILAC spike-in approach.** *A*, Schematic representation of the super-SILAC spike in approach and the histone isolation procedures used to quantify histones from human fresh-frozen or FFPE breast cancer tissues, whose appearance on a Comassie-stained SDS-PAGE gel is shown. A mix of histones obtained from four heavy-labeled breast cancer cell lines is spiked into breast cancer primary samples prior to gel separation and used as an internal standard for MS-based hPTM analysis. *B*, Heatmap display of the log_2_ of ratios obtained for the indicated hPTMs for frozen and FFPE breast cancer biopsies (averages from triplicate measurements). L/H relative abundances ratios obtained with the super-SILAC strategy (light channel: breast cancer biopsy, heavy channel: spike-in super-SILAC standard) normalized over the average value across the samples are shown. *C*, Correlation of ratios obtained in frozen and FFPE samples. Pearson correlation coefficients (r) and *p* values are shown.

We next sought to apply this protocol to the hPTM analysis of clinically relevant samples (breast cancer primary tumors), combining it with a super-SILAC spike-in approach to be able to accurately quantify even small modification changes among patient samples. The stable-isotope labeling by amino acids in cell culture (SILAC) strategy relies on differential labeling of cell populations through incorporation of different isotope-encoded amino acids, which can be distinguished by MS (26). Because the differentially labeled samples can be combined early during the MS-proteomics workflow and are analyzed together by MS, the impact of experimental variation during sample preparation is reduced and the accuracy of the quantitation highly improved. Variations of this technique include the super-SILAC set up, where a mixture of SILAC-labeled cells is spiked into unlabeled samples and used as an internal reference, which allows sample multiplexing and overcomes the intrinsic unfeasibility of applying SILAC to the analysis of clinical samples (27). In addition, using a mixture of cell lines instead of a single one also offers the advantage of better representing the heterogeneity found in a complex sample, such as a tumor tissue. The super-SILAC approach has been successfully used not only for global proteomic studies, but also to analyze the phosphoproteome (28), the N-glycosilated secretome (29) and histones (30). Here, we used a histone-focused version of the super-SILAC approach to analyze and quantify hPTMs in breast cancer samples. Based on their different subtypes, grade of aggressiveness and hPTM patterns (supplemental Fig. S4), we chose four breast cancer cell lines to generate a super-SILAC mix that was spiked into breast cancer samples prior to GeLC-MS analysis and used as an internal reference (Fig. 3*A*).

##### Application of PAT-H-MS in Combination with Super-SILAC to Accurately Quantify hPTM Changes in Human Breast Cancer FFPE Samples

We applied the PAT-H-MS protocol to the analysis of FFPE breast tumor samples from three patients and compared them to their respective fresh frozen tissues as control (See supplemental Fig. S3*A*–S3*C* for the molecular characterization of the samples, the SDS-PAGE gel used to assess the sample purity and the list of the identified peptides). We obtained histones from frozen breast cancer tissue by developing a simple extraction protocol that yields fairly pure histones (∼75% of the sample based on protein concentration) in amounts sufficient for MS analysis from small quantities of starting frozen material (see Experimental Procedures). Confirming the results obtained with mouse samples, the quantification of the FFPE and the corresponding frozen tissues showed remarkably similar results for histone H3 peptides 3–8, 9–17, 18–26 and 27–40 (supplemental Fig. S3*D*–S3*E*).

Next, we combined the PAT-H-MS and the super-SILAC approaches. The output of this type of experiment is represented by L/H ratios that are a measure of the abundance of the peptides in each breast cancer primary specimen (light channel), compared with the super-SILAC mix (heavy channel). Such ratios were then divided by the average ratio to better visualize differences among samples (see Experimental Procedures and supplemental Data set S2). Ratios, and correspondingly hPTM changes among patients, were remarkably similar for the two sets of samples that were either frozen or stored in paraffin (*r* = 0.72, *p* < 0.0001, Fig. 3*B*–3*C*). The majority of the trends were maintained in frozen and FFPE samples, although a few differences existed, which may also be ascribed to the fact that distinct tissue portions were frozen or fixed. Indeed, the heterogeneity of tumor tissues represents an intrinsic limitation for the analysis of both fresh-frozen and FFPE tissue, which could be overcome by combining the PAT-H-MS protocol with procedures such as sampling of tumor cores or laser microdissection, to increase sample homogeneity. Recently, these techniques have been successfully used for the implementation of pathology tissue chromatin immunoprecipitation (PAT-ChIP), an approach for the extraction and high-throughput genomic analysis of FFPE-derived chromatin that represents the genomic counterpart of our proteomic analysis (6, 7).

##### Histone Modification Profiling of Breast Cancer Subtypes by PAT-H-MS

**Fig. 4.**
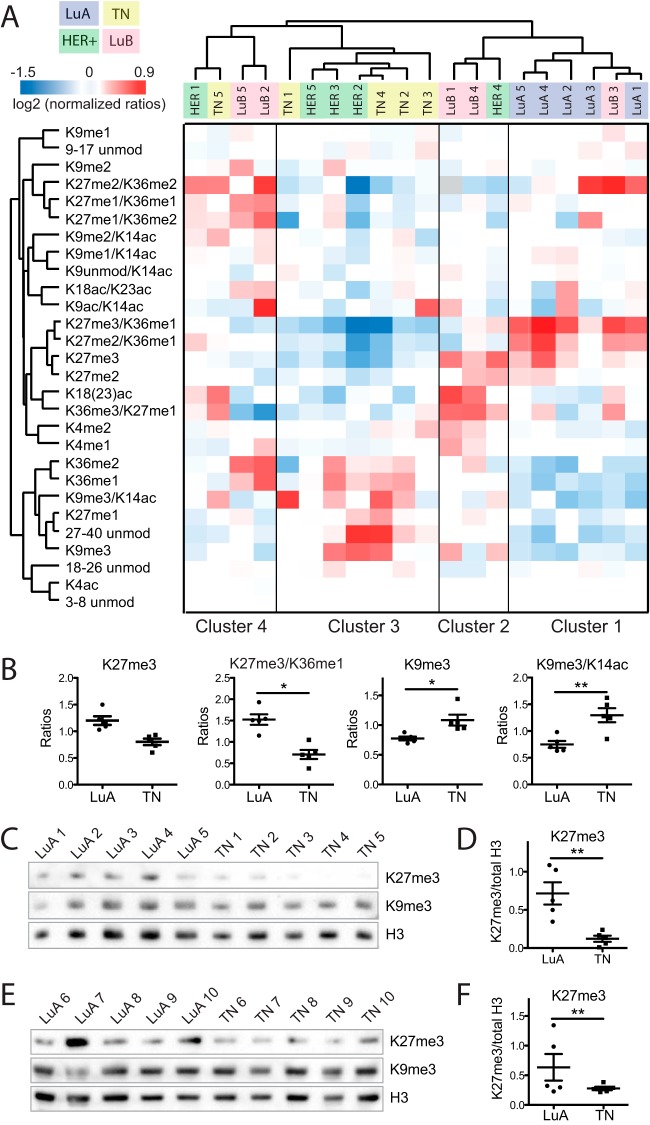
**Epigenetic profiling of breast cancer subtypes by PAT-H-MS.** *A*, Heatmap display of the log_2_ of ratios obtained for the indicated hPTMs for FFPE breast cancer biopsies belonging to four different subtypes (LuA: Luminal A-like, LuB: Luminal B-like, TN: Triple Negative, HER: HER-positive). L/H relative abundances ratios obtained with the super-SILAC strategy (light channel: breast cancer biopsy, heavy channel: spike-in super-SILAC standard) normalized over the average value across the samples are shown. *B*, Ratios obtained for the indicated peptides containing K27me3 or K9me3 in Luminal A or Triple negative breast cancer samples. *C*, *D*, FFPE extracts from the Luminal A and Triple Negative samples analyzed in *A–B* were probed by immunoblot with anti-H3K27me3, H3K9me3 and total H3 antibodies. *D*, The levels of H3K27me3 were quantified from the immunoblots shown in *C* and normalized to the amount of total histone H3. *E*, *F*, Extracts from additional frozen Luminal A and Triple Negative samples were probed by immunoblot and quantified as in *C–D*. Samples in *B* were compared by one-way ANOVA and Bonferroni's post-hoc test. Samples in D and F were compared by T test. Error bars represent standard error from 5 patient samples. *p < 0.05, **p < 0.01.

Breast cancer can be divided clinically into four main subtypes: Luminal A-like and Luminal B-like (expressing ER and PR), HER2-positive, and Triple Negative. We asked whether breast cancer tumors show distinct hPTM patterns and whether a relationship between those patterns and clinical features exist. We quantitated 24 differentially modified peptides from histone H3 in FFPE bioptic samples from patients matched for other clinical features (age, sex, etc.) belonging to the various subtypes (5 patients/subtype, supplemental Table S3). Non-supervised clustering defined four main clusters characterized by different levels of histone marks (Fig. 4). Cluster 1, containing mostly Luminal A-like samples, shows elevated levels of histone H3 K27me3, K27me3/K36me1, and K27me2/K36me1 and low levels of K9me3, K9me3/K14ac, K36me1, K36me2, and K27me1. Association between elevated K27me3 and Luminal A-like tumors has been reported in several immunohistochemistry (IHC) studies (31, 32), thus providing an indirect validation for our method. Cluster 3, which contains four out of five of the Triple Negative samples and three HER+ samples, shows opposite trends for these modifications. Two additional clusters (Cluster 2 and 4) comprise four Luminal B-like samples, two HER+ samples and one Triple Negative. Similarly to Cluster 1, Cluster 2 shows increased levels of K27me2 and me3, but in addition also presents elevated levels of peptides containing acetylation either on K18 or K23 (K18(23)ac) and K27me1/K36me3, whereas Cluster 3 has higher levels of K27me1/K36me1 and K27me2/K36me2 compared with the other clusters, with intermediated levels of K27me3. A more in-depth analysis revealed a remarkable difference in hPTM levels in particular among Luminal A-like and Triple Negative breast cancer samples (Fig. 4*B* and supplemental Fig. S6). We analyzed the FFPE extracts corresponding to these two subtypes (LuA 1–5 and TN 1–5) by Western blot analysis (Fig. 4*C*–4*D*), confirming the lower levels of K27me3 in Triple Negative samples through a different methodology. A similar trend was observed also by IHC analysis of the same samples (supplemental Fig. S7) and in additional frozen samples belonging to the same subtypes (Fig. 4*E*–4*F*). Interestingly, no significant differences were observed in any of these antibody-based assays for the K9me3 mark, for which PAT-H-MS showed quantitative differences of smaller amplitude (Fig. 4 and data not shown). We surmise that the MS-based method developed here may be more powerful in detecting small differences among samples compared with traditional antibody-based assays, that are by nature semi-quantitative and unable to detect faithfully small differences among samples, and that the observed difference in this residue measured by PAT-H-MS reflects a subtle, but real difference among samples.

## DISCUSSION

Drugs targeting enzymes able to modify chromatin by modifying histones are now in clinical use, and several more are at a clinical stage of drug development. A better understanding of the molecular mechanisms underlying epigenetic alterations in tumors is of great relevance to optimize the use of these drugs. To achieve this goal, better tools to explore the epigenome (globally and locally) have to be developed, to access efficiently patient samples.

Our results demonstrate for the first time the feasibility to perform a comprehensive, unbiased and quantitative MS-analysis of histone PTM patterns on patient samples prepared for routine pathology investigations, which had been restricted to antibody-based methods so far. Comparisons with fresh frozen-tissues show that most of the commonly analyzed histone H3 lysine acetylations and methylations are qualitatively and quantitatively preserved in FFPE tissues up to several years old, with the exception of histone H3 peptide 73–83. Few formalin-induced lysine modifications, mostly methylations, appear in FFPE samples stored for 6 years. However, the majority of these modifications represent a minor fraction of the total and do not substantially affect the relative quantification of the most common modifications found on histone H3 peptides 3–8, 9–17, 18–26, and 27–40, on which we focused our analyses. Therefore, our extraction protocol allows the identification and quantification of at least 24 distinct histone peptide forms, even after prolonged storage of FFPE samples. Because mild changes may be observed after 6 years of storage in paraffin also in a few of the 24 modified histone forms that we focused on, the detection of small differences (<1.5 fold) among samples may benefit from selecting samples from “storage age-matched” specimens. Importantly, the required starting material is absolutely compatible with what is available from most biobanks, further supporting its possible implementation in clinical research/use.

The PAT-H-MS approach provides several advantages compared with the traditional antibody-based methods normally employed in the clinic for hPTM analysis. First, tens of modifications can be detected in a single MS run, including modifications for which reliable antibodies do not exist. This also reduces the amount of starting material needed to obtain a comprehensive view of hPTM patterns. Second, hPTMs can be accurately quantified, particularly when spike-in standards, such as a super-SILAC mix, are used as internal reference. Using an internal standard also facilitates sample multiplexing and allows to reliably analyze samples even over long periods of time, a conceivable scenario when testing a large clinical dataset. Finally, PAT-H-MS can detect combinations of modifications, which may help defining modification signatures of pathological states with improved predictive power as compared with single biomarkers.

As a proof-of-principle demonstration, we used the PAT-H-MS approach in combination with a super-SILAC experimental set-up to profile hPTM changes in a clinical collection of human breast cancer biopsies belonging to different subtypes. In line with previous studies (31, 32), we observed that histone H3 K27me3, alone or in combination with K36me1, was higher in Luminal A-like samples compared with other subtypes and was particularly low in samples belonging to Cluster 3, which contains Triple Negative and HER2 samples. This result provides the proof of concept that PAT-H-MS can be used to extract clinically relevant information from patient samples. Most of the Triple Negative samples were grouped in Cluster 3. In addition to a decreased level in K27me3-containing peptides, this cluster showed an increase of K9me3, alone or in combination with K14ac. Interestingly, the samples belonging to Cluster 3 have the highest levels of Ki67 expression among the samples tested (supplemental Table S3), possibly linking the hPTM signature observed in this group with highly proliferating tumors. Consistent with this hypothesis, the only Triple Negative sample that falls into a different cluster has a much lower Ki67 expression than the other samples belonging to the same subtype (supplemental Table S3).

Triple-negative breast cancers represent a highly diverse group of cancers, for which well-defined molecular targets have not yet been identified and targeted therapies do not exist. Given the heterogeneity of this group of cancers, it was somewhat surprising to observe that four out of five of the Triple Negative samples that we analyzed were rather homogeneous in their hPTM patterns, and in particular showed lower levels of the K27me3 mark and increased levels of the K9me3 and K36me1 marks. The analysis of a larger cohort of breast cancer patients is ongoing, and will determine whether the histone marks found in this study can help distinguishing Triple Negative samples from other breast cancer subtypes. In addition, because altered levels of histone marks are often associated with deregulation of histone modifying enzymes, our findings may suggest candidate epigenetic targets for the treatment of Triple Negative cancers. For instance, the elevated levels of K9me3 and K36me1/me2 that we observed may be linked to the deregulation of members of the JMJD2 cluster, which demethylate both of these marks (33).

The set-up used in this experiment could be also readily employed in extended patients cohorts to discover epigenetic marks linked with patient outcome, cancer staging or other clinical features in breast cancer as well as any other diseases where epigenetic components might play a role. In conclusion, by combining the strengths of MS-based hPTM analysis with the vast clinical information associated with FFPE samples, PAT-H-MS represents a significant technological advancement in the field of clinical epigenetics. We thus envision that it will contribute to epigenetic and clinical research by facilitating the identification of epigenetic biomarkers potentially useful for the management of cancer and other diseases.

## Supplementary Material

Supplemental Data

## Footnotes

^1^ The abbreviations used are:

hPTM: histone post-translational modifications
PAT-H-MS FFPE: pathology tissue analysis of histones by mass spectrometry
FFPE: formalin-fixed paraffin embedded
MS/MS: tandem mass spectrometry
RA: relative abundance
XIC: Extracted ion chromatograms
SDS-PAGE: sodium dodecyl sulphate-polyacrylamide gel electrophoresis
geLC-MS: SDS-PAGE followed by in-gel digestion and peptide analysis by mass spectrometry
SILAC: stable isotope labeling by amino acid in cell culture
Arg0: unlabeled L-arginine
Arg10: ^13^C_6_^15^N_4_ L-arginine
Lys0: unlabeled L-lysine
Lys8: ^13^C_6_^15^N_2_ L-lysine
FDR: false discovery rate
H: heavy-labeled
L: light-labeled.

## References

[1] Jenuwein T., and Allis C. D. (2001) Translating the histone code. Science 293, 1074–1080 11498575 10.1126/science.1063127

[2] Portela A., and Esteller M. (2010) Epigenetic modifications and human disease. Nat. Biotechnol. 28, 1057–1068 20944598 10.1038/nbt.1685

[3] Seligson D. B., Horvath S., Shi T., Yu H., Tze S., Grunstein M., and Kurdistani S. K. (2005) Global histone modification patterns predict risk of prostate cancer recurrence. Nature 435, 1262–1266 15988529 10.1038/nature03672

[4] Seligson D. B., Horvath S., McBrian M. A., Mah V., Yu H., Tze S., Wang Q., Chia D., Goodglick L., and Kurdistani S. K. (2009) Global levels of histone modifications predict prognosis in different cancers. Am. J. Pathol. 174, 1619–1628 19349354 10.2353/ajpath.2009.080874 PMC2671251

[5] Soldi M., Cuomo A., Bremang M., and Bonaldi T. (2013) Mass spectrometry-based proteomics for the analysis of chromatin structure and dynamics. Int. J. Mol. Sci. 14, 5402–5431 23466885 10.3390/ijms14035402 PMC3634404

[6] Amatori S., Ballarini M., Faversani A., Belloni E., Fusar F., Bosari S., Pelicci P. G., Minucci S., and Fanelli M. (2014) PAT-ChIP coupled with laser microdissection allows the study of chromatin in selected cell populations from paraffin-embedded patient samples. Epigenetics Chromatin 7, 18 25104973 10.1186/1756-8935-7-18 PMC4124777

[7] Fanelli M., Amatori S., Barozzi I., Soncini M., Dal Zuffo R., Bucci G., Capra M., Quarto M., Dellino G. I., Mercurio C., Alcalay M., Viale G., Pelicci P. G., and Minucci S. (2010) Pathology tissue-chromatin immunoprecipitation, coupled with high-throughput sequencing, allows the epigenetic profiling of patient samples. Proc. Natl. Acad. Sci. U.S.A. 107, 21535–21540 21106756 10.1073/pnas.1007647107 PMC3003125

[8] Palmer-Toy D. E., Krastins B., Sarracino D. A., Nadol J. B. Jr., and Merchant S. N. (2005) Efficient method for the proteomic analysis of fixed and embedded tissues. J. Proteome Res. 4, 2404–2411 16335994 10.1021/pr050208p

[9] Fowler C. B., O'Leary T. J., and Mason J. T. (2013) Toward improving the proteomic analysis of formalin-fixed, paraffin-embedded tissue. Expert Rev. Proteomics 10, 389–400 23992421 10.1586/14789450.2013.820531

[10] Tian Y., Gurley K., Meany D. L., Kemp C. J., and Zhang H. (2009) N-linked glycoproteomic analysis of formalin-fixed and paraffin-embedded tissues. J. Proteome Res. 8, 1657–1662 19714870 10.1021/pr800952h PMC2975740

[11] Ostasiewicz P., Zielinska D. F., Mann M., and Wisniewski J. R. (2010) Proteome, phosphoproteome, and N-glycoproteome are quantitatively preserved in formalin-fixed paraffin-embedded tissue and analyzable by high-resolution mass spectrometry. J. Proteome Res. 9, 3688–3700 20469934 10.1021/pr100234w

[12] Wakabayashi M., Yoshihara H., Masuda T., Tsukahara M., Sugiyama N., and Ishihama Y. (2014) Phosphoproteome analysis of formalin-fixed and paraffin-embedded tissue sections mounted on microscope slides. J. Proteome Res. 13, 915–924 24328109 10.1021/pr400960r

[13] Soldi M., Cuomo A., and Bonaldi T. (2014) Improved bottom-up strategy to efficiently separate hypermodified histone peptides through ultra-HPLC separation on a bench top Orbitrap instrument. Proteomics 14, 2212–2225 25073962 10.1002/pmic.201400075

[14] Minucci S., Monestiroli S., Giavara S., Ronzoni S., Marchesi F., Insinga A., Diverio D., Gasparini P., Capillo M., Colombo E., Matteucci C., Contegno F., Lo-Coco F., Scanziani E., Gobbi A., and Pelicci P. G. (2002) PML-RAR induces promyelocytic leukemias with high efficiency following retroviral gene transfer into purified murine hematopoietic progenitors. Blood 100, 2989–2995 12351412 10.1182/blood-2001-11-0089

[15] Cuomo A., Moretti S., Minucci S., and Bonaldi T. (2011) SILAC-based proteomic analysis to dissect the “histone modification signature” of human breast cancer cells. Amino Acids 41, 387–399 20617350 10.1007/s00726-010-0668-2

[16] Cox J., Neuhauser N., Michalski A., Scheltema R. A., Olsen J. V., and Mann M. (2011) Andromeda: a peptide search engine integrated into the MaxQuant environment. J. Proteome Res. 10, 1794–1805 21254760 10.1021/pr101065j

[17] Ong S. E., Mittler G., and Mann M. (2004) Identifying and quantifying in vivo methylation sites by heavy methyl SILAC. Nat. Methods 1, 119–126 15782174 10.1038/nmeth715

[18] Bremang M., Cuomo A., Agresta A. M., Stugiewicz M., Spadotto V., and Bonaldi T. (2013) Mass spectrometry-based identification and characterisation of lysine and arginine methylation in the human proteome. Mol. Biosyst. 9, 2231–2247 23748837 10.1039/c3mb00009e

[19] Zhang Y., Muller M., Xu B., Yoshida Y., Horlacher O., Nikitin F., Garessus S., Magdeldin S., Kinoshita N., Fujinaka H., Yaoita E., Hasegawa M., Lisacek F., and Yamamoto T. (2015) Unrestricted modification search reveals lysine methylation as major modification induced by tissue formalin fixation and paraffin embedding. Proteomics 15, 2568–2579 25825003 10.1002/pmic.201400454

[20] Jung H. R., Pasini D., Helin K., and Jensen O. N. (2010) Quantitative mass spectrometry of histones H3.2 and H3.3 in Suz12-deficient mouse embryonic stem cells reveals distinct, dynamic post-translational modifications at Lys-27 and Lys-36. Mol. Cell. Proteomics 9, 838–850 20150217 10.1074/mcp.M900489-MCP200 PMC2871418

[21] Pan C., Gnad F., Olsen J. V., and Mann M. (2008) Quantitative phosphoproteome analysis of a mouse liver cell line reveals specificity of phosphatase inhibitors. Proteomics 8, 4534–4546 18846507 10.1002/pmic.200800105

[22] Olsen J. V., Blagoev B., Gnad F., Macek B., Kumar C., Mortensen P., and Mann M. (2006) Global, in vivo, and site-specific phosphorylation dynamics in signaling networks. Cell 127, 635–648 17081983 10.1016/j.cell.2006.09.026

[23] Vizcaino J. A., Deutsch E. W., Wang R., Csordas A., Reisinger F., Rios D., Dianes J. A., Sun Z., Farrah T., Bandeira N., Binz P. A., Xenarios I., Eisenacher M., Mayer G., Gatto L., Campos A., Chalkley R. J., Kraus H. J., Albar J. P., Martinez-Bartolome S., Apweiler R., Omenn G. S., Martens L., Jones A. R., and Hermjakob H. (2014) ProteomeXchange provides globally coordinated proteomics data submission and dissemination. Nat. Biotechnol. 32, 223–226 24727771 10.1038/nbt.2839 PMC3986813

[24] Pesavento J. J., Mizzen C. A., and Kelleher N. L. (2006) Quantitative analysis of modified proteins and their positional isomers by tandem mass spectrometry: human histone H4. Anal. Chem. 78, 4271–4280 16808433 10.1021/ac0600050

[25] Bonaldi T., Regula J. T., and Imhof A. (2004) The use of mass spectrometry for the analysis of histone modifications. Methods Enzymol. 377, 111–130 14979021 10.1016/S0076-6879(03)77006-2

[26] Ong S. E., Blagoev B., Kratchmarova I., Kristensen D. B., Steen H., Pandey A., and Mann M. (2002) Stable isotope labeling by amino acids in cell culture, SILAC, as a simple and accurate approach to expression proteomics. Mol. Cell. Proteomics 1, 376–386 12118079 10.1074/mcp.m200025-mcp200

[27] Geiger T., Cox J., Ostasiewicz P., Wisniewski J. R., and Mann M. (2010) Super-SILAC mix for quantitative proteomics of human tumor tissue. Nat. Methods 7, 383–385 20364148 10.1038/nmeth.1446

[28] Monetti M., Nagaraj N., Sharma K., and Mann M. (2011) Large-scale phosphosite quantification in tissues by a spike-in SILAC method. Nat. Methods 8, 655–658 21743459 10.1038/nmeth.1647

[29] Boersema P. J., Geiger T., Wisniewski J. R., and Mann M. (2013) Quantification of the N-glycosylated secretome by super-SILAC during breast cancer progression and in human blood samples. Mol. Cell. Proteomics 12, 158–171 23090970 10.1074/mcp.M112.023614 PMC3536897

[30] Jaffe J. D., Wang Y., Chan H. M., Zhang J., Huether R., Kryukov G. V., Bhang H. E., Taylor J. E., Hu M., Englund N. P., Yan F., Wang Z., Robert McDonald E. 3rd, Wei L., Ma J., Easton J., Yu Z., deBeaumount R., Gibaja V., Venkatesan K., Schlegel R., Sellers W. R., Keen N., Liu J., Caponigro G., Barretina J., Cooke V. G., Mullighan C., Carr S. A., Downing J. R., Garraway L. A., and Stegmeier F. (2013) Global chromatin profiling reveals NSD2 mutations in pediatric acute lymphoblastic leukemia. Nat. Genet. 45, 1386–1391 24076604 10.1038/ng.2777 PMC4262138

[31] Holm K., Grabau D., Lovgren K., Aradottir S., Gruvberger-Saal S., Howlin J., Saal L. H., Ethier S. P., Bendahl P. O., Stal O., Malmstrom P., Ferno M., Ryden L., Hegardt C., Borg A., and Ringner M. (2012) Global H3K27 trimethylation and EZH2 abundance in breast tumor subtypes. Mol. Oncol. 6, 494–506 22766277 10.1016/j.molonc.2012.06.002 PMC5528390

[32] Healey M. A., Hu R., Beck A. H., Collins L. C., Schnitt S. J., Tamimi R. M., and Hazra A. (2014) Association of H3K9me3 and H3K27me3 repressive histone marks with breast cancer subtypes in the Nurses' Health Study. Breast Cancer Res. Treat 147, 639–651 25224916 10.1007/s10549-014-3089-1

[33] Cloos P. A., Christensen J., Agger K., and Helin K. (2008) Erasing the methyl mark: histone demethylases at the center of cellular differentiation and disease. Genes Dev. 22, 1115–1140 18451103 10.1101/gad.1652908 PMC2732404

