# Supplementary material for: Pathology Tissue-quantitative Mass Spectrometry Analysis to Profile Histone Post-translational Modification Patterns in Patient Samples
Source: Mol Cell Proteomics. 2015 Oct 13;15(3):866–77. doi: 10.1074/mcp.M115.054510 (PMC4813706; doi:10.1074/mcp.M115.054510)
Supplement: Supplemental Data [file supp_15_3_866__index.html]

Pathology tissue-quantitative mass spectrometry analysis to profile histone post-translational modification patterns in patient samples — Pathology Tissue-quantitative Mass Spectrometry Analysis to Profile Histone Post-translational Modification Patterns in Patient Samples — Pathology Tissue Quantitative MS Analysis of Histone PTMs — Supplemental Data 

# Pathology Tissue-quantitative Mass Spectrometry Analysis to Profile Histone Post-translational Modification Patterns in Patient Samples

## Supplemental Data

- supplementary figures and tables (.pdf, 1.6 MB) - supplementary figures and tables
- Dataset S1 (.xlsx, 45 KB) - Dataset S1
- Dataset S2 (.xlsx, 121 KB) - Dataset S2
- Dataset S3 (.xlsx, 93 KB) - Dataset S3
- Table S4 (.xlsx, 16 KB) - Table S4
- Figure S8 (.pdf, 7.6 MB) - Figure S8 (previously submitted as Figure S7)
