# Supplementary material for: Pathology Tissue-quantitative Mass Spectrometry Analysis to Profile Histone Post-translational Modification Patterns in Patient Samples
Source: Mol Cell Proteomics. 2015 Oct 13;15(3):866–77. doi: 10.1074/mcp.M115.054510 (PMC4813706; doi:10.1074/mcp.M115.054510)
Supplement: Supplemental Data [file 10.1074_M115.054510_mcp.M115.054510-1.pdf]

Pathology tissue-quantitative mass spectrometry analysis to profile histone post-translational modification patterns in patient samples

Roberta Noberini, Andrea Uggetti, Giancarlo Pruneri, Saverio Minucci
and Tiziana Bonaldi

Supplemental Data

Figure S1

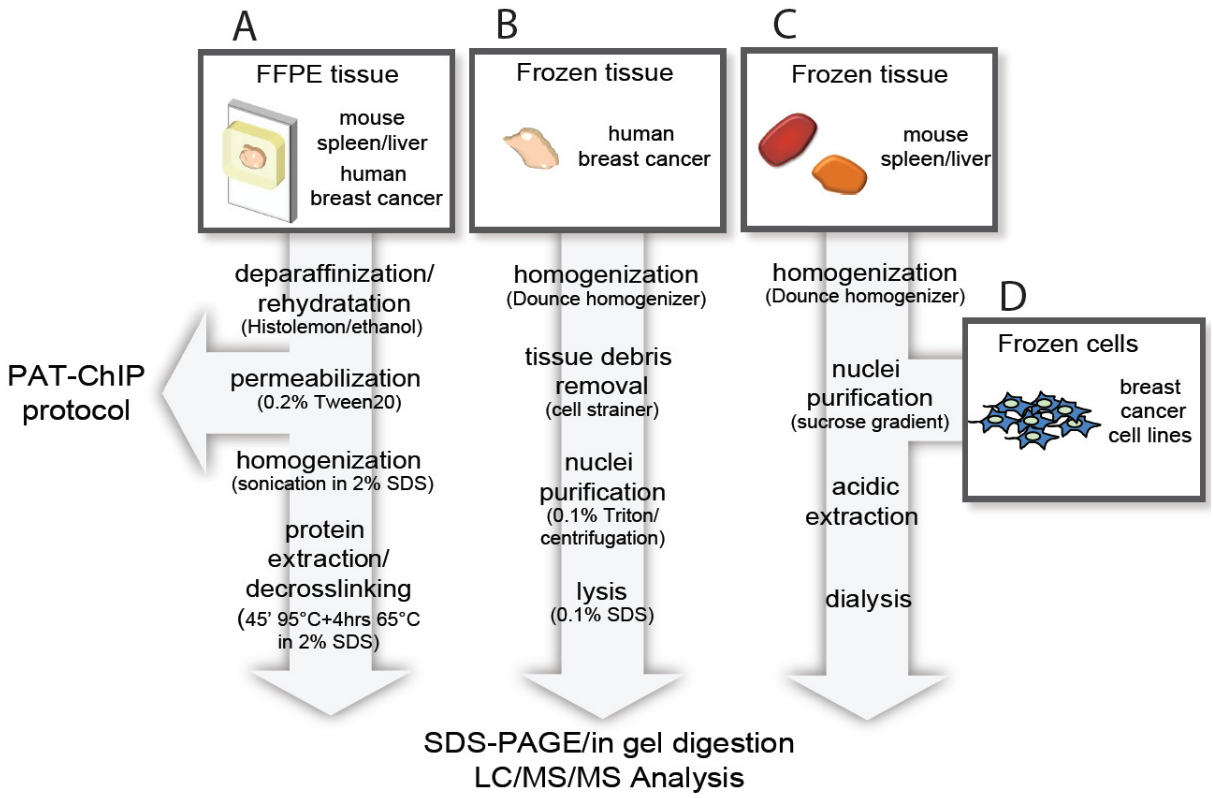

Figure S1. Scheme of protocols used to isolate histones from different types of samples. (A) FFPE tissues (mouse and human). After the permeabilization step, the PAT-ChIP protocol (Fanelli et al. 2010, *PNAS*) can also be performed, since the initial steps are shared between the two protocols. (B) Frozen human breast cancer tissue. (C) Frozen mouse liver and spleen. (D) Breast cancer cell lines.

Figure S2

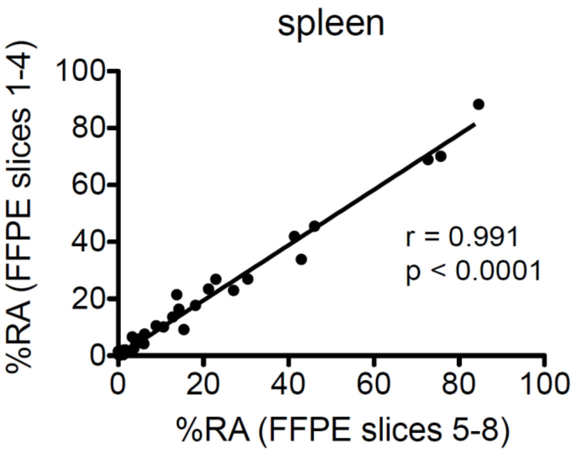

Figure S2. hPTM % Relative abundance (%RA) correlation between different FFPE sections from the same spleen sample. Correlation of %RA calculated for mouse spleen histones obtained from adjacent FFPE slices. Pearson correlation coefficients (r) and p-values are shown.

Figure S3

A

Breast cancer biopsy #	Subtype [1]	Immunoprofile [2]				pTNM [3]	Differentiation
		ER	PR	HER2	Ki-67		
1	LuB	+	+	-	25%	pT2N1a	moderate
2	LuA	+	+	-	17%	pT1cN0M0	moderate
3	LuB	+	-	-	22%	pT1cN0M0	poor

[1] Lu: luminal, Ba: Basal, based Ki-67 labeling index value
[2] ER: estrogen receptor, PR: progesteron receptor, HER2: human epidermal growth factor receptor 2
[3] pTNM: pathological tumor-node-metastasis

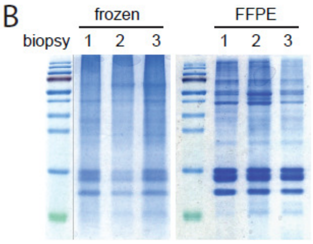

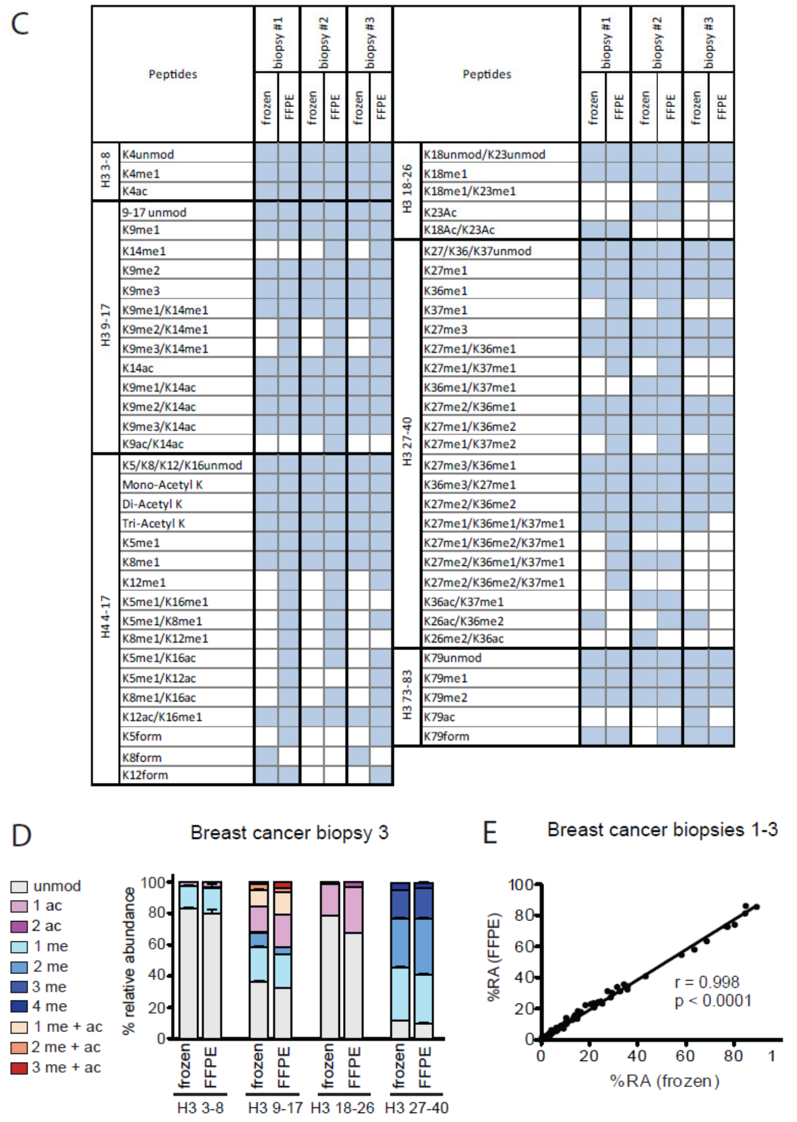

Figure S3. (A) Molecular classification of breast cancer biopsies 1-3. (B) Coomassie-staining visualization of histones purified from FFPE and frozen breast cancer samples. Twenty µg of FFPE extracts and 4 µg of histones obtained from frozen biopsies were loaded on a 17% gel. Non-relevant lanes were removed. (C) List of H3 and H4 peptides identified from frozen or FFPE samples for breast cancer human biopsies 1-3. (D) Percent relative abundances (%RA) profiles for H3 modified peptides from frozen or FFPE samples for human breast cancer biopsy #3. Error bars represent the standard error from triplicate measurements. (E) Correlation of %RA values calculated for FFPE and fresh-frozen tissues from three human breast cancer biopsies. The Pearson correlation coefficient (r) and p-value are shown.

Figure S4

A

Cell line	Subtype	Immunoprofile ER	PR	HER2
MDA-MB-453	Uncl	-	-	-
MCF7	LuA	+	+	-
MDA-MB-361	LuB	+	+ / -	+
MDA-MB-468	BaB	-	-	-
MDA-MB-231	BaA	-	-	-

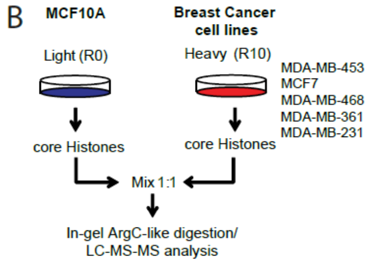

C

L/H ratios	MDA-MB-453		MCF7		MDA-MB-468		MDA-MB-361		MDA-MB-231	
	1	2	1	2	1	2	1	2	1	2
H3 3-8 unmod	1.00	0.98	0.98	0.97	0.98	0.99	0.93	0.91	0.94	0.98
H3 K4me1	0.96	1.06	1.12	1.23	1.07	1.04	1.41	1.52	1.33	1.07
H3 K4me2	1.34	1.28	1.29	1.26	1.28	1.26	1.52	1.34	1.92	1.42
H3 K4ac	1.00	0.90	0.97	0.93	1.11	0.98	0.92	0.91	1.23	0.96
H3 9-17 unmod	0.90	0.91	1.31	1.26	1.23	1.23	0.78	0.73	0.79	0.77
H3 K9me1	0.99	0.94	1.14	1.12	1.21	1.19	1.03	0.95	1.11	1.11
H3 K9me2	1.42	1.31	1.30	1.35	1.39	1.39	1.55	1.41	1.91	2.02
H3 K9me3	0.92	1.00	1.15	1.10	1.07	1.12	1.16	1.07	2.47	2.44
H3 K14ac	0.69	0.66	0.69	0.62	0.68	0.66	0.65	0.58	0.42	0.42
H3 K9me1/K14ac	0.83	0.78	0.62	0.58	0.62	0.60	0.90	0.80	0.54	0.58
H3 K9me2/K14ac	1.27	1.26	0.75	0.69	0.61	0.60	1.38	1.30	1.01	1.06
H3 K9me3/K14ac	1.49	1.09	0.93	0.68	0.63	0.49	1.97	1.38	2.08	1.03
H3 K9ac/K14ac	1.06	1.10	0.83	0.82	0.84	1.10	1.11	1.11	0.76	0.80
H3 18-26 unmod	1.08	0.90	0.96	1.02	0.93	1.02	0.97	1.00	0.87	0.93
H3 K18me1	1.64	1.29	1.80	1.77	1.06	1.16	1.55	1.47	1.92	2.01
H3 K18(23)ac	0.59	1.41	1.10	0.86	1.29	0.83	0.98	0.87	1.52	1.28
H3 K18ac/K23ac	2.83	2.21	1.40	1.43	1.59	2.19	3.79	4.32	1.85	2.10
H3 27-40 unmod	0.51	0.43	0.67	0.62	1.08	1.07	0.60	0.62	0.38	0.31
H3 K27me1	0.82	0.68	1.14	0.95	1.45	1.25	0.65	0.79	0.69	0.56
H3 K27me2	1.69	1.54	2.97	2.87	2.02	1.89	1.15	1.18	2.23	1.50
H3 K36me2	0.53	0.45	0.52	0.55	0.94	0.92	1.17	1.16	0.73	0.72
H3 K27me	0.99	1.27	1.65	1.49	1.14	1.04	1.35	1.19	2.49	1.34
H3 K27me2/K36me1	1.55	1.31	1.15	1.11	0.60	1.23	1.21	0.85	1.04	1.22
H3 K27me1/K36me2	0.83	0.72	0.51	0.48	0.72	0.68	1.03	0.99	0.85	
H3 K27me1/K36me1	0.96	0.89	0.78	0.75	0.76	0.74	1.03	1.05	0.76	0.64
H3 K27me2/K36me2	1.14	1.07	0.47	0.50	0.46	0.47	1.25	1.25	1.14	0.99
H3 K27me3/K36me1	0.87	0.91	1.14	1.14	0.35	0.50	0.95	1.18	1.52	0.95
H3 K27me3/K36me2	0.62	1.35	0.64	0.72	0.35	0.67	0.92	1.15	1.12	
H3 K27me3/K36me2	0.84	1.24	0.63	0.67	0.18	0.40	1.43	1.31	1.41	1.28
H3 73-83 unmod	0.98	0.98	0.99	1.00	1.01	1.01	1.04	1.07	0.98	0.99
H3 K79me1	1.13	1.10	1.01	0.98	0.93	0.92	0.79	0.77	1.11	1.13
H3 K79me2	1.23	1.21	1.16	1.11	0.85	0.86	0.27	0.25	0.96	0.92
H3 K79ac	1.00	1.03	1.00	0.88	1.01	0.86	1.01	0.93	1.01	0.89

0.20
0.50
0.75
1.00
1.50
2.00
3.00
4.00

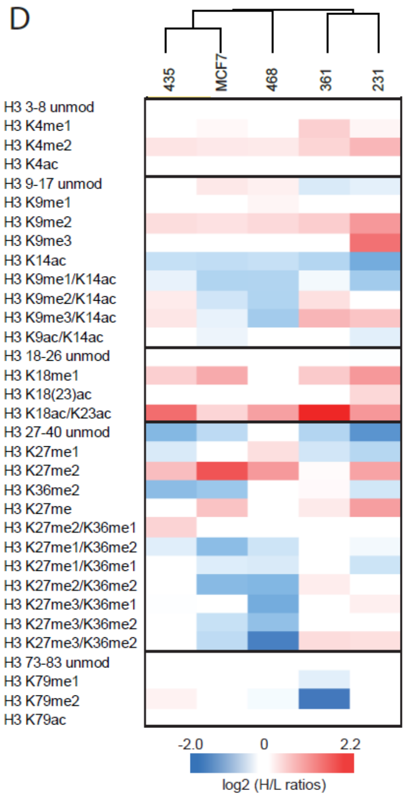

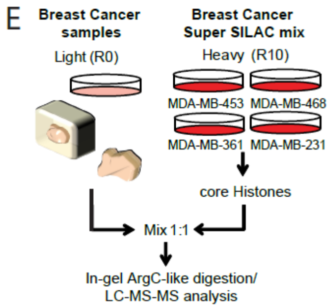

Figure S4. Set up of the super-SILAC approach for hPTM analysis. (A) Molecular classification of the breast cancer cell lines tested. Uncl: unclassified, Lu: luminal, Ba: Basal, ER: estrogen receptor, PR: progesteron receptor, HER2: human epidermal growth factor receptor 2 (B) Schematic rappresentation of the SILAC approach used to profile hPTMs in five heavy-labelled breast cancer cell lines using unlabelled normal breast MCF10 cells as spike-in. (C) H/L SILAC ratios for modified peptides were obtained from extracted ion chromatographic peaks for light and heavy peptides in duplicate experiments. Ratios from experiment 1 and 2 were obtained using an uHPLC system in combination with a Q Exactive instrument, or an HPLC system in combination with a LTQ-Velos Orbitrap instrument, respectively. (D) Heatmap display of log2(H/L) ratios for hPTMs in the five breast cancer cell lines and hierarchical clustering based on Euclidean distance. Based on their different modification patterns, MDA-MB-453, MDA-MB-361, MDA-MB-468 and MDA-MB-231 were selected to generate the spike-in super-SILAC standard for the analysis of breast cancer cell lines and primary samples (E).

Figure S5

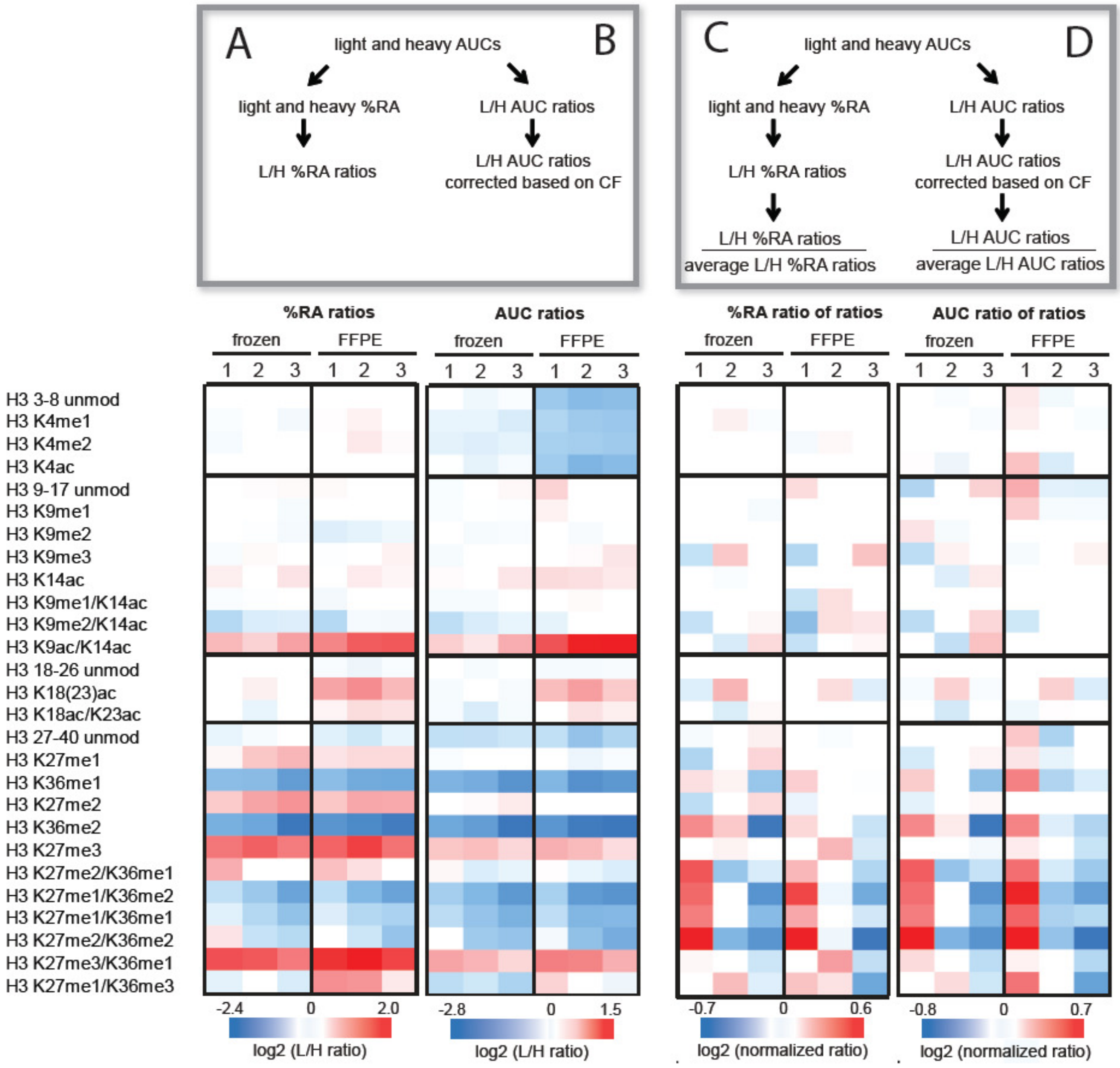

Figure S5. Comparison of ratios obtained from AUC and %RA values. Heatmap display of the $\log_2$ of ratios obtained for the indicated hPTMs for frozen and FFPE breast cancer biopsies (average from 3 technical replicates), using different normalization strategies (schematized in the top panels). L/H of % relative abundances (%RA) or area under the curve (AUC) values are shown in A and B, respectively. In B, L/H ratios were corrected based on the SILAC ratio of an unmodified peptide to account for mixing errors. In C and D the ratio shown in A and B were divided by the average ratio across all the samples analyzed (FFPE and frozen were averaged separately). AUC ratios for peptide 3-8 (shown in B) were lower in FFPE samples compared to frozen, likely due to lower extraction efficiency from paraffin, but %RA values were not affected.

Figure S6

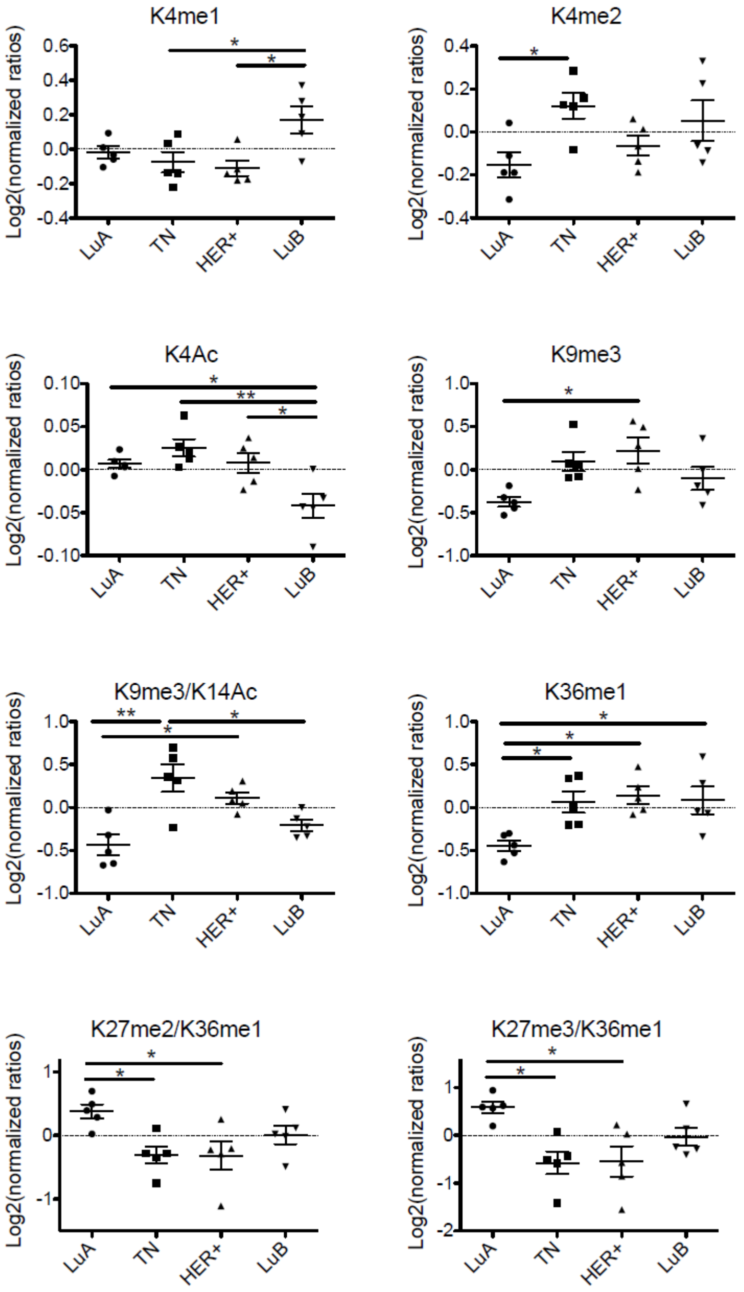

Figure S6. Ratios obtained for the indicated hPTMs in FFPE breast cancer biopsies belonging to different subtypes were compared by one-way ANOVA; only modified peptides showing significant changes are shown. Normalized ratios are the %RA ratios divided by the average ratio across all the samples analyzed. Error bars represent the standard error from 5 biological replicates. *p<0.05, **p<0.01 by one-way ANOVA and Bonferroni's post-hoc test.

Figure S7

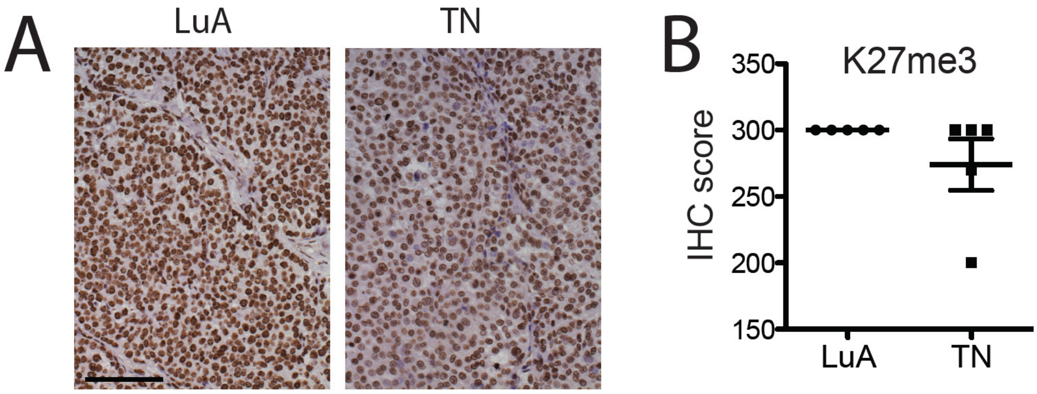

Figure S7. (A) Representative images from Luminal A-like and Triple negative FFPE sections corresponding to the samples analyzed in Figure 4 A-B, which were immunostained with an anti-H3K27me3 antibody (Millipore 07-449, 1:100) and H&E. (B) Immunostained sections were quantified based on the percentage and intensity of stained cells. The level of staining was evaluated by assigning to each image a score from 0 to 300, which corresponds to the percent of stained cells multiplied for the intensity of the staining (score 1, 2 or 3 for increasing intensity). Scalebar: 100 μm

Figure S8. (separate PDF file). Representative MS/MS spectra for all the identified modified histone peptides obtained from histone extraction and GeLC-MS analysis (parameters for these spectra are reported in Table S2).

Dataset S1 (separate Excel file). AUC and %RA values obtained for mouse samples (spleen, spleen 6 years and liver).

Dataset S2 (separate Excel file). AUCs, %RAs and ratios obtained for breast cancer biopsies 1-3.

Dataset S3 (separate Excel file). AUCs, %RAs and ratios obtained for the breast cancer biopsies shown in Figure 4.

Table S1. Histone yield from FFPE tissues

Tissue	Octamer yield (μM) [1]	n[2]
Spleen	70 ± 8.1[3]	4
Liver	16 ± 4.5	2
Breast	46 ± 29	25
Kidney	20	1
Heart	10	1

[1] based on comparison with known amounts of recombinant histone H3.1 on a Comassie-stained SDS-PAGE gel

[2] n = number of experiments, [3] average ± SE

Table S2. % Relative abundances of histone modified peptides in frozen and FFPE mouse tissue

	Peptide	spleen[1]		liver[2]		spleen 6 years[2]	
		Frozen	FFPE	Frozen	FFPE	Frozen	FFPE
Histone H3	3-8 unmod	91 ± 0.44	91 ± 0.14	89 ± 0.33	86 ± 0.07	85 ± 0.74	80 ± 0.44
	K4me1	7.5 ± 0.23	6.7 ± 0.31	7.3 ± 0.3	9.6 ± 0.08	9.2 ± 0.74	15 ± 0.55
	K4me2	0.1 ± 0.002	0.13 ± 0.02	0.19 ± 0.034	0.22 ± 0.03	0.51 ± 0.009	0.22 ± 0.06
	K4ac	1.2 ± 0.21	1.8 ± 0.14	4 ± 0.007	3.9 ± 0.01	4.7 ± 0.01	4.1 ± 0.04
	9-17 unmod	26 ± 2.2	26 ± 0.45	22 ± 0.12	20 ± 0.49	25 ± 0.02	20 ± 0.35
	K9me1 or K14me1	11 ± 0.07	8.9 ± 3.6	17 ± 0.49	16 ± 0.52	12 ± 0.04	12 ± 0.04
	K9me2	18 ± 1.1	18 ± 1.4	18 ± 0.5	16 ± 0.81	12 ± 0.06	11 ± 0.33
	K9me3	7.8 ± 0.26	7.5 ± 0.5	1.9 ± 0.017	3.3 ± 0.15	3.9 ± 0.05	4.3 ± 0.11
	K14ac	15 ± 0.55	16 ± 1.7	14 ± 0.11	15 ± 0.23	23 ± 0.14	23 ± 0.53
	K9me1/K14ac	7.5 ± 0.32	6.1 ± 2	12 ± 0.24	14 ± 0.16	11 ± 0.23	14 ± 0.04
	K9me2/K14ac	11 ± 0.94	12 ± 1.9	12 ± 0.22	13 ± 0.46	9.7 ± 0.13	10 ± 0.38
	K9me3/K14ac	3.3 ± 0.19	3.4 ± 0.5	0.82 ± 0.03	1.6 ± 0.002	1.8 ± 0.03	2.3 ± 0.08
	K9ac/K14ac	0.74 ± 0.01	1.2 ± 0.31	1.4 ± 0.01	1.9 ± 0.005	1.7 ± 0.004	2.9 ± 0.05
	K9me1/K14ac		0.7 ± 0.22				0.47 ± 0.0002
	K9me2/K14ac		0.09				0.5 ± 0.01
	K9me3/K14ac		0.04 ± 0.005				
	K9 form		0.03 ± 0.01				
	18-26 unmod	77 ± 0.82	74 ± 2.9	70 ± 0.36	67 ± 0.04	69 ± 0.25	60 ± 0.03
	K18ac or K23ac	21 ± 0.77	22 ± 3	27 ± 0.29	27 ± 0.06	28 ± 0.19	31 ± 0.01
	K18ac/K23ac	0.82 ± 0.094	1.2 ± 0.47	2.2 ± 0.04	2.5 ± 0.04	3 ± 0.05	4.2 ± 0.04
	K18me1/K23unmod	1 ± 0.039	2 ± 0.36	0.38 ± 0.02	2.7 ± 0.01	0.11 ± 0.005	2.2 ± 0.01
	K18me1/K23me1		0.16 ± 0.18				0.17 ± 0.0001
	K18form or K23form	0.05 ± 0.007	0.25 ± 0.06		0.47 ± 0.08	0.04 ± 0.001	1.7 ± 0.02
	73-83 unmod	92 ± 0.21	88 ± 1.8	78 ± 0.01	72 ± 1.1	88 ± 0.37	56 ± 1.1
	K79me1	5.7 ± 0.76	9.3 ± 2	16 ± 0.15	20 ± 1	6.9 ± 0.38	38 ± 0.98
	K79me2	0.72 ± 0.29	0.83 ± 0.28	2.7 ± 0.16	3.1 ± 0.06	0.71 ± 0.01	2.7 ± 0.24
	K79ac	1.1 ± 0.27	1.5 ± 0.36	2.9 ± 0.004	3 ± 0.08	4.4 ± 0.002	2.6 ± 0.12
	K79form	0.02 ±	0.44 ± 0.1		1.5 ± 0.1		
	27-40 unmod	22 ± 0.56	20 ± 1.7	6.8 ± 0.21	6 ± 0.19	22 ± 0.46	20 ± 0.59
	K27me1	34 ± 0.23	34 ± 0.22	23 ± 0.58	13 ± 0.37	49 ± 1.3	51 ± 2.3
	K36me1	2.5 ± 0.079	2.4 ± 0.27	1.3 ± 0.17	0.62 ± 0.026		
	K27me2	21 ± 2.2	22 ± 0.5	35 ± 0.88	37 ± 0.58	14 ± 0.12	11 ± 0.94
	K36me2	1 ± 0.51	1.1 ± 0.41			1.4 ± 0.006	1 ± 0.085
	K27me3	1.5 ± 0.22	1.4 ± 0.42	1.8 ± 0.1	6.1 ± 0.32	0.89 ± 0.009	0.6 ± 0.07
	K36me3	0.3 ± 0.13	0.29 ± 0.02	0.09 ± 0.007			
	K27me1/K36me1	5.6 ± 0.87	6.5 ± 0.21	3.4 ± 0.87	3.3 ± 0.34	5.5 ± 0.52	8.8 ± 1.1
	K27me1/K37me1		0.13 ± 0.04				
	K27me2/K36me1	5 ± 0.54	6.1 ± 0.02	24 ± 0.32	20 ± 0.2	2.9 ± 0.02	3.1 ± 0.34
	K27me1/K36me2	2 ± 0.68	2.2 ± 0.1	1.5 ± 0.01	4.1 ± 0.13	2.6 ± 0.13	1.6 ± 0.15
	K27me2/K37me1		0.08 ± 0.01			0.009 ± 0.001	0.39 ± 0.05
	K27me2/K36me2	1 ± 0.16	1.4 ± 0.92	1.6 ± 0.02	4.3 ± 0.19	0.46 ± 0.01	0.45 ± 0.12
	K27me3/K36me1	0.54 ± 0.07	0.5 ± 0.05	1.1 ± 0.06	4.6 ± 0.14	0.22 ± 0.002	0.21 ± 0.03
	K27me1/K36me2	0.32 ± 0.1	0.52 ± 0.27			0.41 ± 0.034	0.27 ± 0.03
	K27ac	0.27	0.11				
	K27me1/K36me1/K37me1						0.18 ± 0.006
	K27me1/K36me2/K37me1 or K27me1/K36me1/K37me2 or K27me2/K36me1/K37me1	4.3	1.2 ± 0.15			0.63 ± 0.04	0.71 ± 0.05
	K36form						0.88 ± 0.02
Histone H4	4-17 unmod	46 ± 1.9	13 ± 2.1	45 ± 0.05	42 ± 0.21	44 ± 0.05	27 ± 1.6
	Mono-Acetyl K	44 ± 0.51	44 ± 0.21	42 ± 0.007	43 ± 0.18	42 ± 0.05	35 ± 2.1
	Di-Acetyl K	8.7 ± 1	10 ± 0.74	11 ± 0.07	12 ± 0.01	11 ± 0.01	14 ± 0.87
	Tri-Acetyl K	1.3 ± 0.19	1.8 ± 0.11	1.8 ± 0.004	2.2 ± 0.01	1.9 ± 0.002	3.5 ± 0.25
	Tetra-Acetyl K	0.12 ± 0.023	0.27 ± 0.008	0.31 ± 0.01	0.38 ± 0.003	0.22 ± 0.0002	0.61 ± 0.06
	Mono-me	0.04 ± 0.022	0.49 ± 0.67			0.18 ± 0.003	12 ± 0.62
	K5me/1K16ac or K8me1/K16ac or K12ac/K16me1	0.05 ± 0.03	0.41 ± 0.56			0.13 ± 0.11	7.3 ± 5.6
	K5form or K8form or K12form	0.11 ± 0.15	0.26				

[1] average ± SE from biological replicates, [2] average ± SE from technical replicates

Table S3. Breast cancer biopsies

Breast cancer biopsy #	Subtype[1]	Immunoprofile[2]				Grading	Sample analyzed
		ER	PR	HER2	Ki-67		
LuA 1	Luminal A	95%	95%	-	15%	G2	FFPE
LuA 2	Luminal A	95%	95%	-	12%	G2	FFPE
LuA 3	Luminal A	90%	90%	80% (+)	18%	G2	FFPE
LuA 4	Luminal A	>95%	>95%	60% (+)	10%	G1	FFPE
LuA 5	Luminal A	90%	80%	30% (+)	13%	G2	FFPE
LuA 6	Luminal A	90%	90%	-	10%	G2	frozen
LuA 7	Luminal A	>95%	>95%	-	15%	G2	frozen
LuA 8	Luminal A	95%	95%	-	18%	G2	frozen
LuA 9	Luminal A	90%	90%	-	19%	G2	frozen
LuA 10	Luminal A	>95%	>95%	-	13%	G2	frozen
TN 1	Triple Negative	-	-	-	70%	G3	FFPE
TN 2	Triple Negative	-	-	-	75%	G3	FFPE
TN 3	Triple Negative	-	-	-	60%	G3	FFPE
TN 4	Triple Negative	-	-	-	70%	G3	FFPE
TN 5	Triple Negative	-	-	30% (+)	16%	G2	FFPE
TN 6	Triple Negative	-	-	-	68%	G3	frozen
TN 7	Triple Negative	-	-	20% (+)	85%	G3	frozen
TN 8	Triple Negative	-	-	15% (+)	70%	G3	frozen
TN 9	Triple Negative	-	-	-	70%	G3	frozen
TN 10	Triple Negative	-	-	30% (+)	90%	G3	frozen
HER 1	HER2 positive	-	-	90% (+++)	30%	G3	FFPE
HER 2	HER2 positive	-	-	>95% (+++)	85%		FFPE
HER 3	HER2 positive	-	-	>95% (+++)	46%	G3	FFPE
HER 4	HER2 positive	-	-	>95% (+++)	32%	G3	FFPE
HER 5	HER2 positive	-	-	>95% (+++)	75%	G3	FFPE
LuB 1	Luminal B	95%	20%	-	45%		FFPE
LuB 2	Luminal B	95%	80%	70% (++)	55%	G3	FFPE
LuB 3	Luminal B	95%	95%	65% (+)	25%	G3	FFPE
LuB 4	Luminal B	95%	10%	40% (+)	29%	G3	FFPE
LuB 5	Luminal B	95%	95%	60% (+)	26%	G3	FFPE

[1] Based on the Ki-67 labeling index value

[2] ER: estrogen receptor, PR: progesteron receptor, HER2: human epidermal growth factor receptor

Table S4 (separate Excel file). Parameters for representative histone modified peptides identified from FFPE samples (separate Excel file)
