# Supplementary material for: Pathology Tissue-quantitative Mass Spectrometry Analysis to Profile Histone Post-translational Modification Patterns in Patient Samples
Source: Mol Cell Proteomics. 2015 Oct 13;15(3):866–77. doi: 10.1074/mcp.M115.054510 (PMC4813706; doi:10.1074/mcp.M115.054510)
Supplement: Supplemental Data [file 10.1074_M115.054510_mcp.M115.054510-6.pdf]

H3 K4me

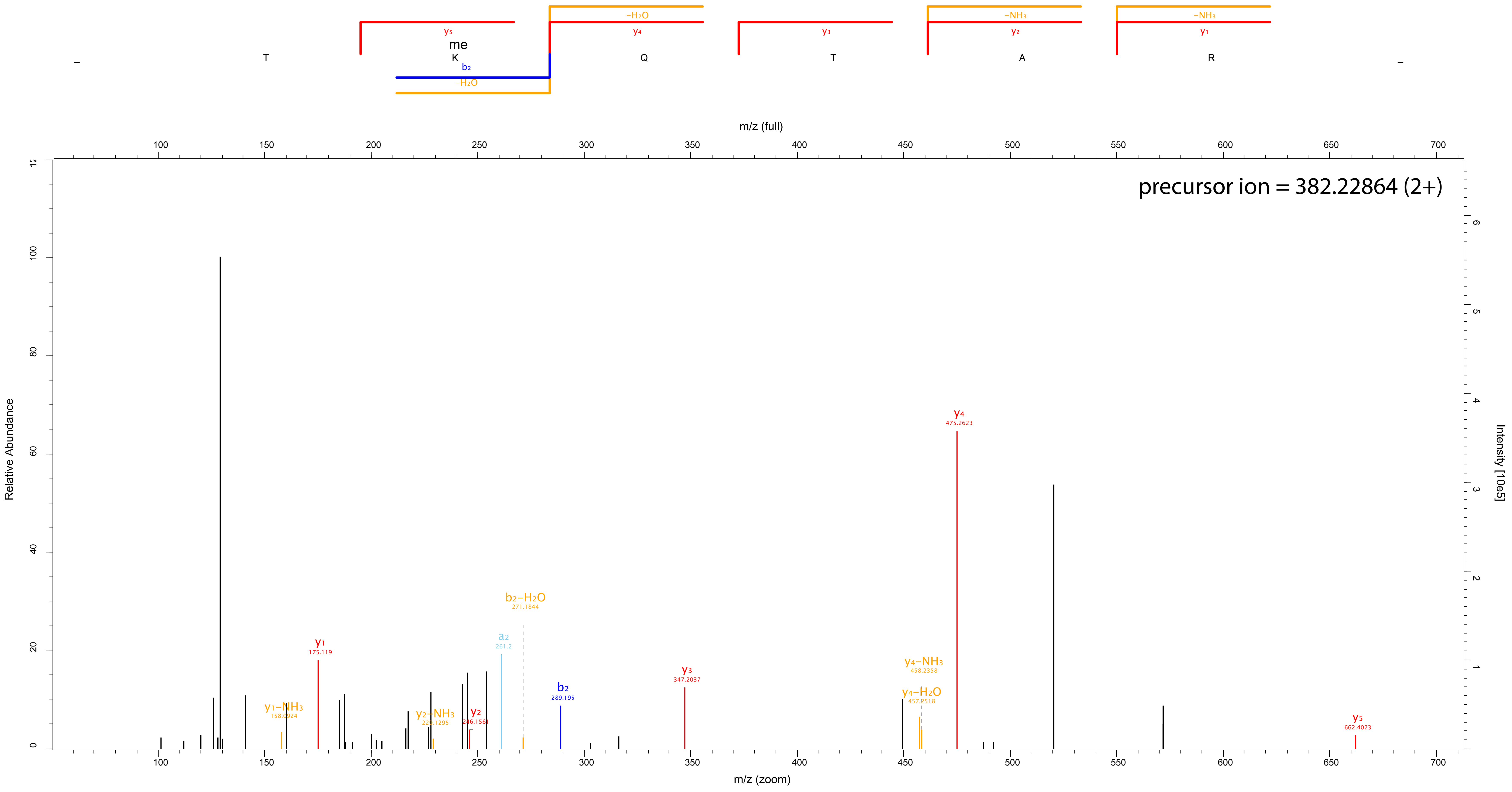

T
me
K
Q
T
A
R
b2
y5
y4
y3
y2
y1
−H2O
−NH3
m/z (full)
precursor ion = 382.22864 (2+)
Relative Abundance
Intensity [10e5]
m/z (zoom)
y1
175.119
y1−NH3
158.0924
y2−NH3
y2
a2
261.2
b2−H2O
271.1844
b2
289.195
y3
347.2037
y4−NH3
458.2358
y4−H2O
y4
475.2623
y5
662.4023

H3 K4me2

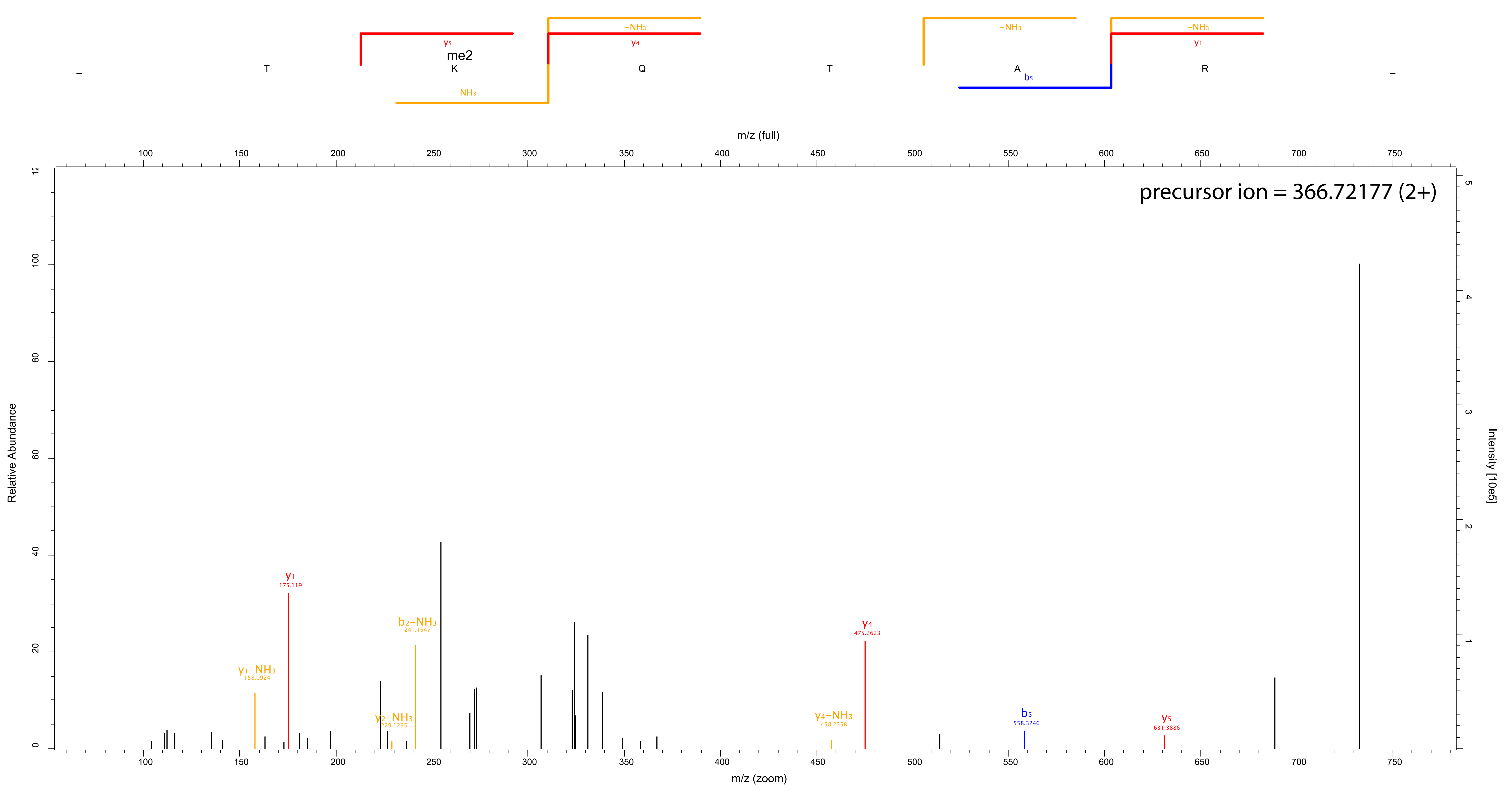

T
me2
K
Q
T
A
R
y5
y4
y1
b5
−NH3
m/z (full)
precursor ion = 366.72177 (2+)
Relative Abundance
Intensity [10e5]
y1
175.119
y1−NH3
158.0924
b2−NH3
241.1547
y2−NH3
229.1295
y4
475.2623
y4−NH3
458.2358
b5
558.3246
y5
631.3886
m/z (zoom)

H3 K4 ac

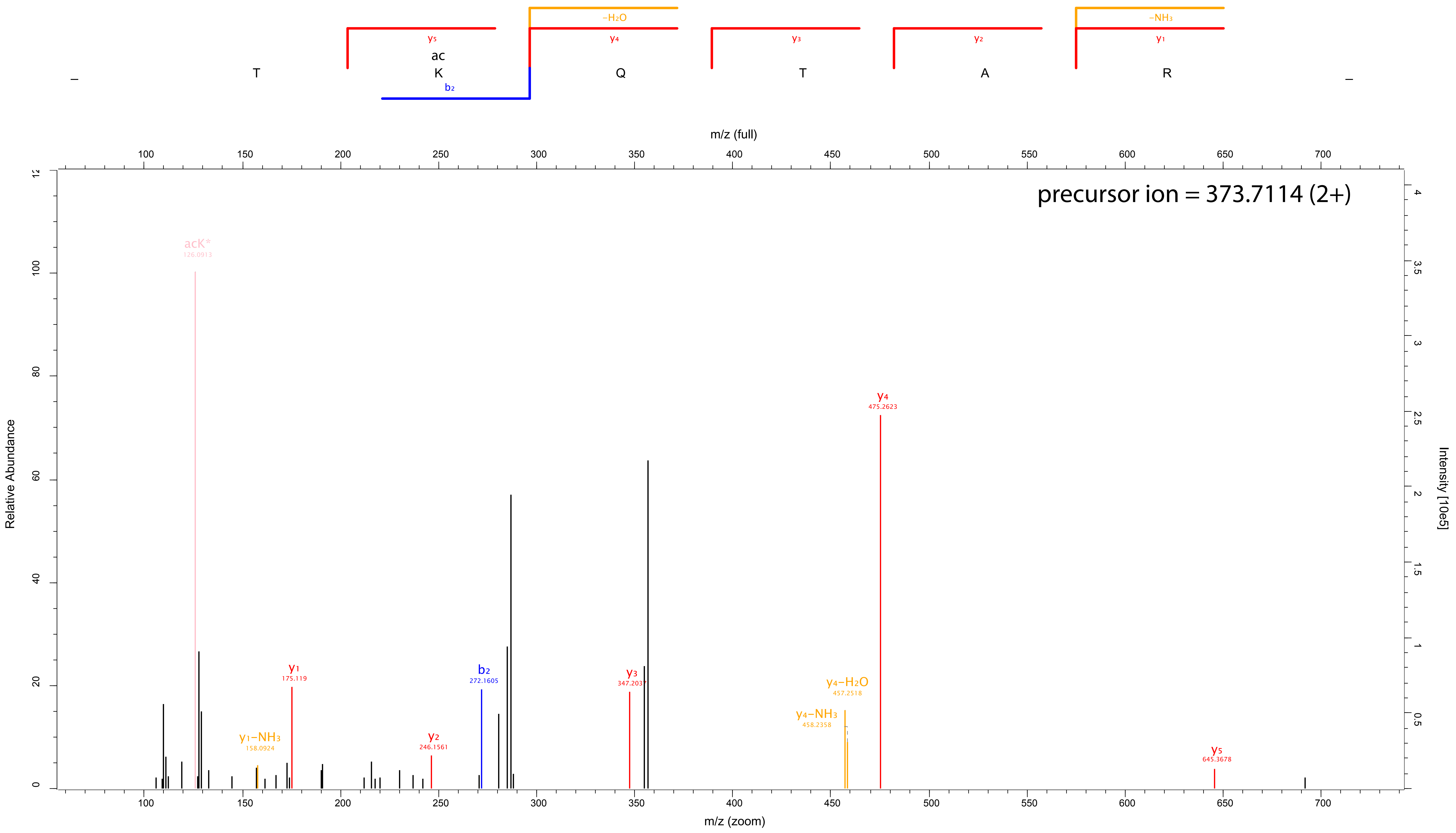

y5
ac
K
b2
-H2O
y4
Q
y3
T
y2
A
-NH3
y1
R
T
m/z (full)
precursor ion = 373.7114 (2+)
acK*
126.0913
y1
175.119
y1-NH3
158.0924
y2
246.1561
b2
272.1605
y3
347.2037
y4-H2O
457.2518
y4-NH3
458.2358
y4
475.2623
y5
645.3678
Relative Abundance
Intensity [10e5]
m/z (zoom)

H3 K9me1

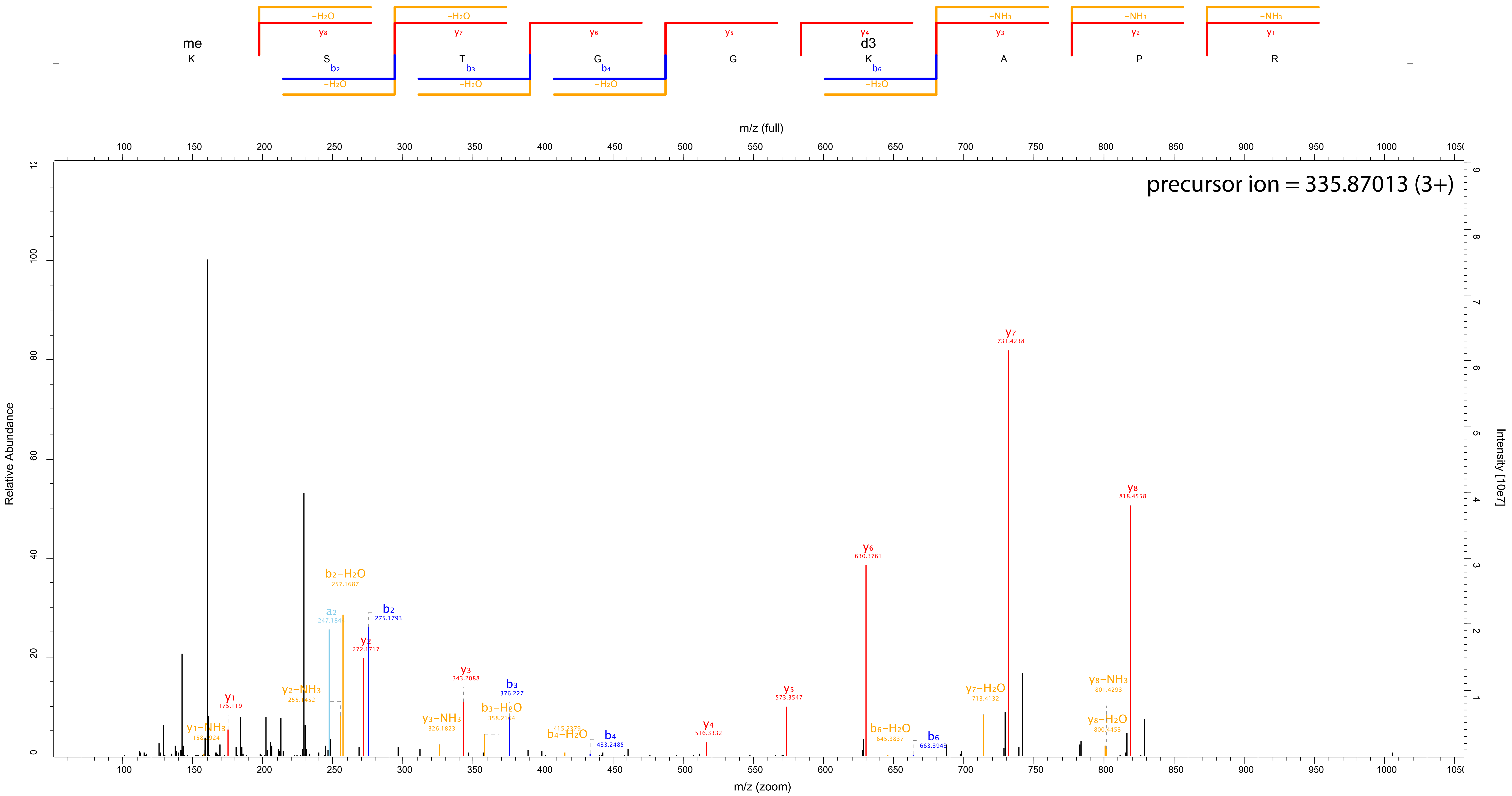

me
K
S
T
G
G
d3
K
A
P
R
y8
y7
y6
y5
y4
y3
y2
y1
-H2O
-NH3
b2
b3
b4
b6
m/z (full)
precursor ion = 335.87013 (3+)
Relative Abundance
Intensity [10e7]
m/z (zoom)
y7
731.4238
y8
818.4558
y6
630.3761
b2-H2O
257.1687
a2
b2
275.1793
y2
272.1717
y1
175.119
y1-NH3
y2-NH3
y3
343.2088
b3
376.227
y3-NH3
326.1823
b3-H2O
b4-H2O
b4
433.2485
y4
516.3332
y5
573.3547
b6-H2O
645.3837
b6
y7-H2O
713.4132
y8-NH3
801.4293
y8-H2O

H3 K14me1

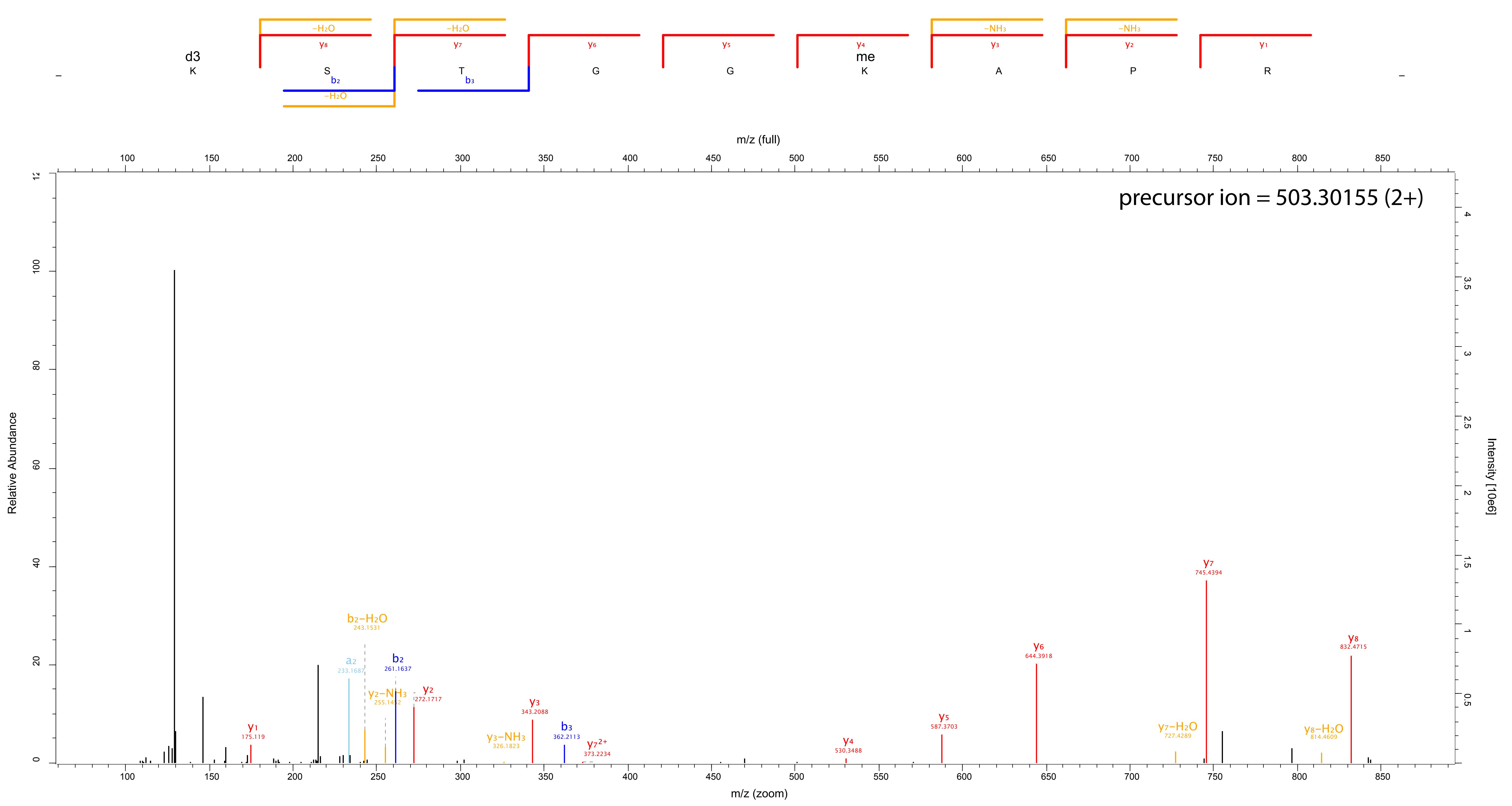

d3
K
S
T
G
G
me
K
A
P
R
precursor ion = 503.30155 (2+)
m/z (full)
m/z (zoom)
Relative Abundance
Intensity [10e6]
b2-H2O 243.1531
a2 233.1687
b2 261.1637
y2-NH3 255.1452
y2 272.1717
y1 175.119
y3 343.2088
y3-NH3 326.1823
b3 362.2113
y7 2+ 373.2234
y4 530.3488
y5 587.3703
y6 644.3918
y7-H2O 727.4289
y7 745.4394
y8-H2O 814.4609
y8 832.4715

H3 K9me2

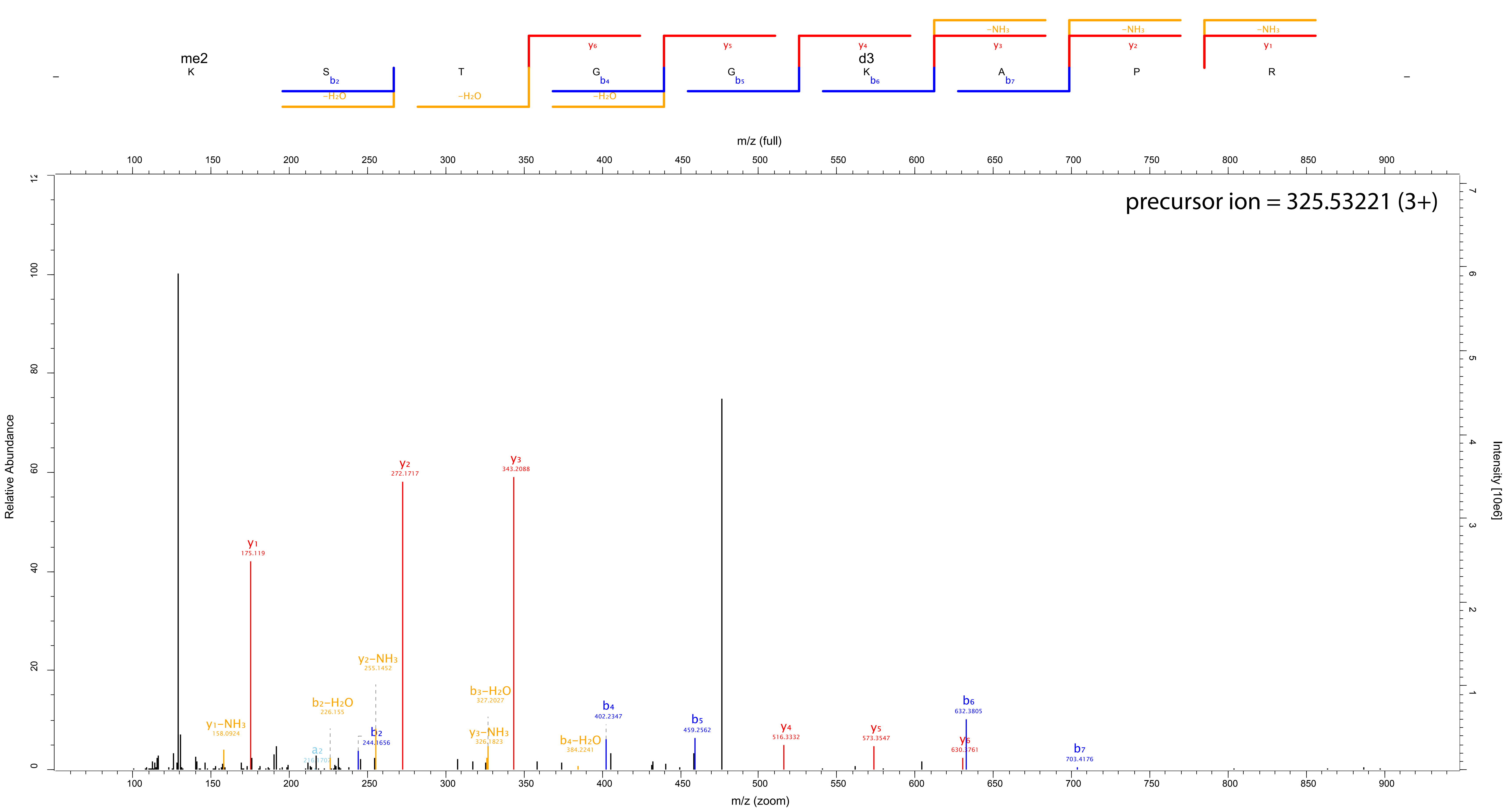

me2
K
S
T
G
G
d3
K
A
P
R
b2
b4
b5
b6
b7
y6
y5
y4
y3
y2
y1
-H2O
-NH3
m/z (full)
precursor ion = 325.53221 (3+)
y1 175.119
y2 272.1717
y3 343.2088
y1-NH3 158.0924
b2-H2O 226.155
y2-NH3 255.1452
b2 244.1656
b3-H2O 327.2027
y3-NH3 326.1823
b4-H2O 384.2241
b4 402.2347
b5 459.2562
y4 516.3332
y5 573.3547
y6 630.3761
b6 632.3805
b7 703.4176
Relative Abundance
Intensity [10e6]
m/z (zoom)

H3 K9me3

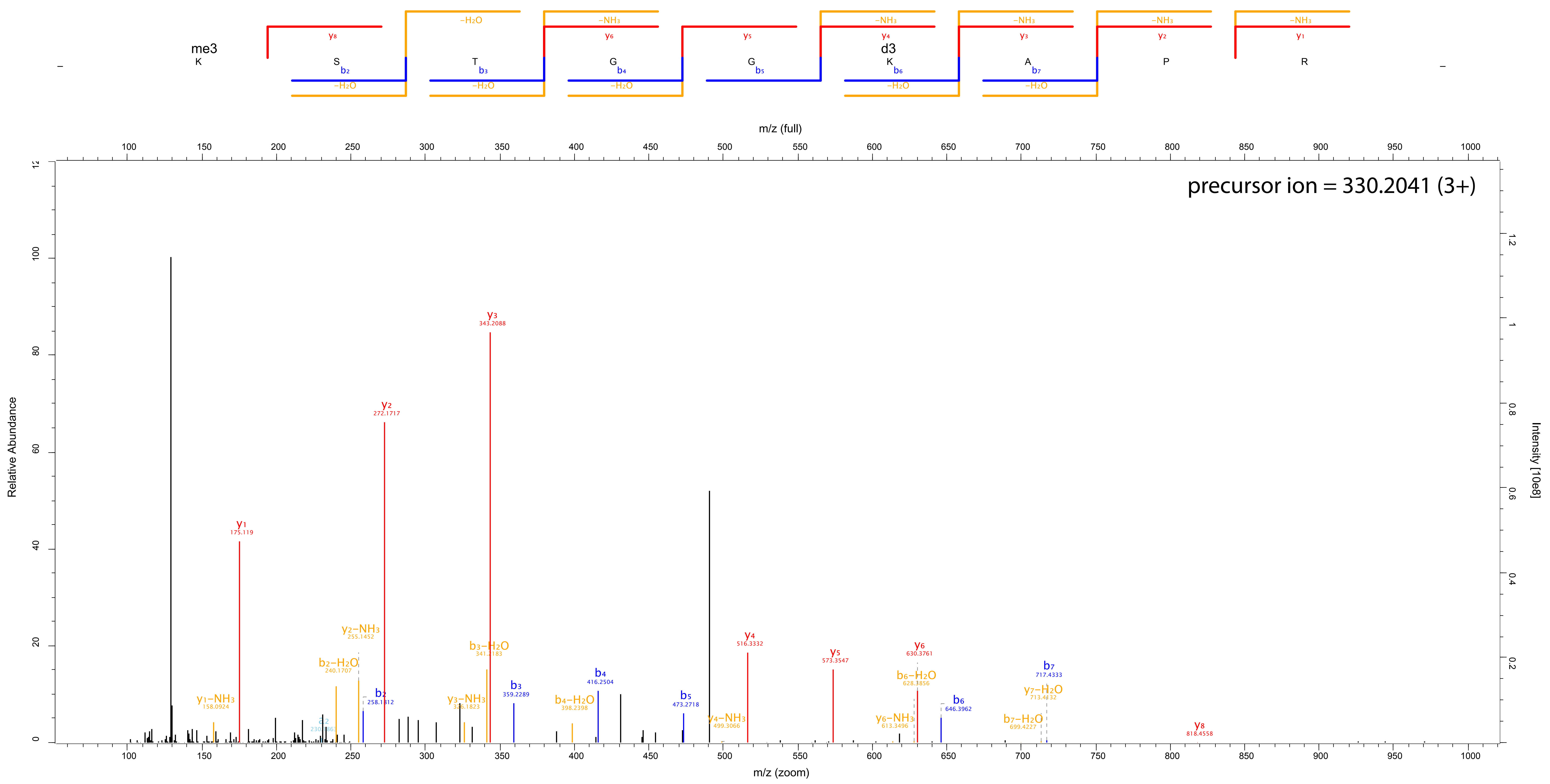

me3
K
S
T
G
G
d3
K
A
P
R
precursor ion = 330.2041 (3+)
m/z (full)
m/z (zoom)
Relative Abundance
Intensity [10e8]
y1 175.119
y2 272.1717
y3 343.2088
y4 516.3332
y5 573.3547
y6 630.3761
y8 818.4558
b3 359.2289
b4 416.2504
b5 473.2718
b6 646.3962
b7 717.4333
y1−NH3 158.0924
b2−H2O 240.1707
y2−NH3 255.1452
b3−H2O 341.2183
b4−H2O 398.2398
y4−NH3 499.3066
y6−NH3 613.3496
b6−H2O 628.3856
b7−H2O 699.4227

H3 K9ac

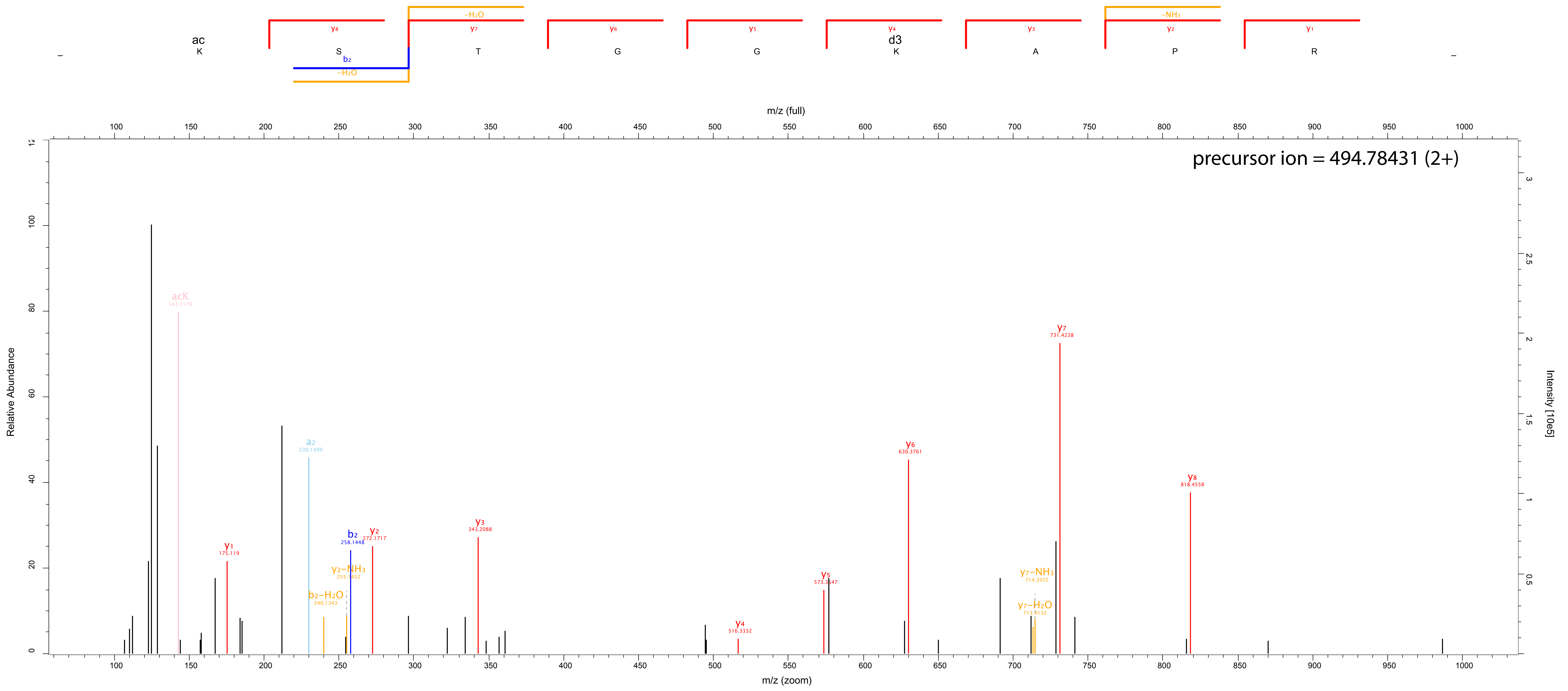
ac
d3
m/z (full)
precursor ion = 494.78431 (2+)
Relative Abundance
Intensity [10e5]
m/z (zoom)
acK
143.1179
y1
175.119
a2
230.1499
b2-H2O
240.1343
y2-NH3
255.1452
b2
258.1448
y2
272.1717
y3
343.2088
y4
516.3332
y5
573.3547
y6
630.3761
y7-NH3
714.3972
y7-H2O
713.4132
y7
731.4238
y8
818.4558

H3 K14ac

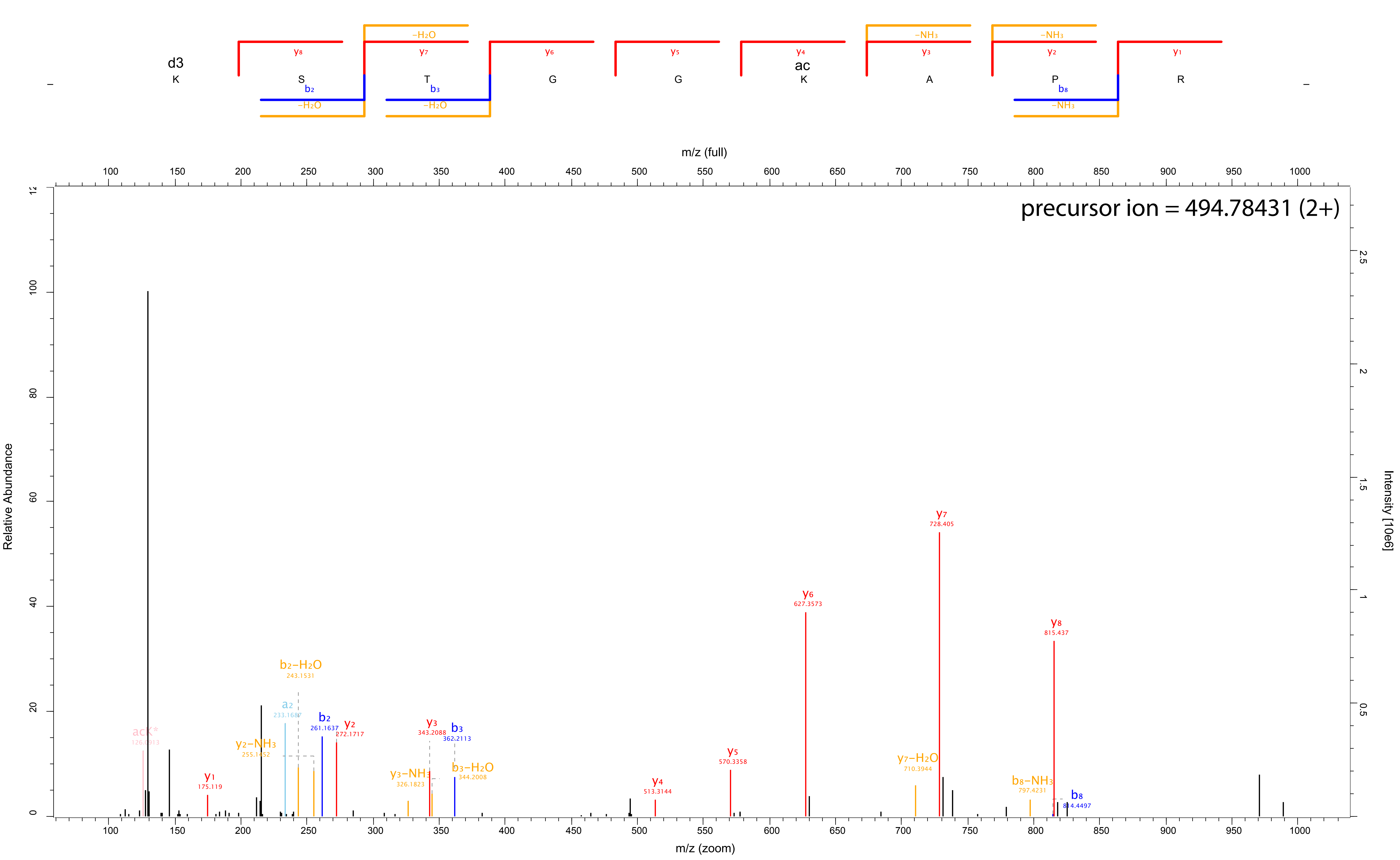

d3 K S T G G ac K A P R
y8 y7 y6 y5 y4 y3 y2 y1
b2 b3 b8
-H2O
-NH3
m/z (full)
precursor ion = 494.78431 (2+)
Relative Abundance
Intensity [10e6]
m/z (zoom)
y7 728.405
y6 627.3573
y8 815.437
b2-H2O 243.1531
a2 233.1687
b2 261.1637
y2 272.1717
y3 343.2088
b3 362.2113
y2-NH3 255.1452
y1 175.119
y3-NH3 326.1823
b3-H2O 344.2008
y4 513.3144
y5 570.3358
y7-H2O 710.3944
b8-NH3 797.4231
b8

H3 K9me1K14ac

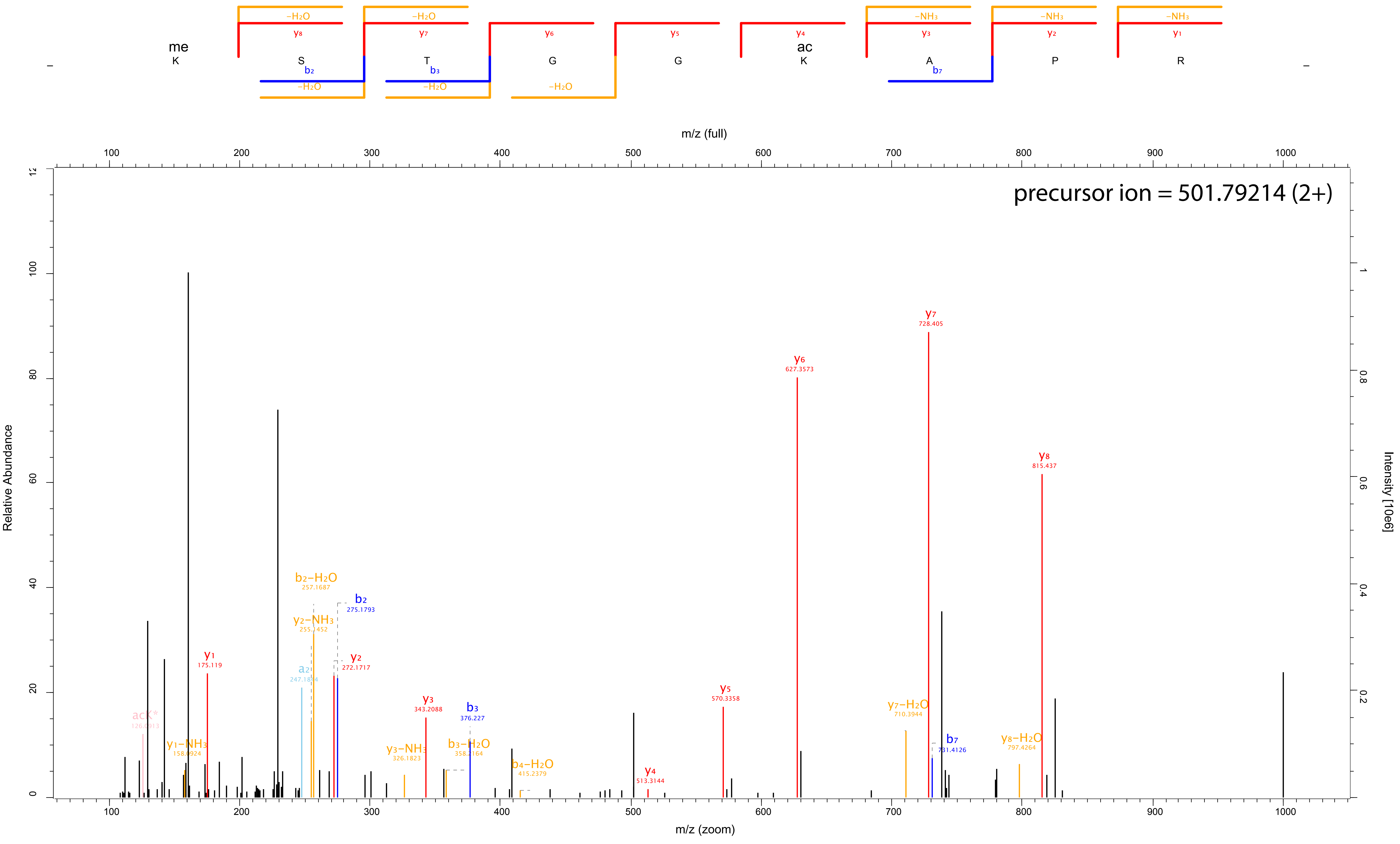

H3 K9me2K14ac

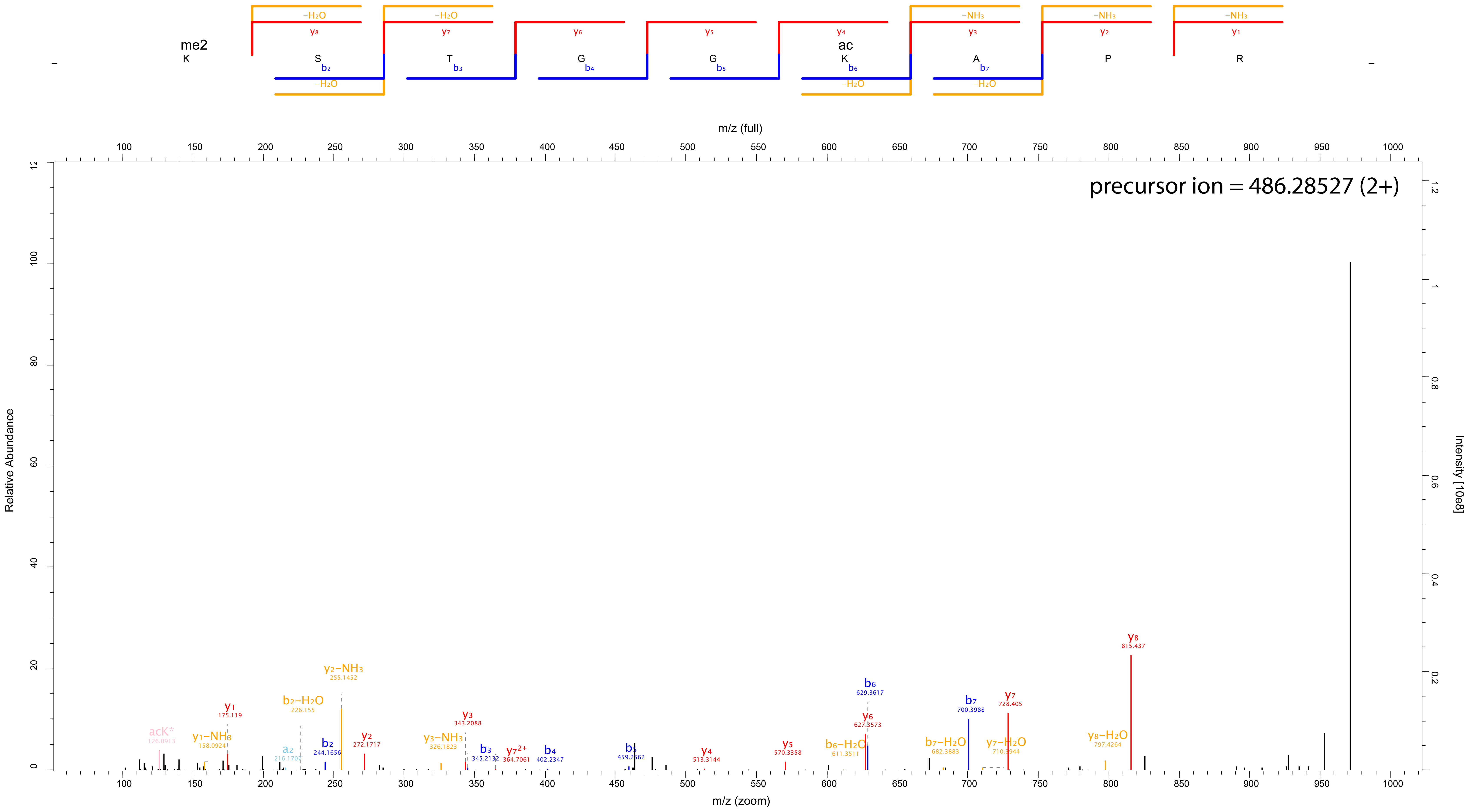

H3 K9me3K14ac

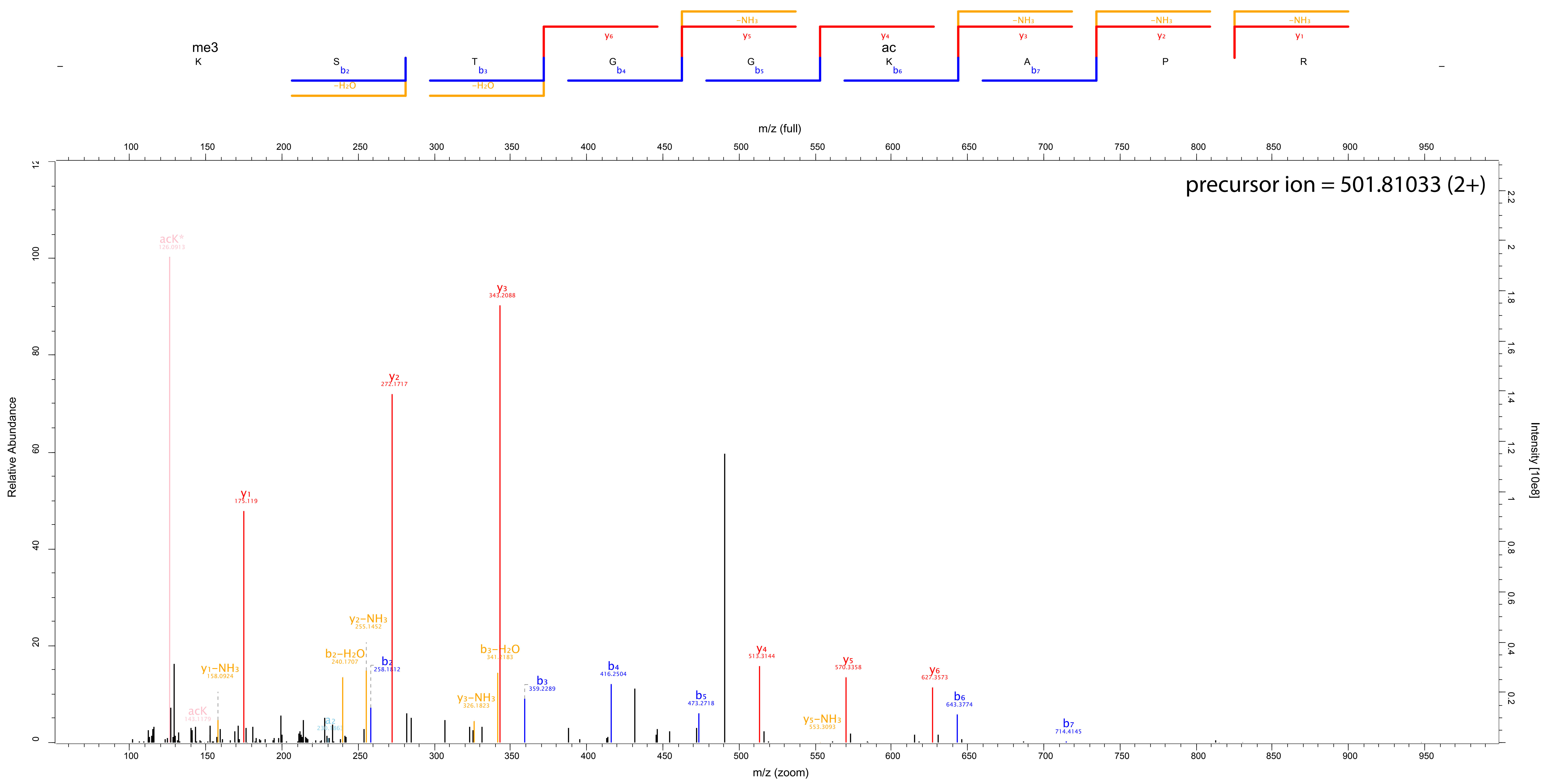

H3 K9acK14ac

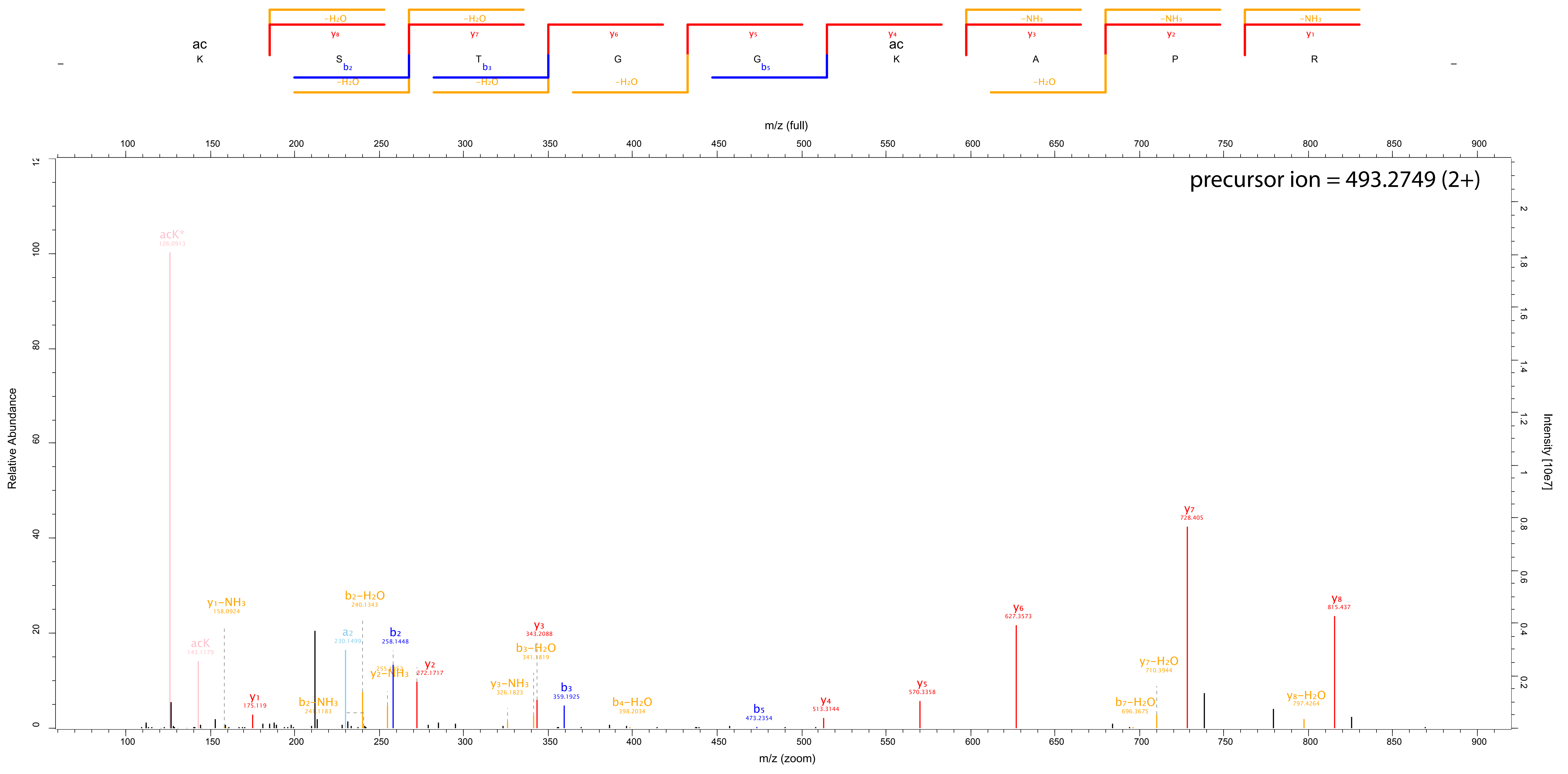

H3 K9me1K14me1

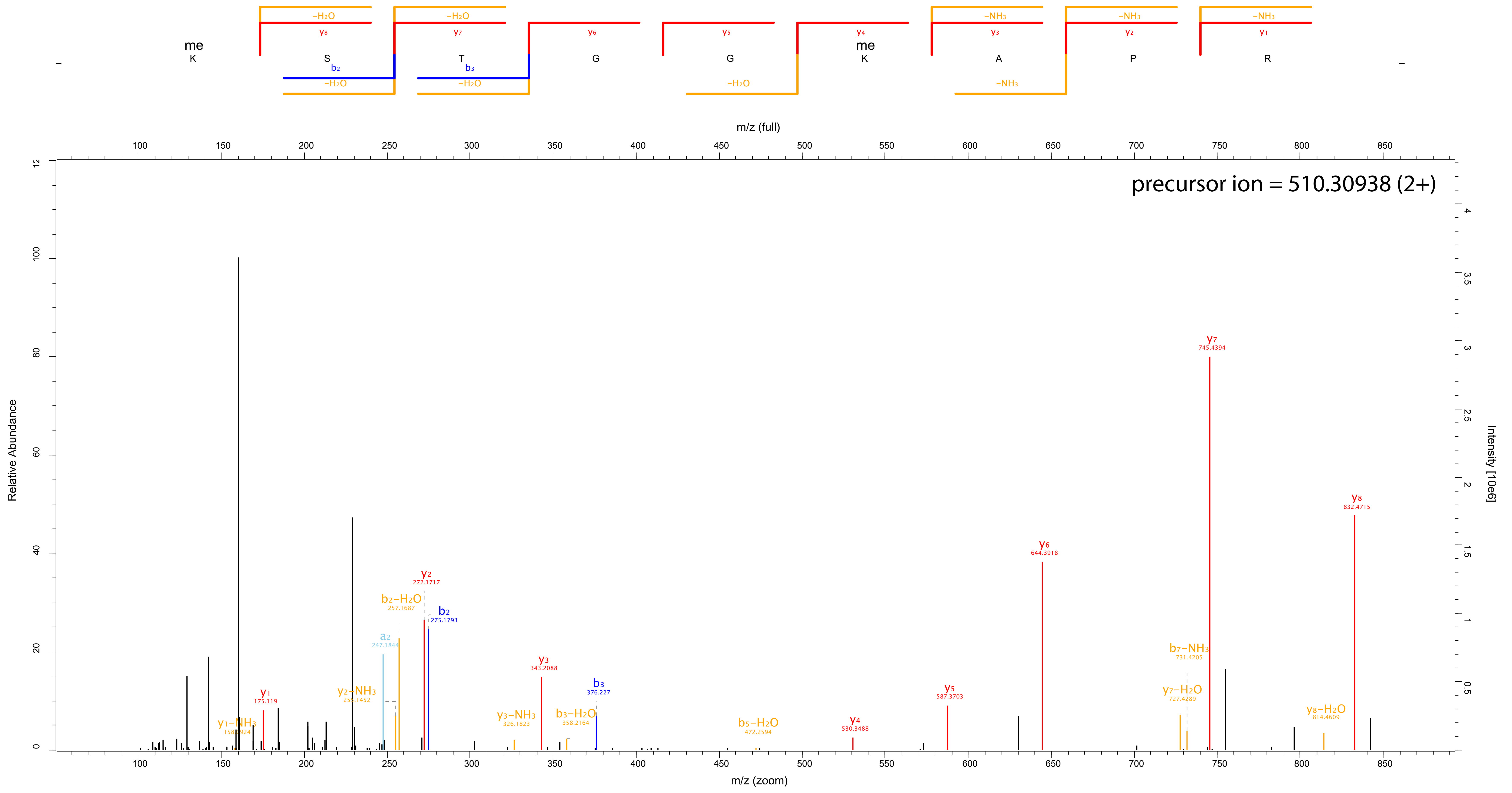

H3 K9me2K14me1

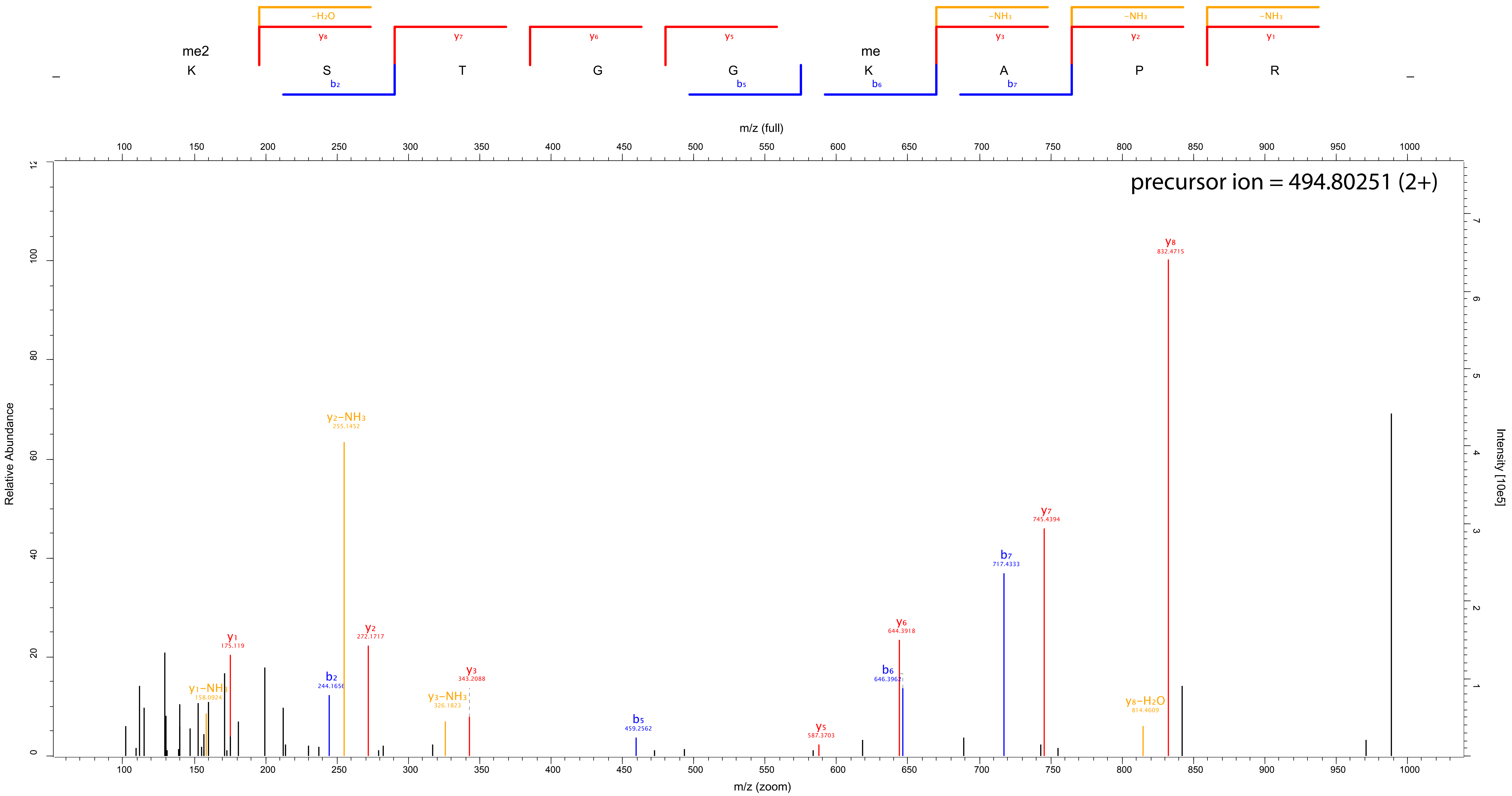

H3 K9me3K14me1

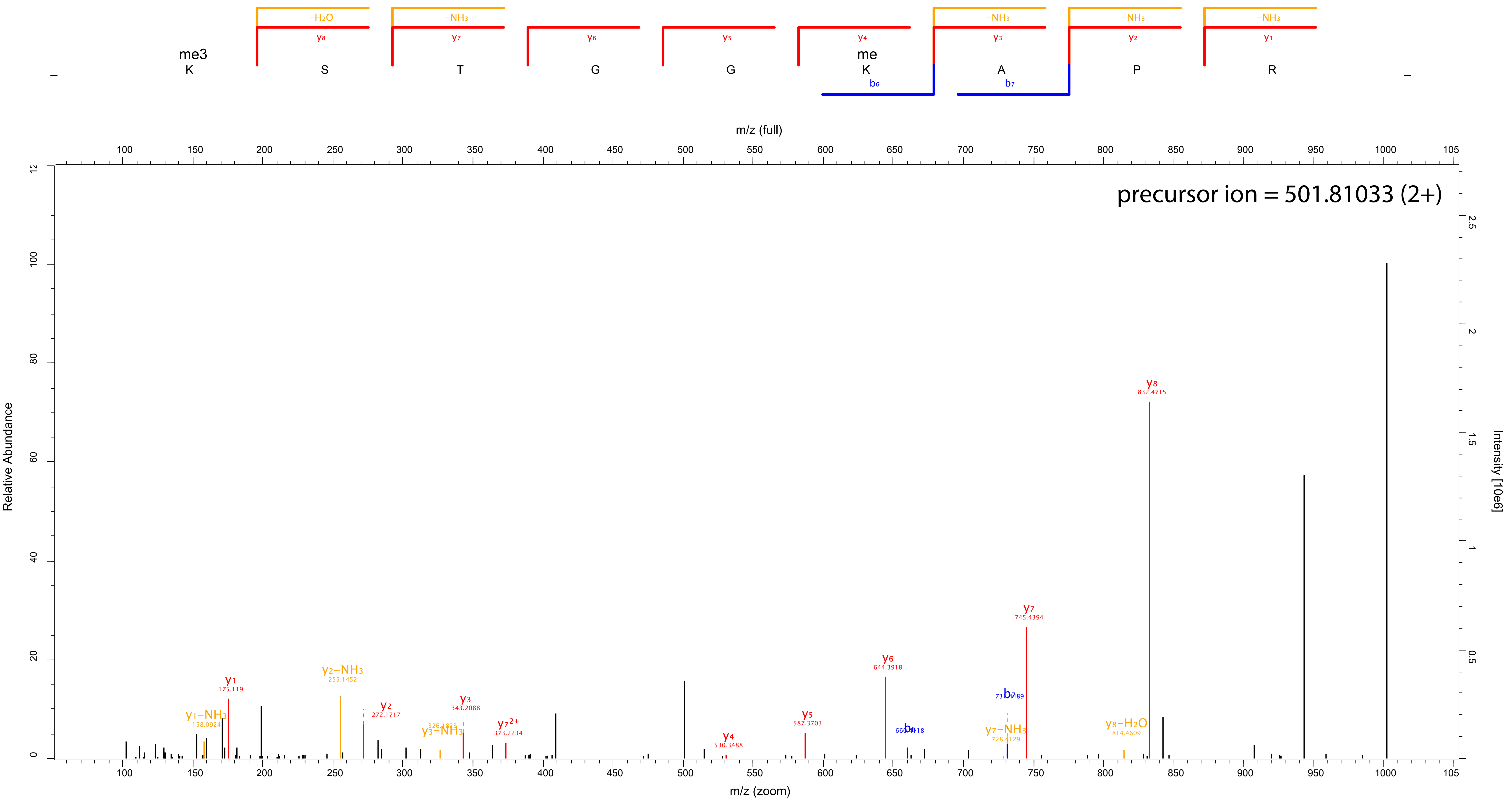

H3 K9form

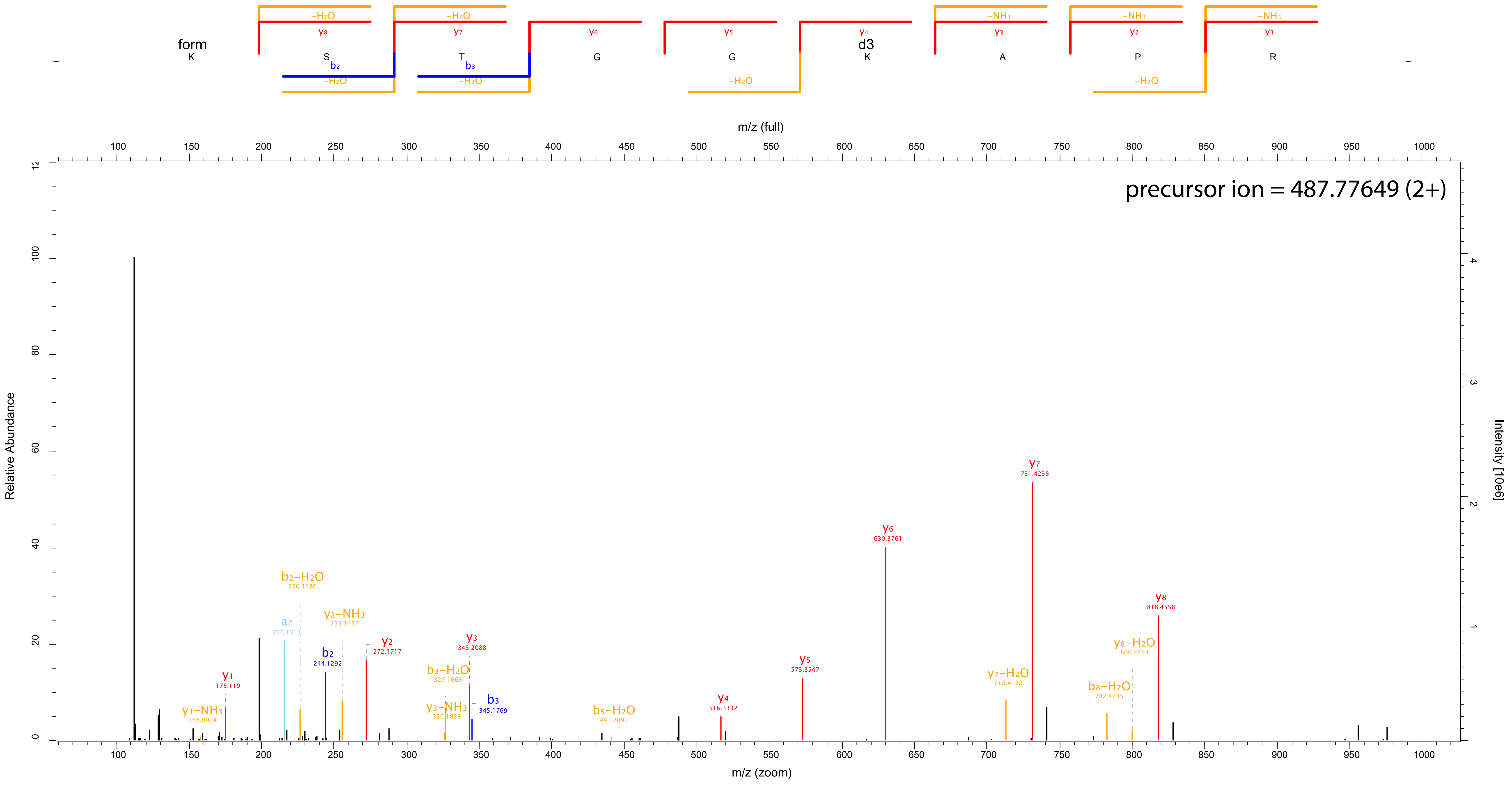

form K
S
T
G
G
d3 K
A
P
R
y8
y7
y6
y5
y4
y3
y2
y1
b2
b3
-H2O
-NH3
m/z (full)
precursor ion = 487.77649 (2+)
Relative Abundance
Intensity [10e6]
y1 175.119
y1-NH3 158.0924
a2 216.1342
b2-H2O 226.1186
b2 244.1292
y2-NH3 255.1452
y2 272.1717
y3 343.2088
b3-H2O 327.1663
y3-NH3 326.1823
b3 345.1769
b5-H2O 441.2092
y4 516.3332
y5 573.3547
y6 630.3761
y7-H2O 713.4132
y7 731.4238
b8-H2O 782.4235
y8-H2O 800.4453
y8 818.4558
m/z (zoom)

H3 K23ac

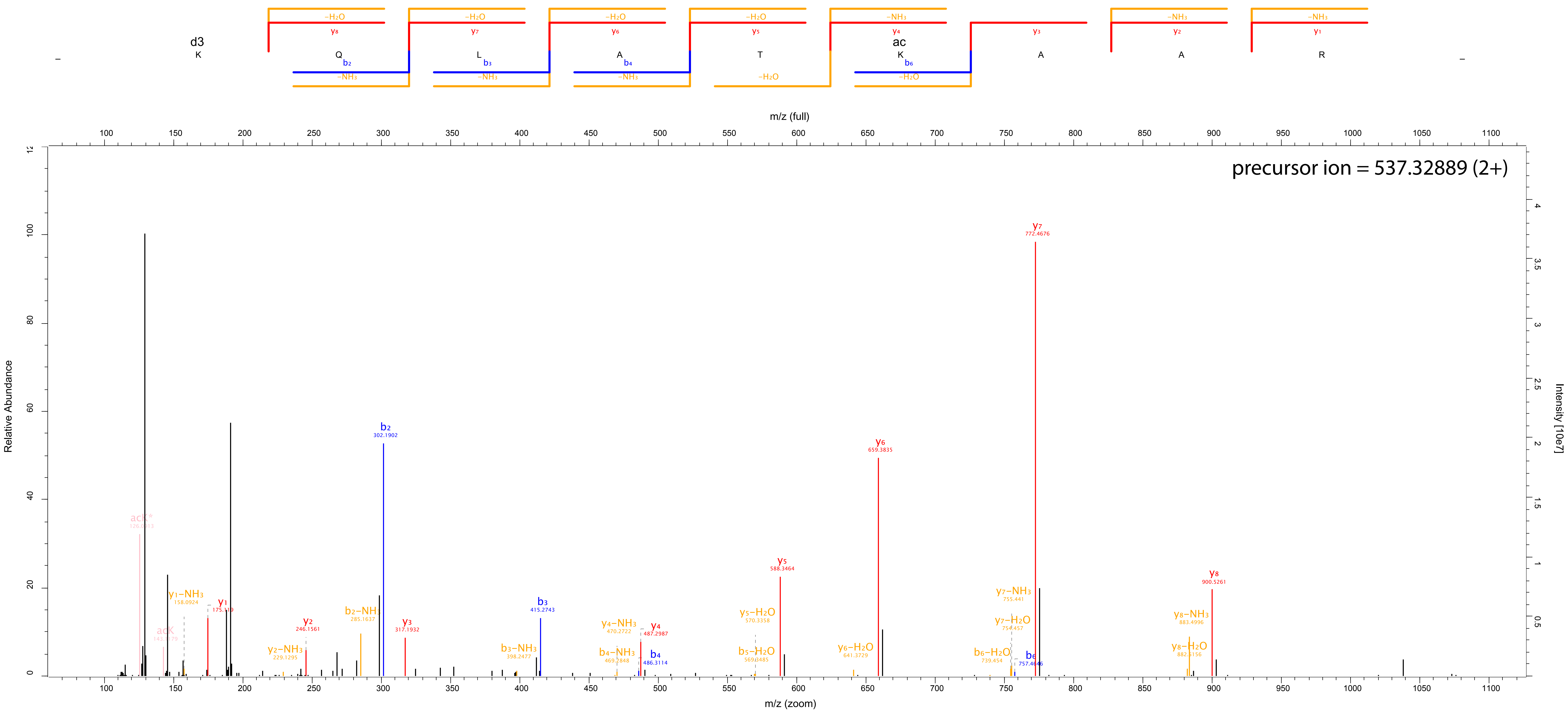

d3
K
Q
L
A
T
ac
K
A
A
R
precursor ion = 537.32889 (2+)
m/z (full)
m/z (zoom)
Relative Abundance
Intensity [10e7]
y7
772.4676
b2
302.1902
y6
659.3835
y5
588.3464
y8
900.5261
b3
415.2743

H3 K18acK23ac

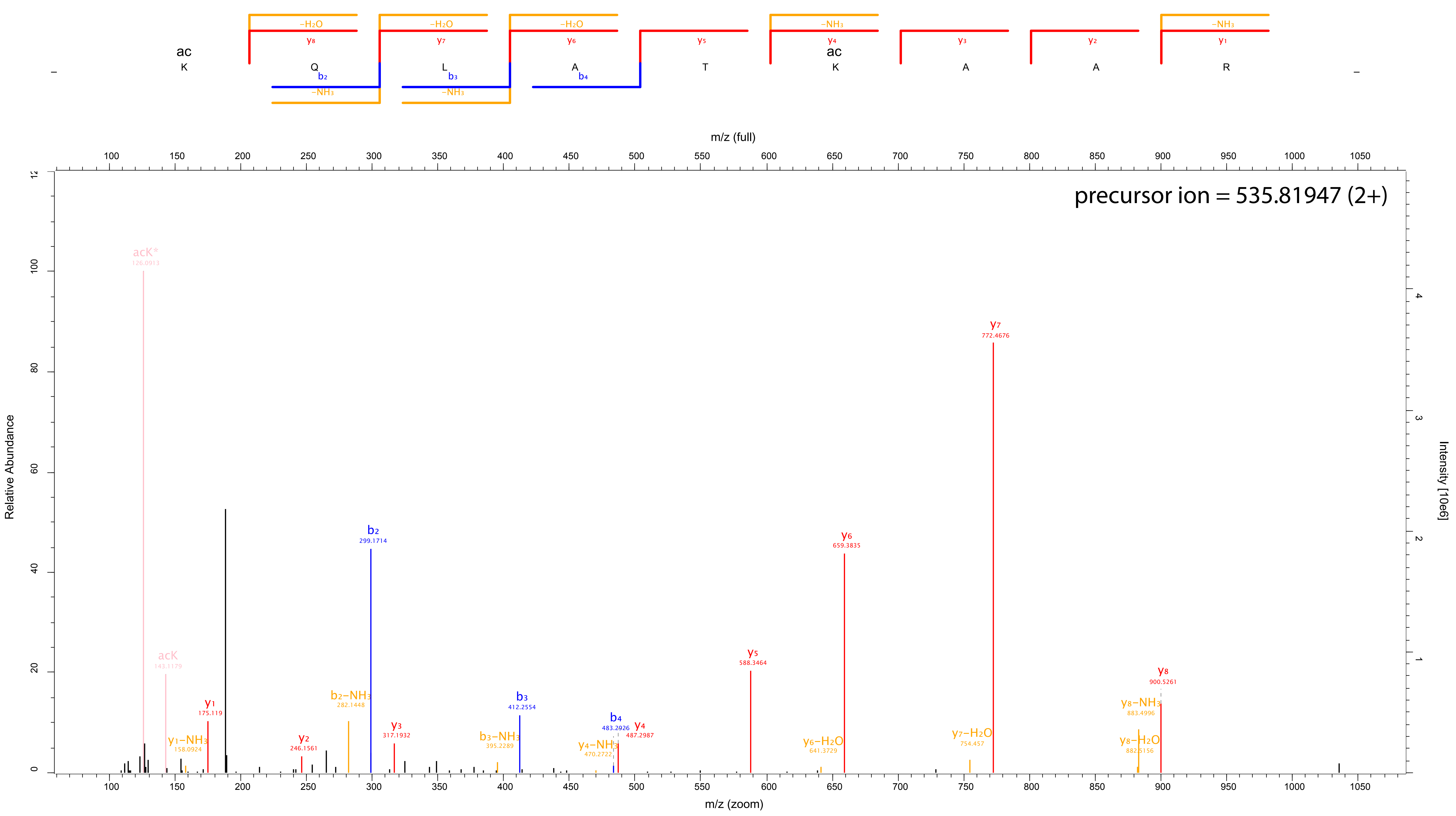

H3 K18me1

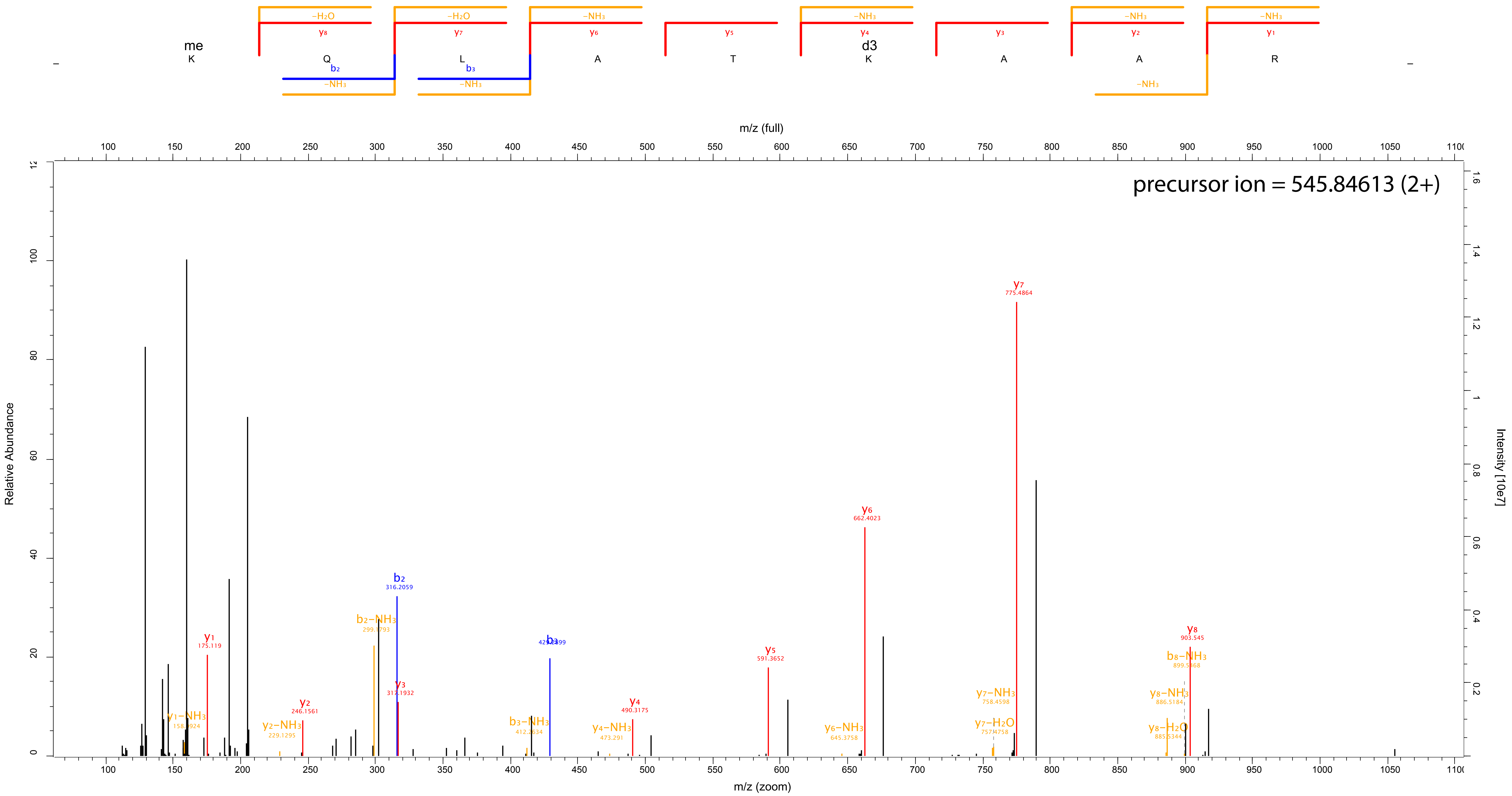
me
K
Q
L
A
T
d3
K
A
A
R
m/z (full)
precursor ion = 545.84613 (2+)
Relative Abundance
Intensity [10e7]
m/z (zoom)
y7
775.4864
y6
662.4023
y5
591.3652
y4
490.3175
y2
246.1561
y1
175.119
b2
316.2059
b2−NH3
y3
y8
903.545
b8−NH3
y7−NH3
758.4598
y7−H2O
y8−NH3
886.5184
y6−NH3
645.3758
y4−NH3
473.291
y2−NH3
229.1295
b3−NH3
y8−H2O

H3 K23me1

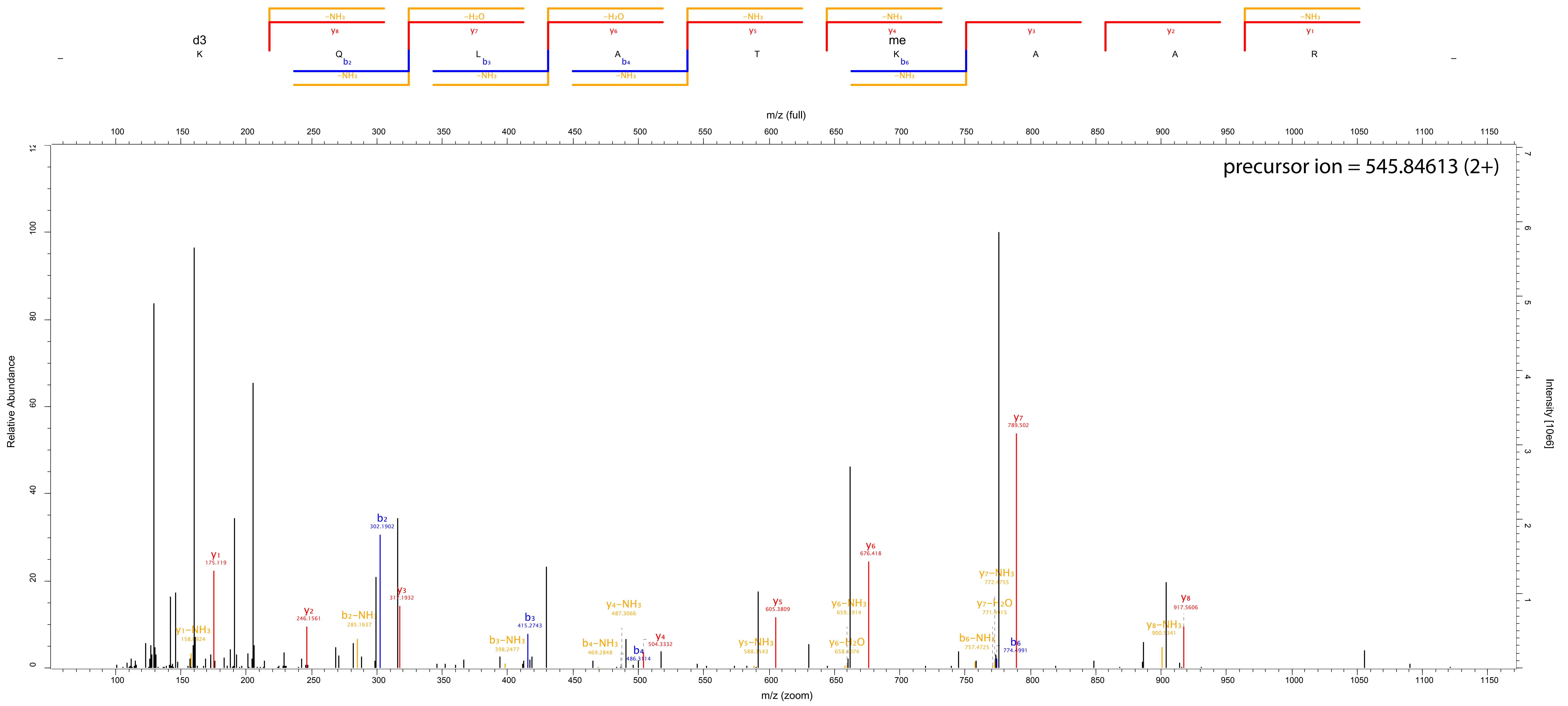

d3 K Q L A T me K A A R
precursor ion = 545.84613 (2+)
m/z (full)
m/z (zoom)
Relative Abundance
Intensity [10e6]
y1 175.119
y2 246.1561
b2 302.1902
y3 317.1932
b3 415.2743
y4 504.3332
y5 605.3809
y6 676.418
y7 789.502
y8 917.5606
b6 774.4991
b2-NH3 285.1637
b3-NH3 398.2477
y4-NH3 487.3066
b4-NH3 469.2848
y7-NH3 772.4755
y8-NH3 900.5341

H3 K18me1K23me1

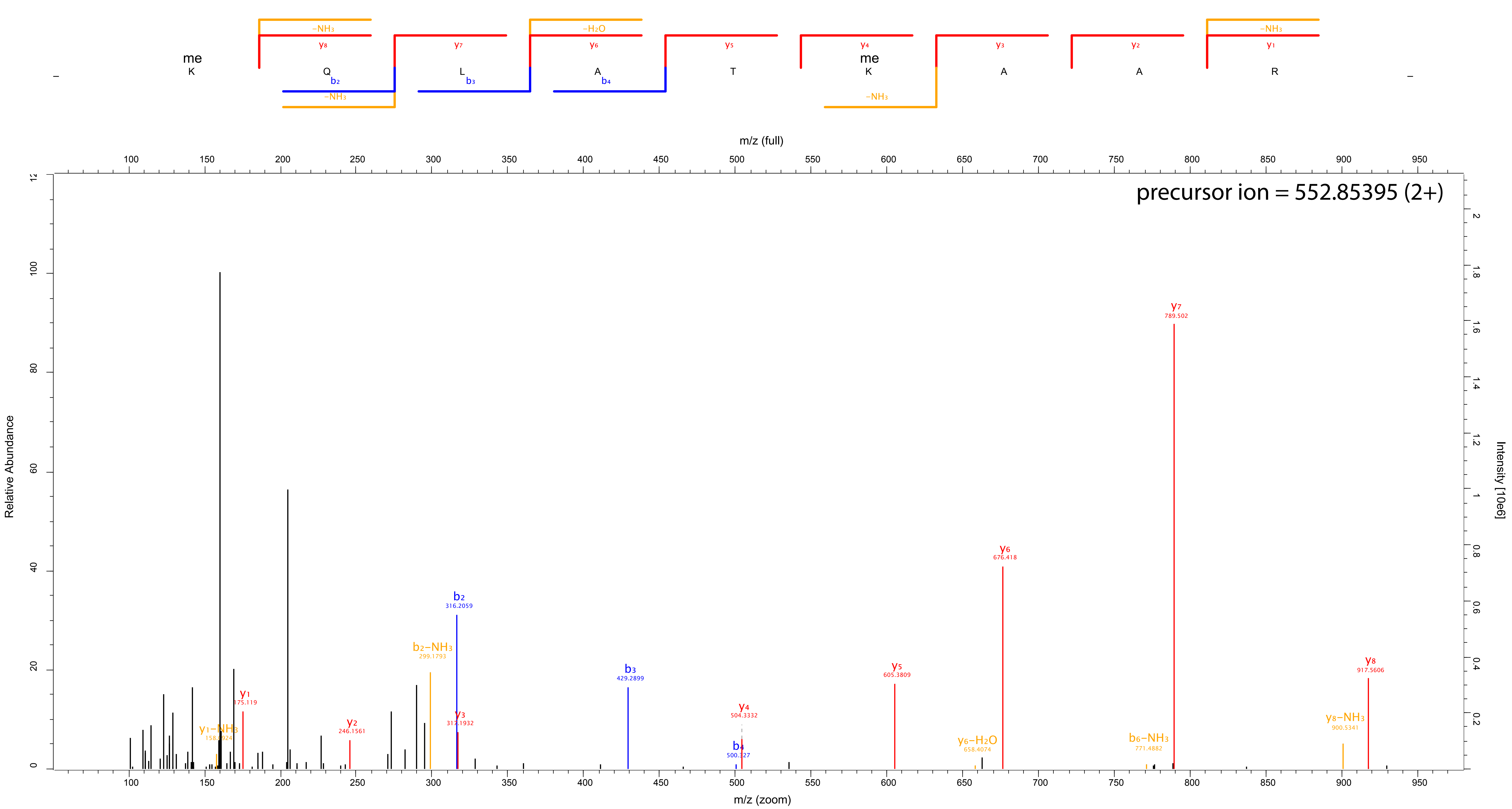

H3 K18form

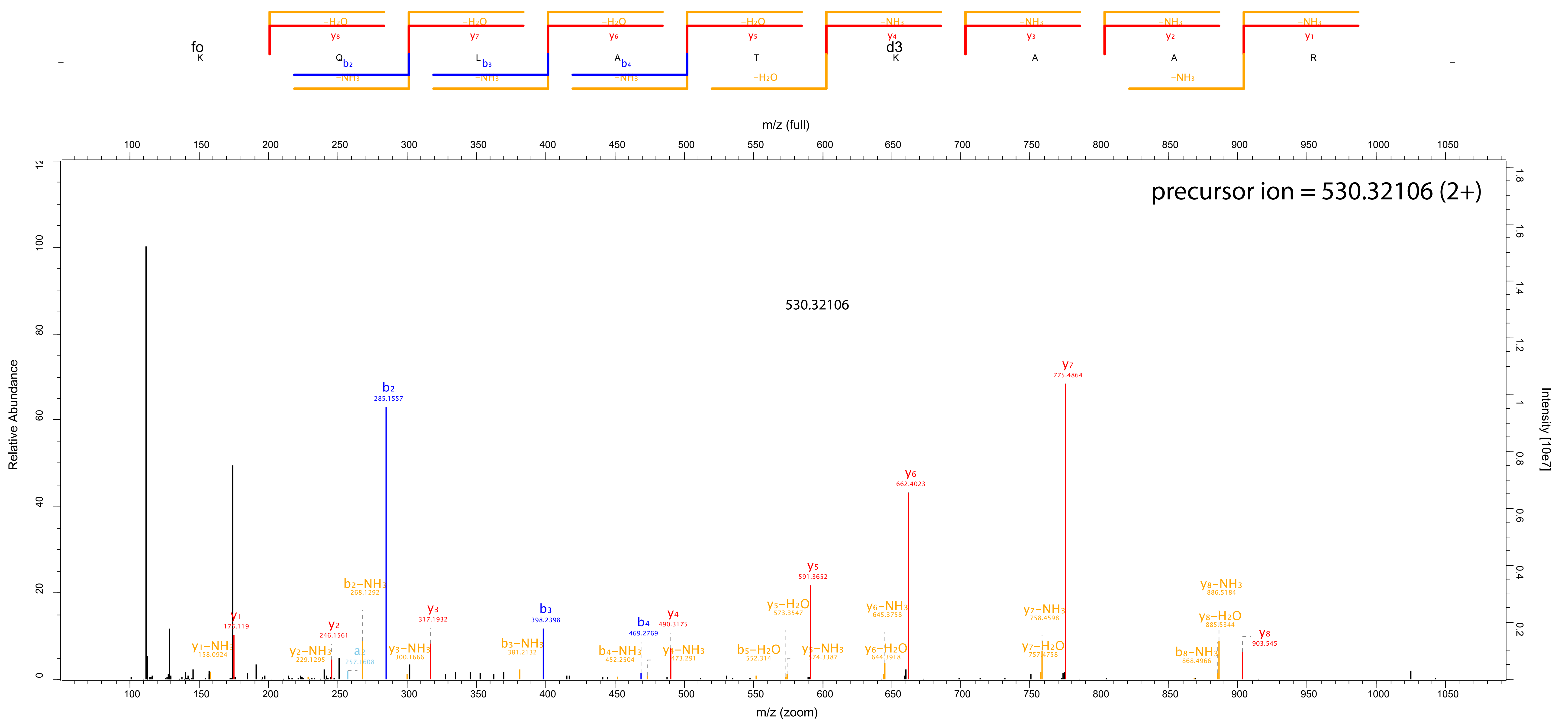

precursor ion = 530.32106 (2+)
530.32106
m/z (full)
m/z (zoom)
Relative Abundance
Intensity [10e7]
y7
775.4864
b2
285.1557
y6
662.4023
y5
591.3652
y4
490.3175
y3
317.1932
b3
398.2398
b4
469.2769
y1
175.119
y2
246.1561
y8
903.545
b2−NH3
268.1292
y8−NH3
886.5184
y8−H2O
885.5344
b8−NH3
868.4966
y7−NH3
758.4598
y7−H2O
757.4758
y6−NH3
645.3758
y6−H2O
644.3918
y5−H2O
573.3547
y5−NH3
574.3387
b5−H2O
552.314
y4−NH3
473.291
b4−NH3
452.2504
b3−NH3
381.2132
y3−NH3
300.1666
y2−NH3
229.1295
y1−NH3
158.0924
a2
257.1608

H3 K23form

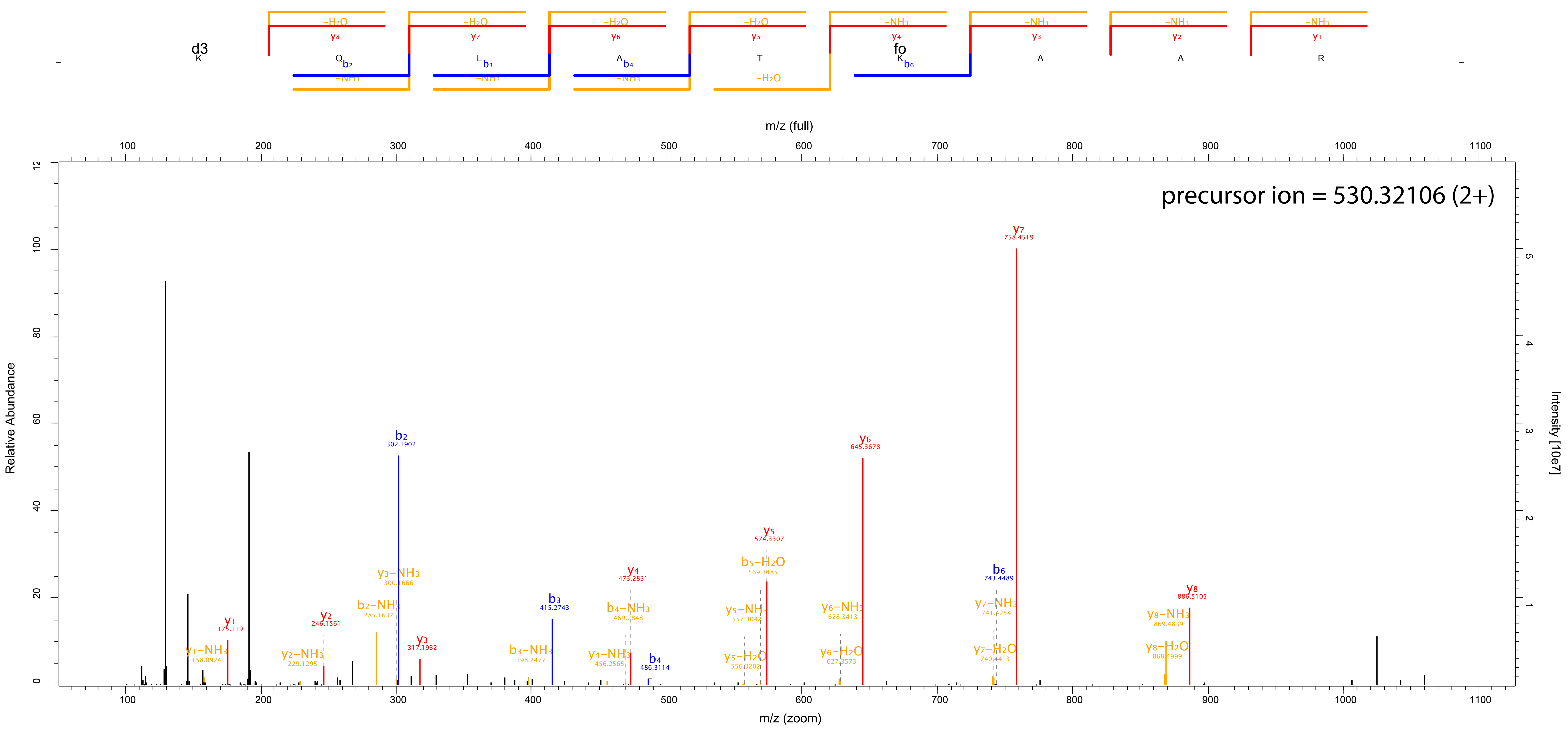

precursor ion = 530.32106 (2+)
m/z (full)
m/z (zoom)
Relative Abundance
Intensity [10e7]
y7 758.4519
y6 645.3678
b2 302.1902
y5 574.3307
b5−H2O 569.3485
b6 743.4489
y4 473.2831
y8 886.5105
b3 415.2743
y1 175.119
y2 246.1561
y3 317.1932
b4 486.3114

H3 K27me1

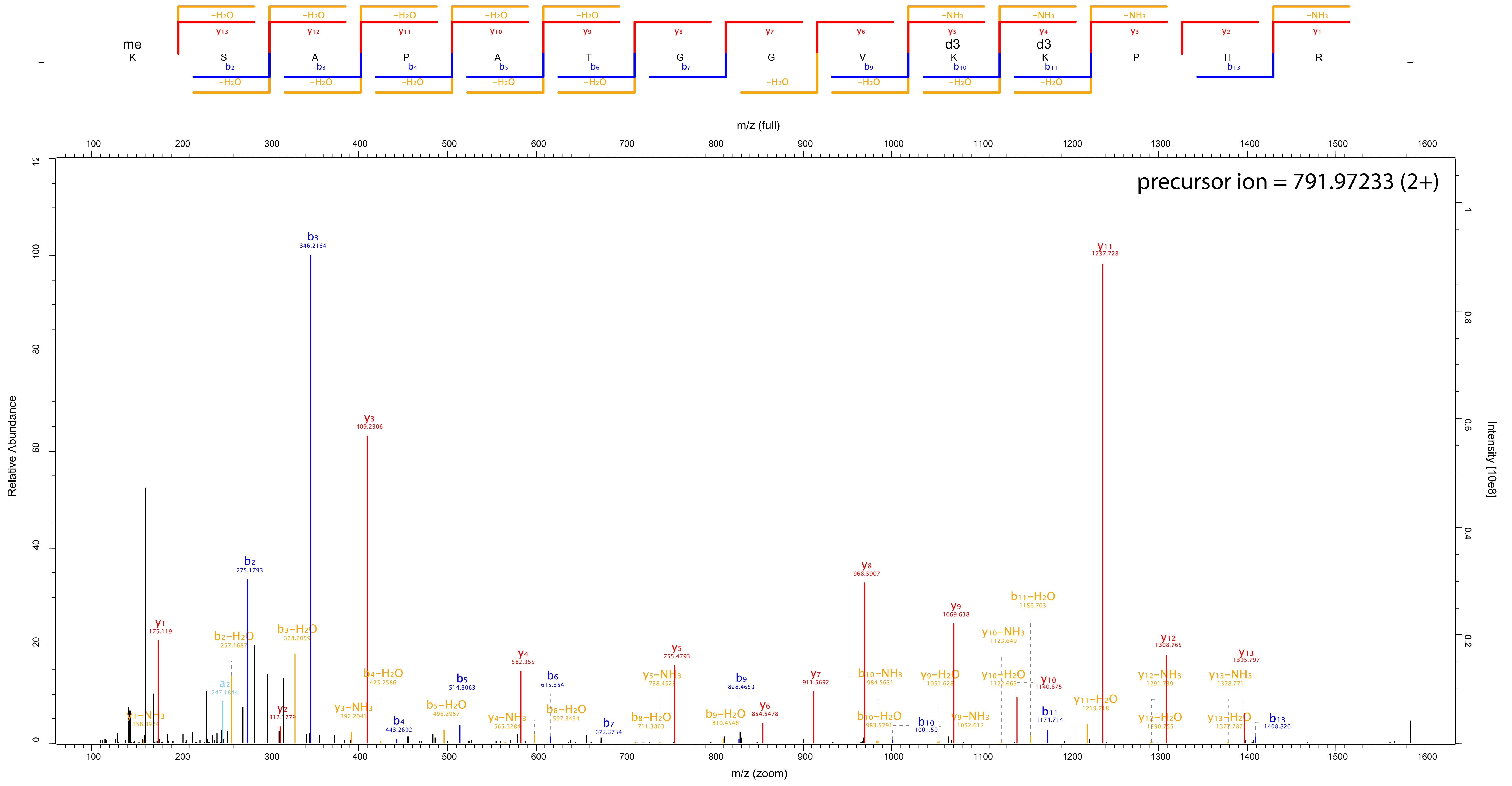

me
K
S
A
P
A
T
G
G
V
d3
K
d3
K
P
H
R
m/z (full)
m/z (zoom)
Relative Abundance
Intensity [10e8]
precursor ion = 791.97233 (2+)
b3
346.2164
y11
1237.728
y3
409.2306
b2
275.1793
y8
968.5907
y9
1069.638
b11−H2O
1156.703
y1
175.119
b2−H2O
257.1687
b3−H2O
328.2059
y4
582.355
y5
755.4793
y12
1308.765
y13
1395.797
b5
514.3063
b6
615.354
b9
828.4653
y7
911.5692
y6
854.5478
y10
1140.675
b4
443.2692
b7
672.3754
b10
1001.59
b11
1174.714
b13
1408.826
a2
247.1844

H3 K36me1

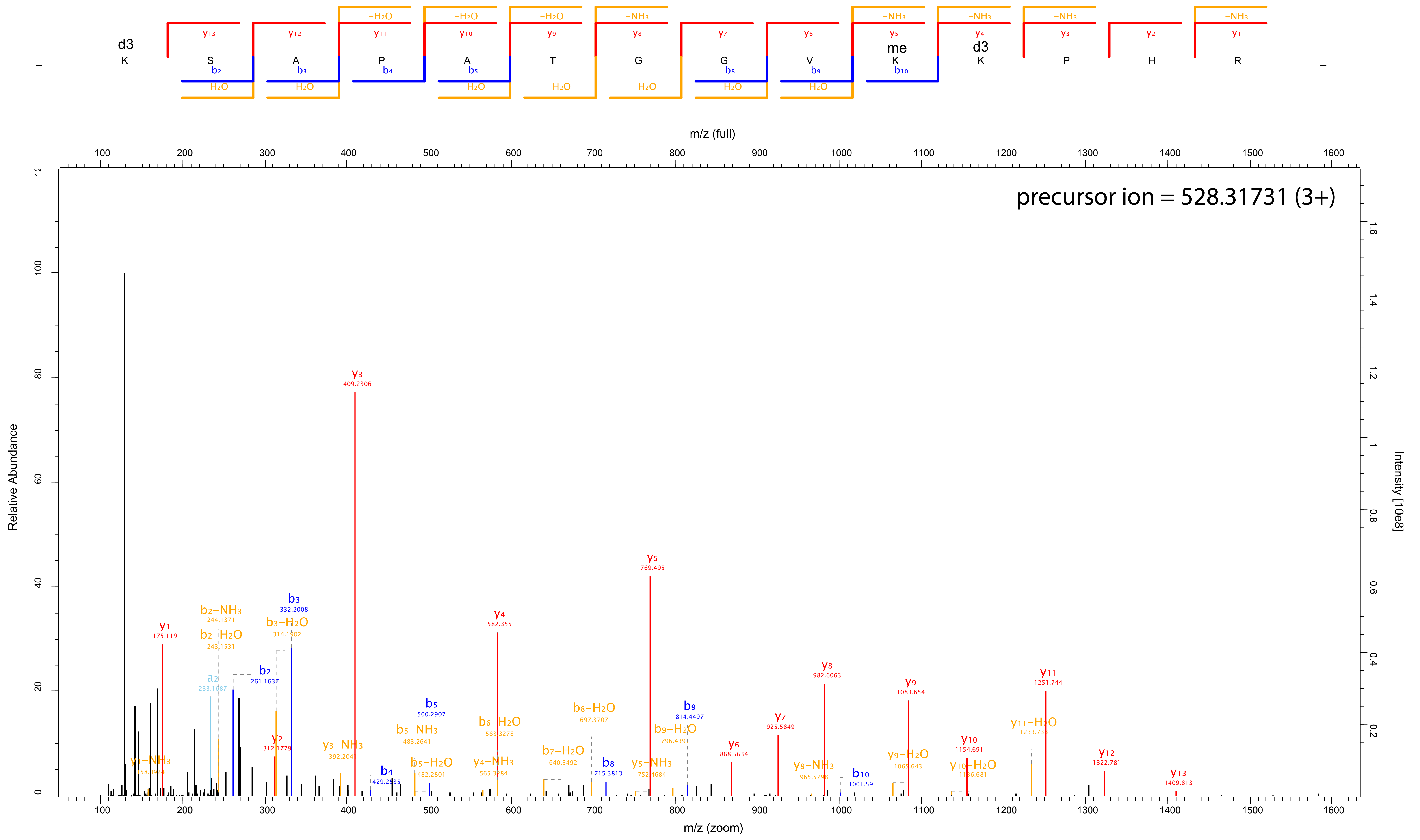
precursor ion = 528.31731 (3+)
m/z (full)
m/z (zoom)
Relative Abundance
Intensity [10e8]

H3 K37me1

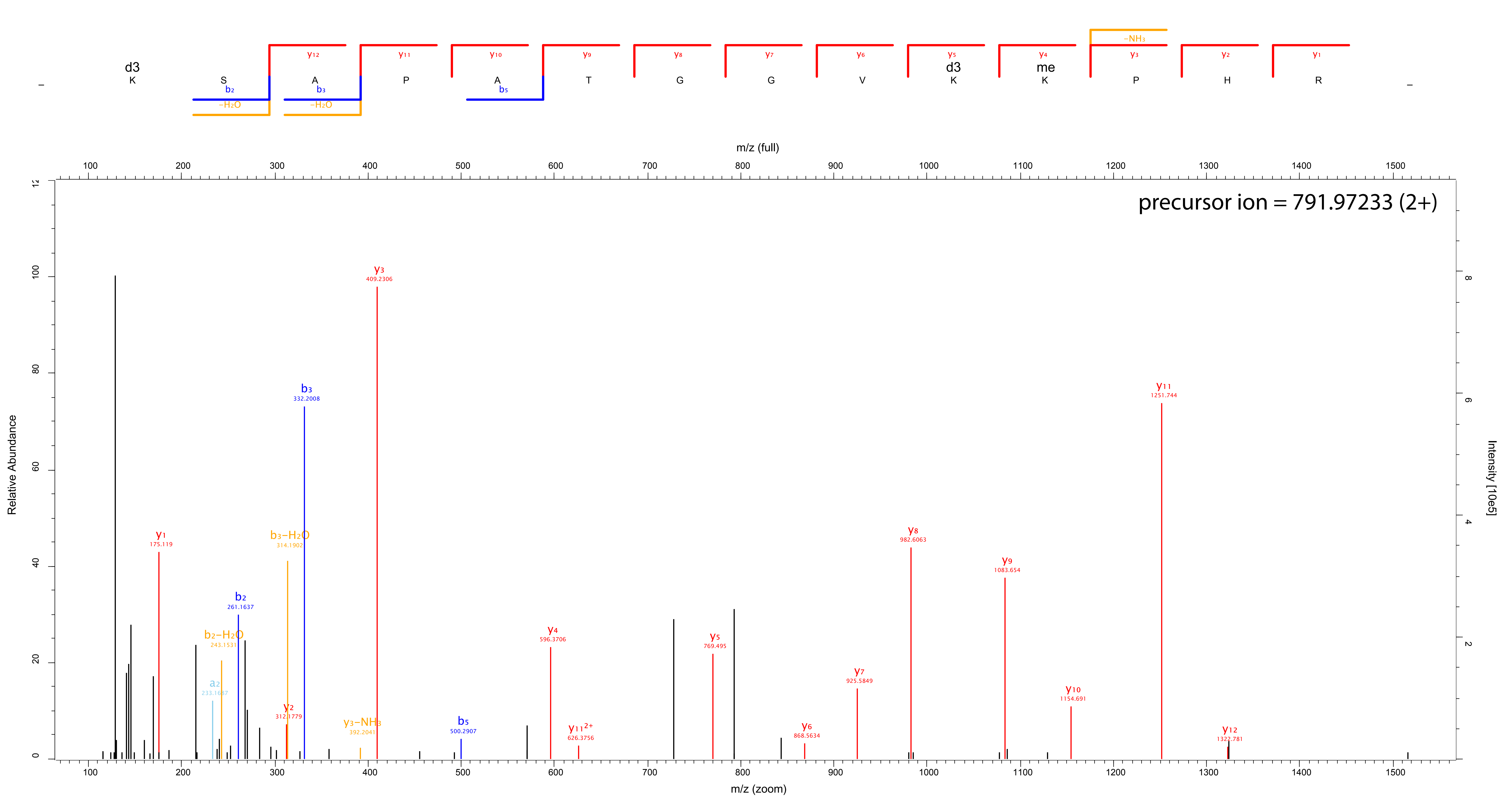

d3 K S A P A T G G V d3 K me K P H R
b2 b3 b5
-H2O -H2O
y12 y11 y10 y9 y8 y7 y6 y5 y4 y3 y2 y1
-NH3
m/z (full)
precursor ion = 791.97233 (2+)
Relative Abundance
Intensity [10e5]
m/z (zoom)
y3 409.2306
b3 332.2008
y11 1251.744
y1 175.119
b3-H2O 314.1902
y8 982.6063
y9 1083.654
b2 261.1637
b2-H2O 243.1531
y4 596.3706
y5 769.495
a2 233.1687
y2 312.1779
y7 925.5849
y10 1154.691
y3-NH3 392.2041
b5 500.2907
y11 2+ 626.3756
y6 868.5634
y12 1322.781

H3 K27me2

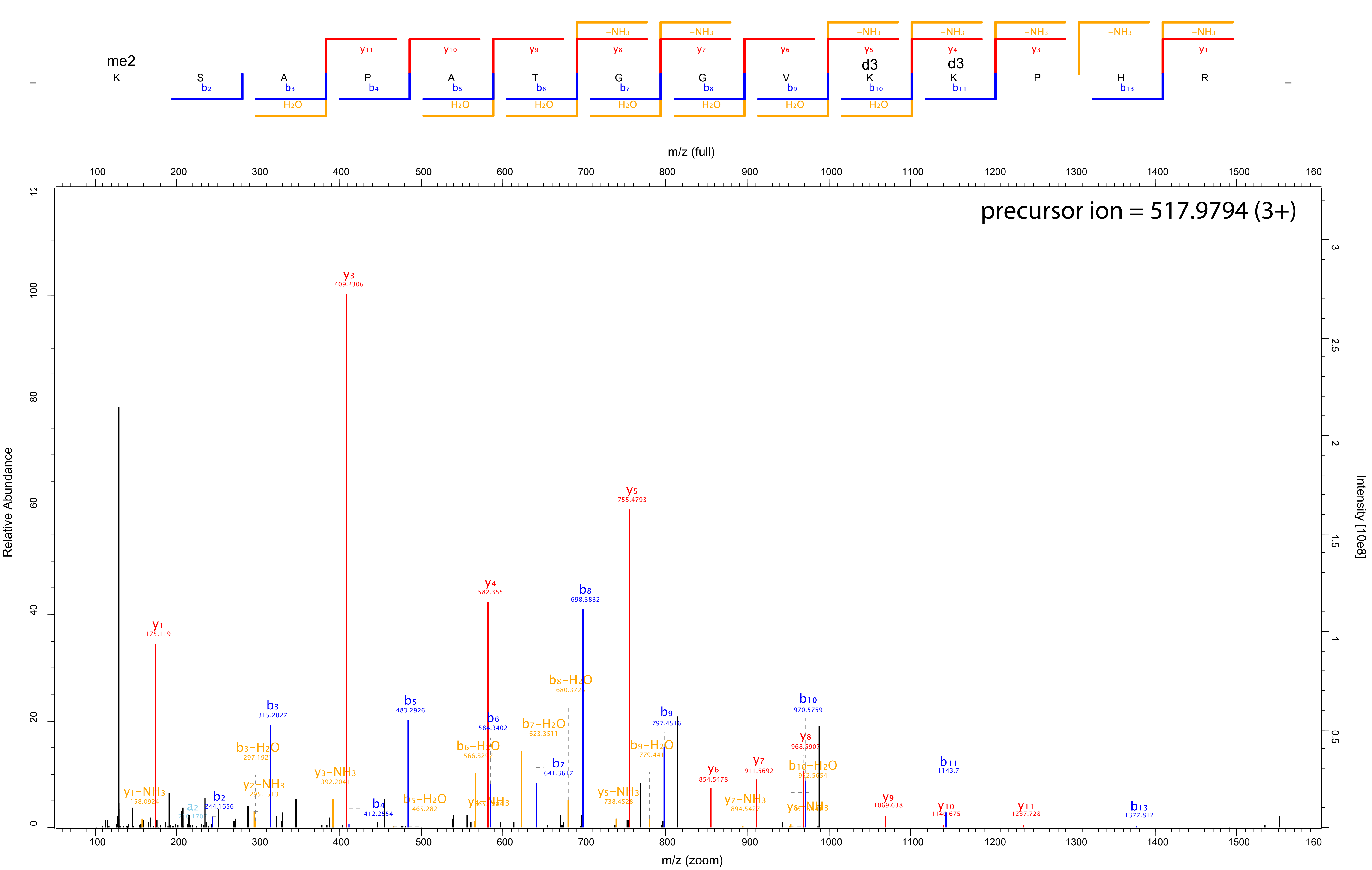

me2
K
S
A
P
A
T
G
G
V
d3
K
d3
K
P
H
R
m/z (full)
precursor ion = 517.9794 (3+)
Relative Abundance
Intensity [10e8]
m/z (zoom)
y3
409.2306
y5
755.4793
y4
582.355
b8
698.3832
y1
175.119
b8−H2O
680.3726
b3
315.2027
b5
483.2926
b6
584.3402
b7−H2O
623.3511
b9
797.4516
b10
970.5759
b3−H2O
297.192
b6−H2O
566.3297
b9−H2O
779.441
y8
968.5907
b10−H2O
952.5654
y3−NH3
392.2041
b7
641.3617
y6
854.5478
y7
911.5692
b11
1143.7
y1−NH3
158.0924
y2−NH3
295.1513
y5−NH3
738.4528
b2
244.1656
b4
412.2554
b5−H2O
465.282
y7−NH3
894.5427
y9
1069.638
y10
1140.675
y11
1237.728
b13
1377.812

H3 K36me2

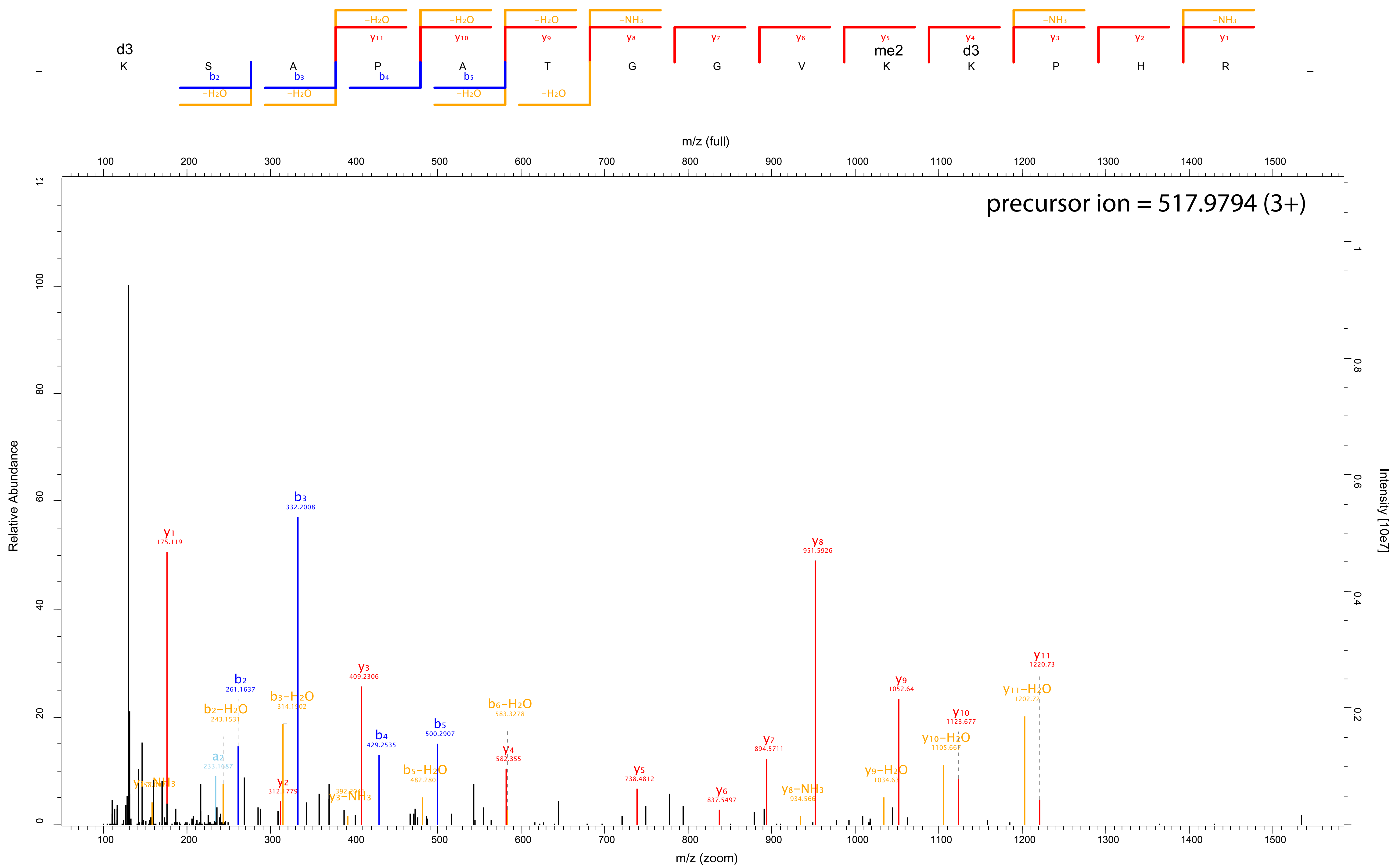

d3
K
S
A
P
A
T
G
G
V
me2
K
d3
K
P
H
R
b2
b3
b4
b5
y11
y10
y9
y8
y7
y6
y5
y4
y3
y2
y1
m/z (full)
precursor ion = 517.9794 (3+)
Relative Abundance
Intensity [10e7]
m/z (zoom)
y1 175.119
b3 332.2008
y8 951.5926
b2 261.1637
b2−H2O 243.1531
b3−H2O 314.1902
y3 409.2306
b4 429.2535
b5 500.2907
b6−H2O 583.3278
y4 582.355
b5−H2O 482.280
y2 312.1779
y5 738.4812
y6 837.5497
y7 894.5711
y8−NH3 934.566
y9 1052.64
y9−H2O 1034.63
y10 1123.677
y10−H2O 1105.667
y11 1220.73
y11−H2O 1202.72

H3 K27me3

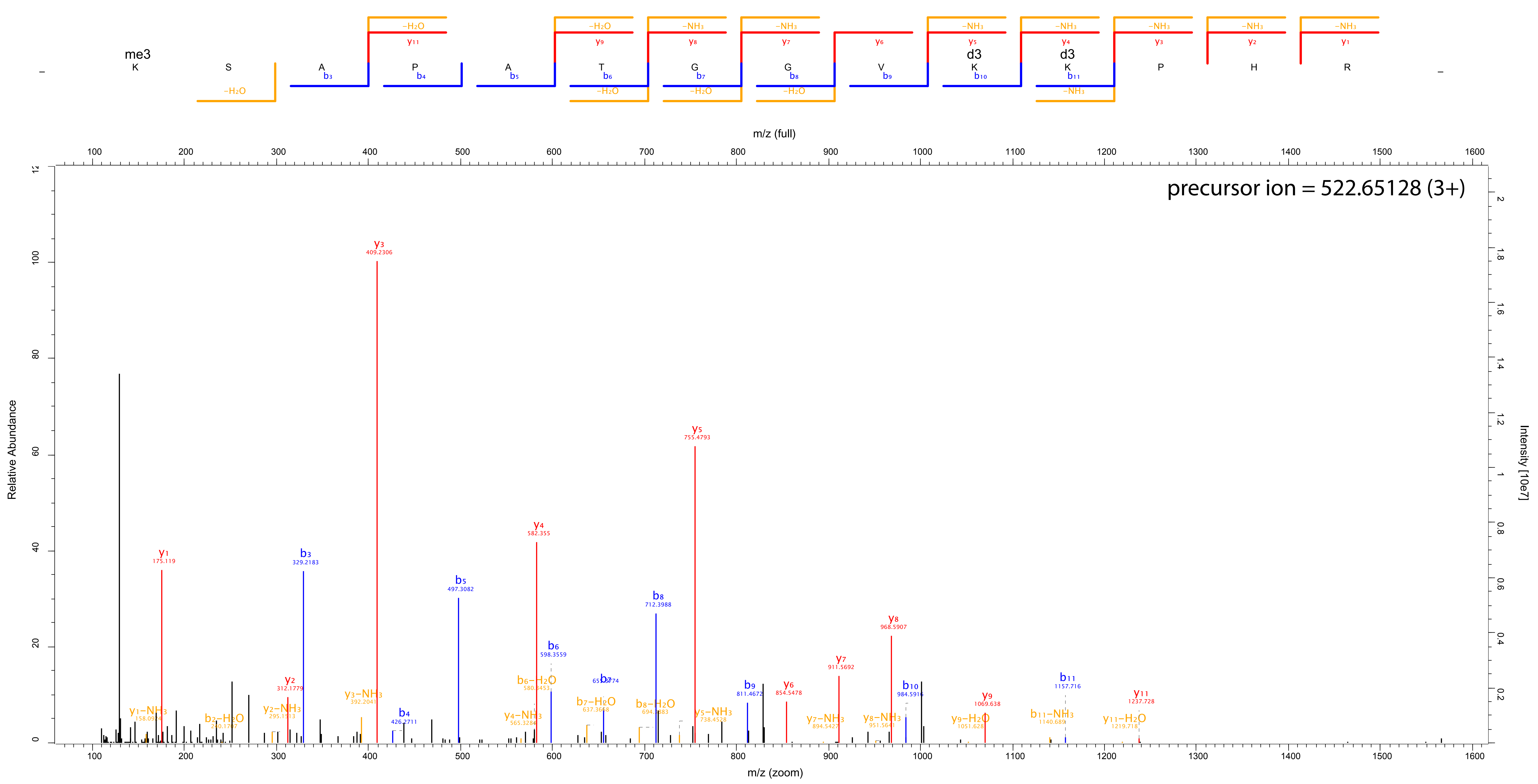

me3
K S A P A T G G V K K P H R
d3
d3
m/z (full)
precursor ion = 522.65128 (3+)
Relative Abundance
Intensity [10e7]
m/z (zoom)
y3 409.2306
y5 755.4793
y4 582.355
y1 175.119
b3 329.2183
b5 497.3082
b8 712.3988
y8 968.5907
b6 598.3559
y7 911.5692
y2 312.1779
y6 854.5478
b9 811.4672
b10 984.5916
b11 1157.716
y9 1069.638
y11 1237.728
b4 426.2711

H3 K36me3

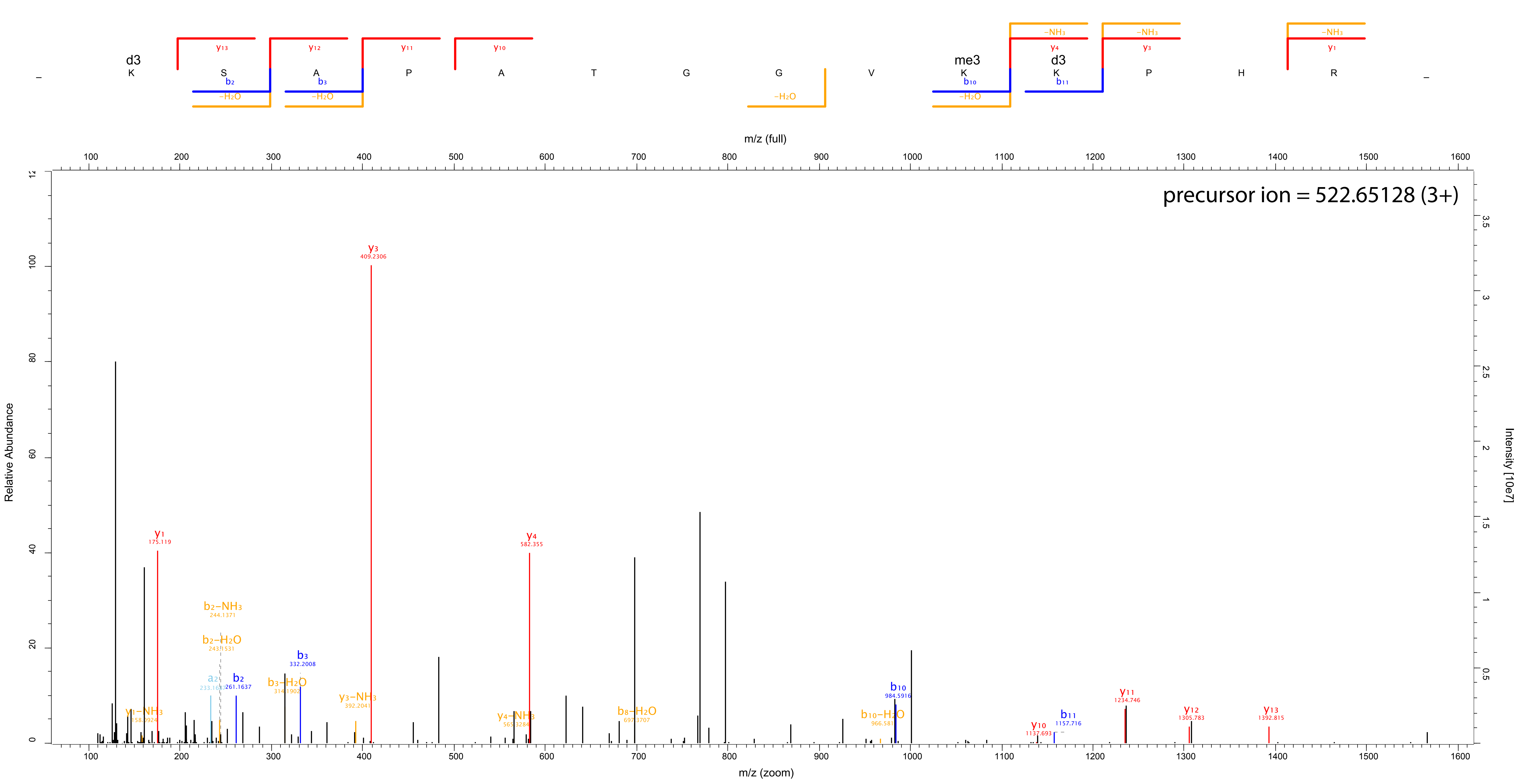

d3 K S A P A T G G V me3 K d3 K P H R
y13 y12 y11 y10 y4 y3 y1
-NH3
b2 b3 b10 b11
-H2O
m/z (full)
precursor ion = 522.65128 (3+)
Relative Abundance
Intensity [10e7]
m/z (zoom)
y3 409.2306
y1 175.119
y4 582.355
b2-NH3 244.1371
b2-H2O
b3 332.2008
b2 261.1637
b3-H2O 314.1902
y3-NH3 392.2041
y4-NH3
b8-H2O 697.3707
b10 984.5916
b10-H2O 966.581
y11 1234.746
y12 1305.783
y13 1392.815
b11 1157.716
y10 1137.693

H3 K27me1K36me1

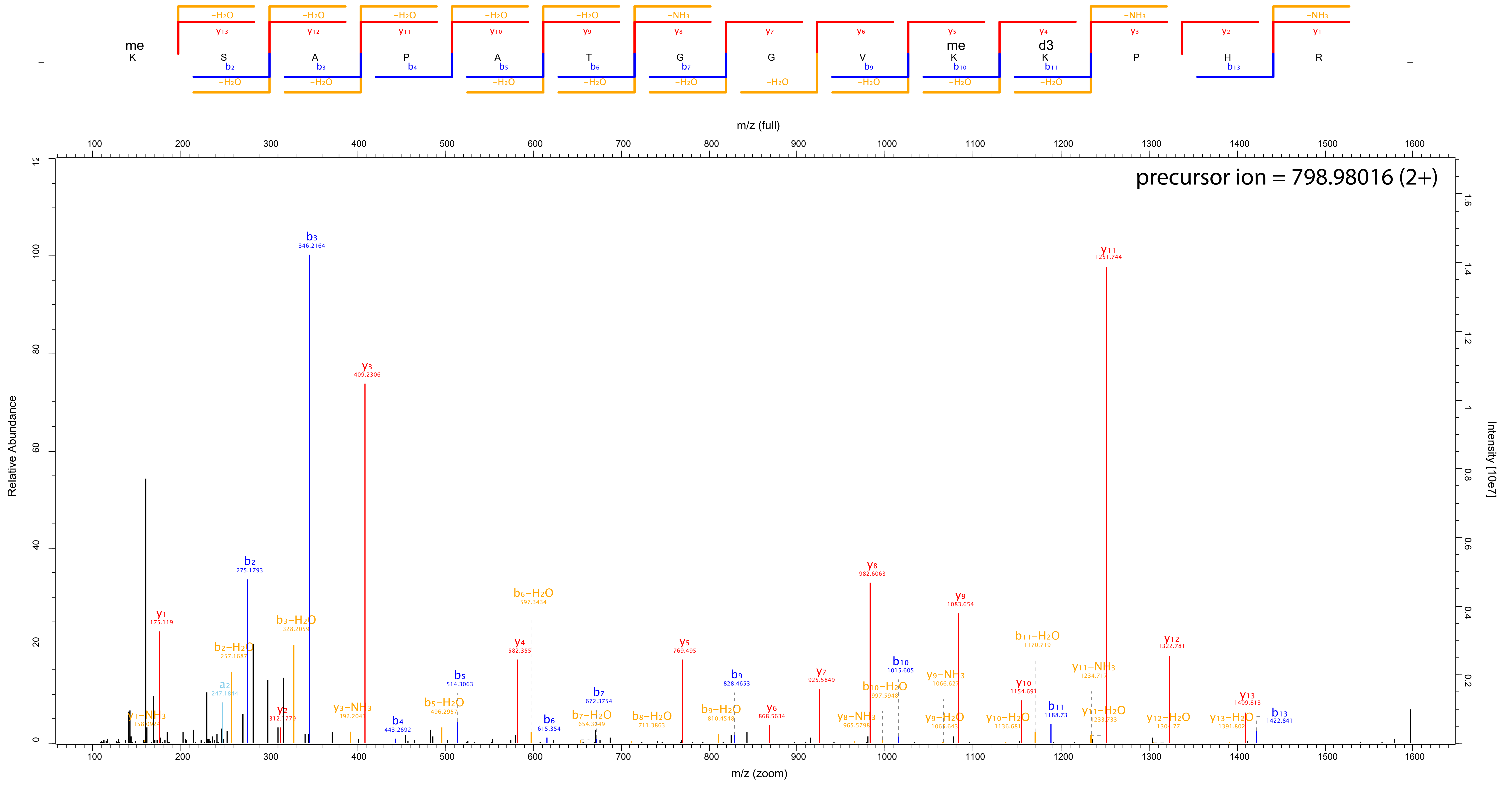

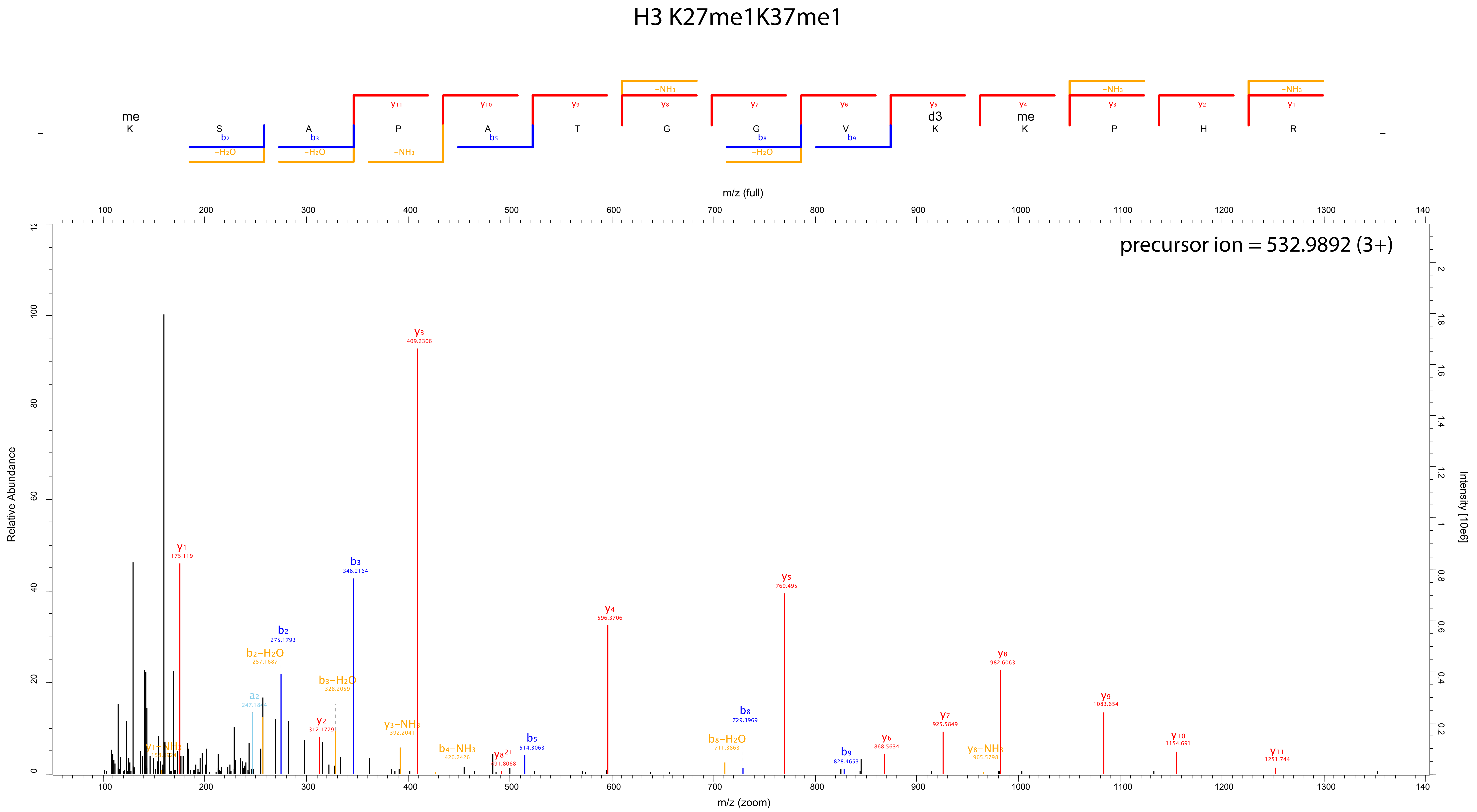
H3 K27me1K37me1
precursor ion = 532.9892 (3+)
m/z (full)
m/z (zoom)
Relative Abundance
Intensity [10e6]

H3 K27me2K36me1

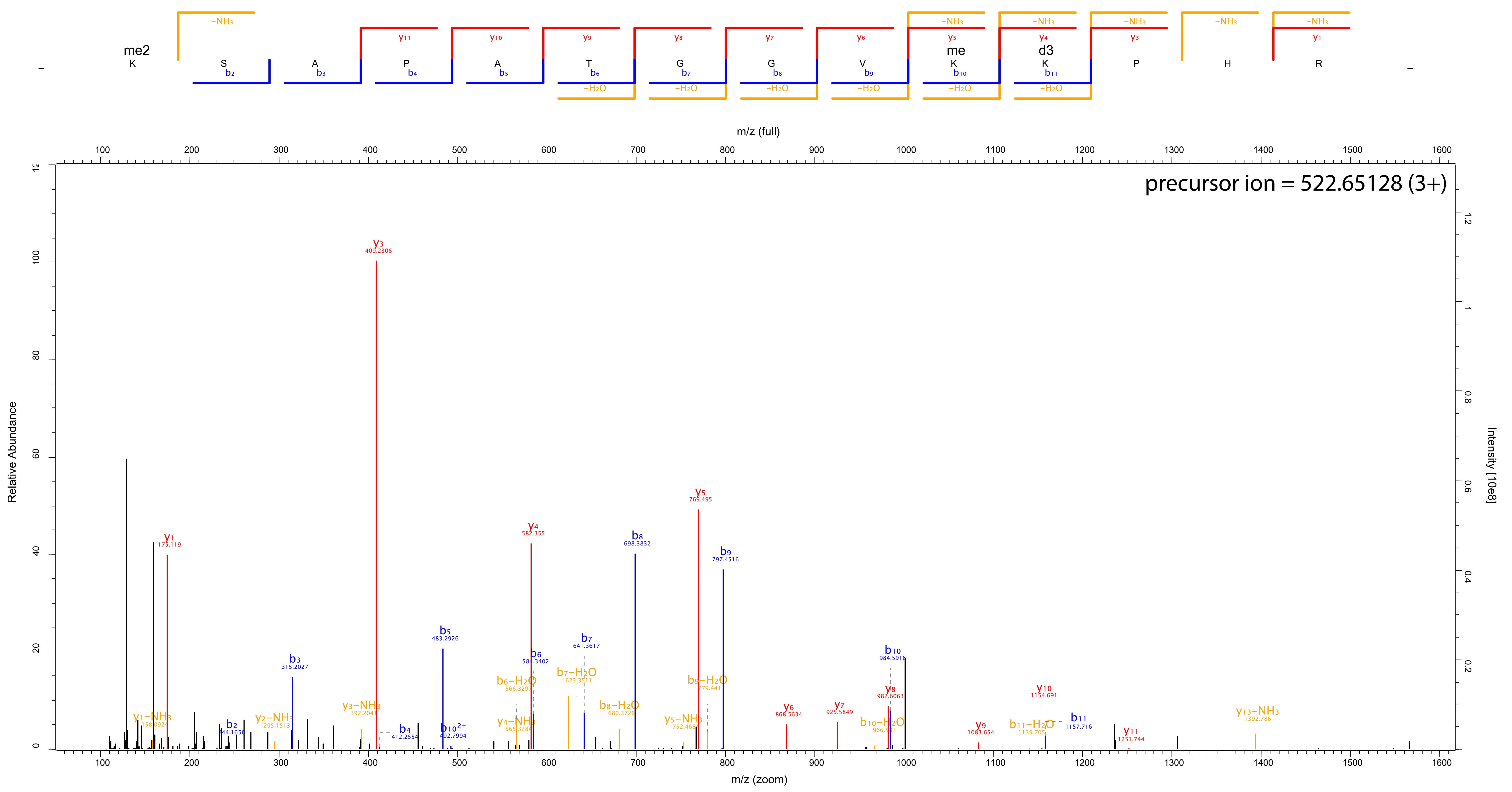

H3 K27me1K36me2

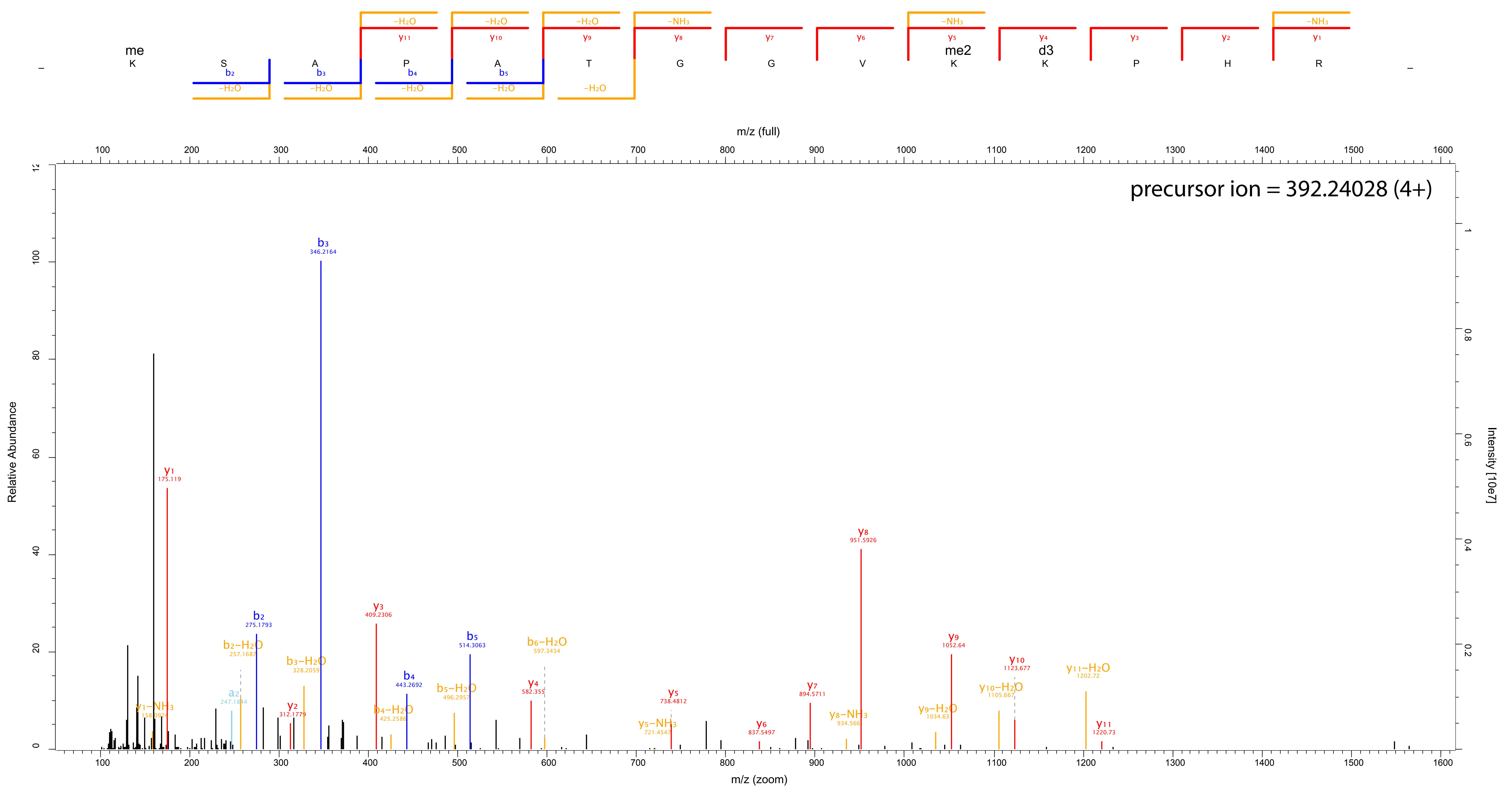

H3 K27me2K37me1

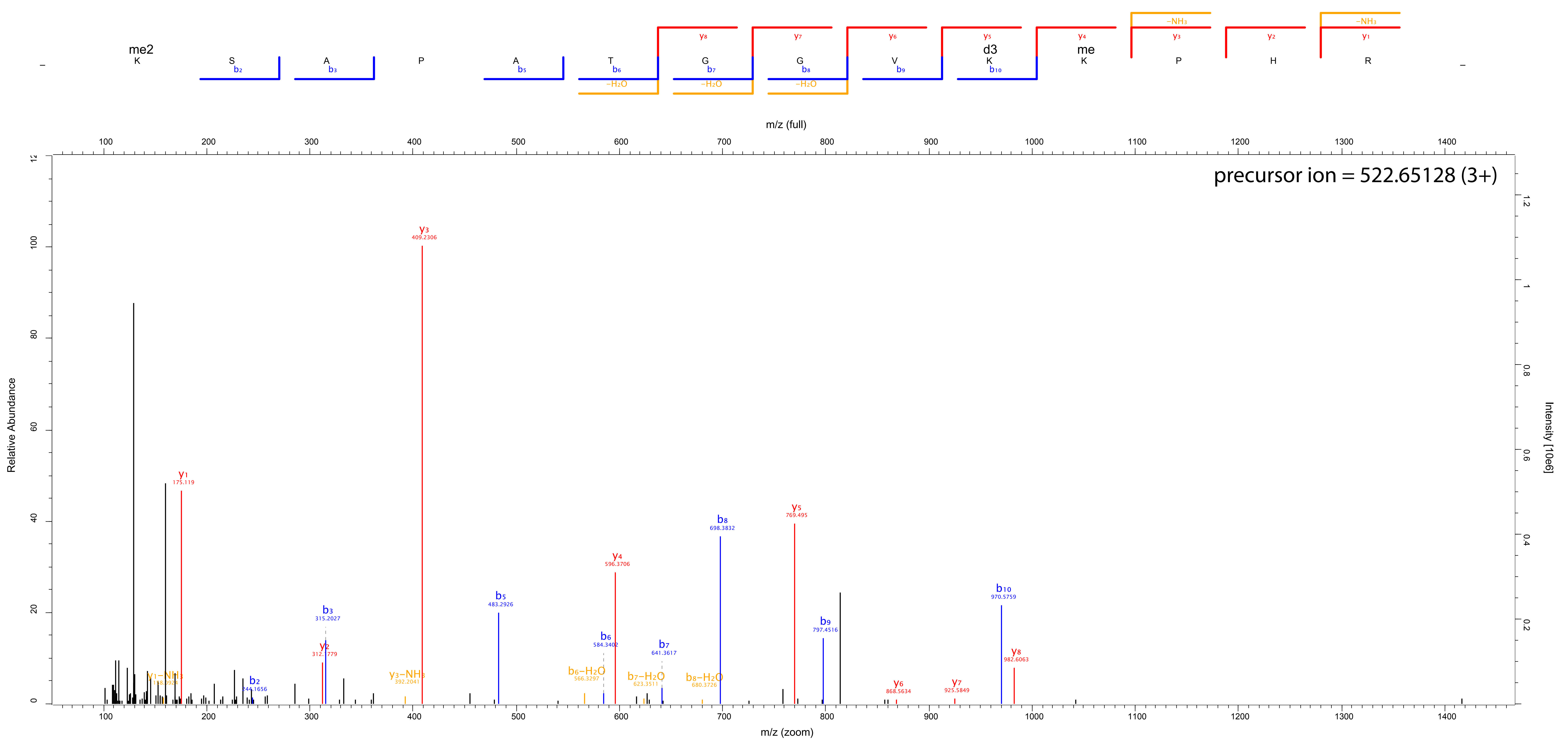

H3 K27me1K36me3

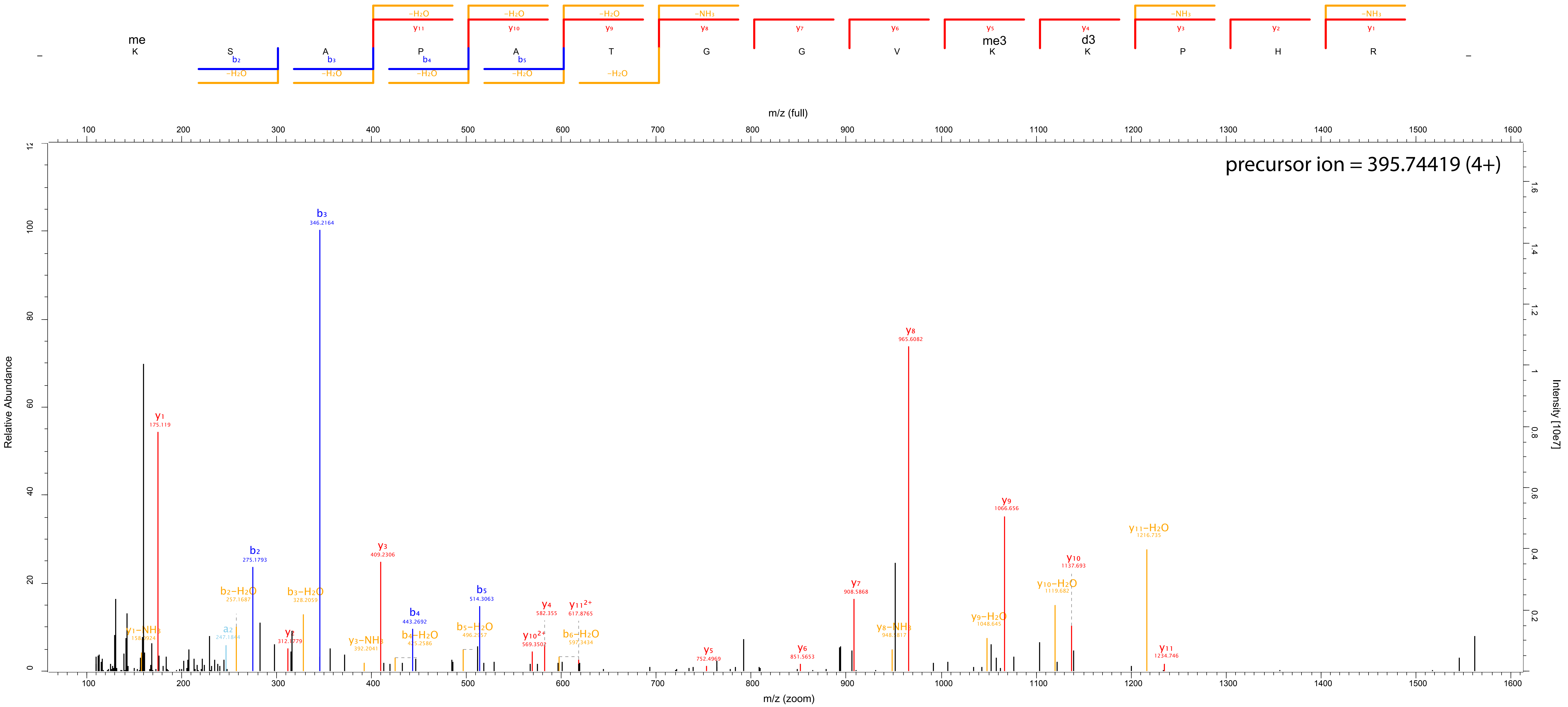

H3 K27me3K36me1

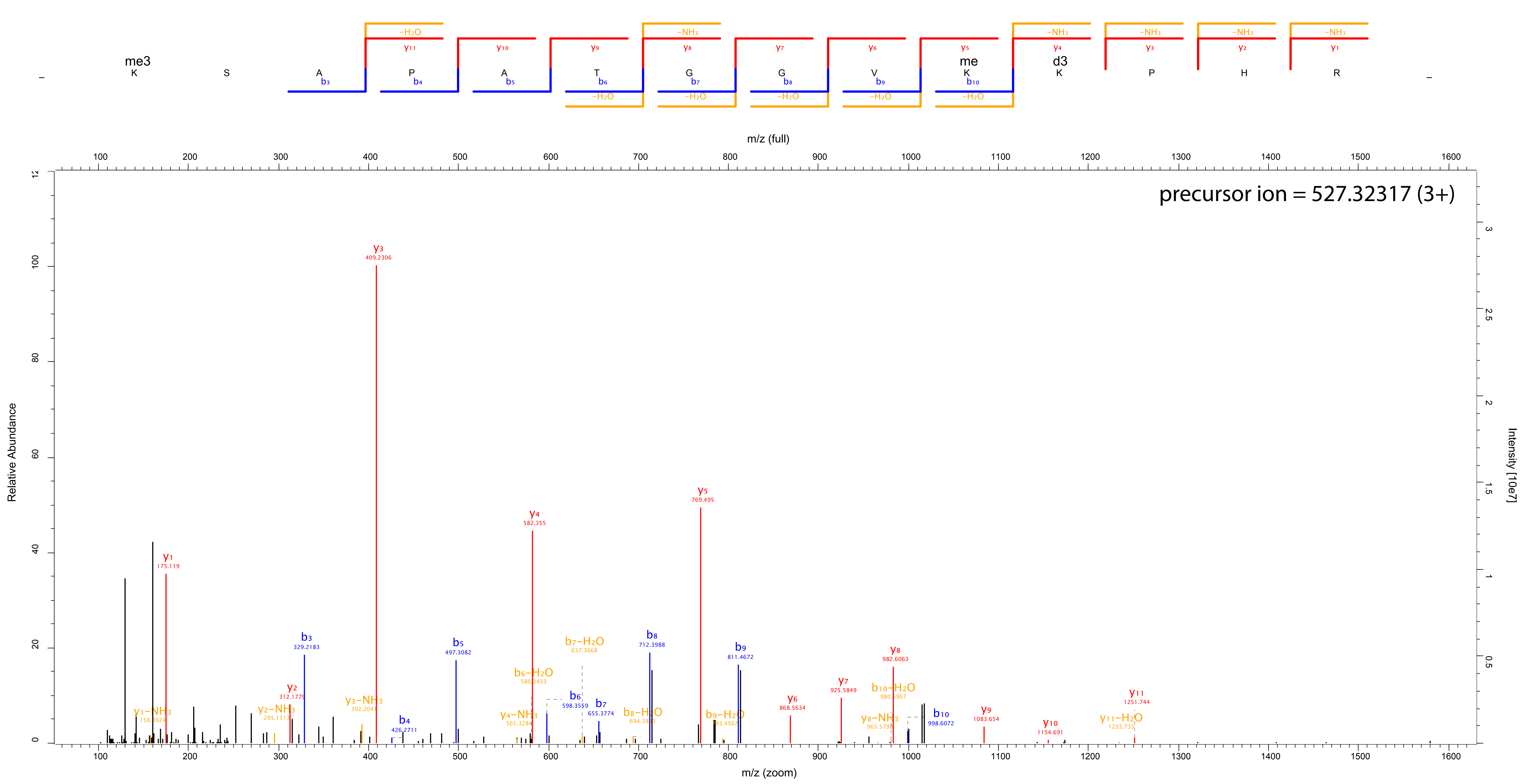

H3 K27me2K36me2

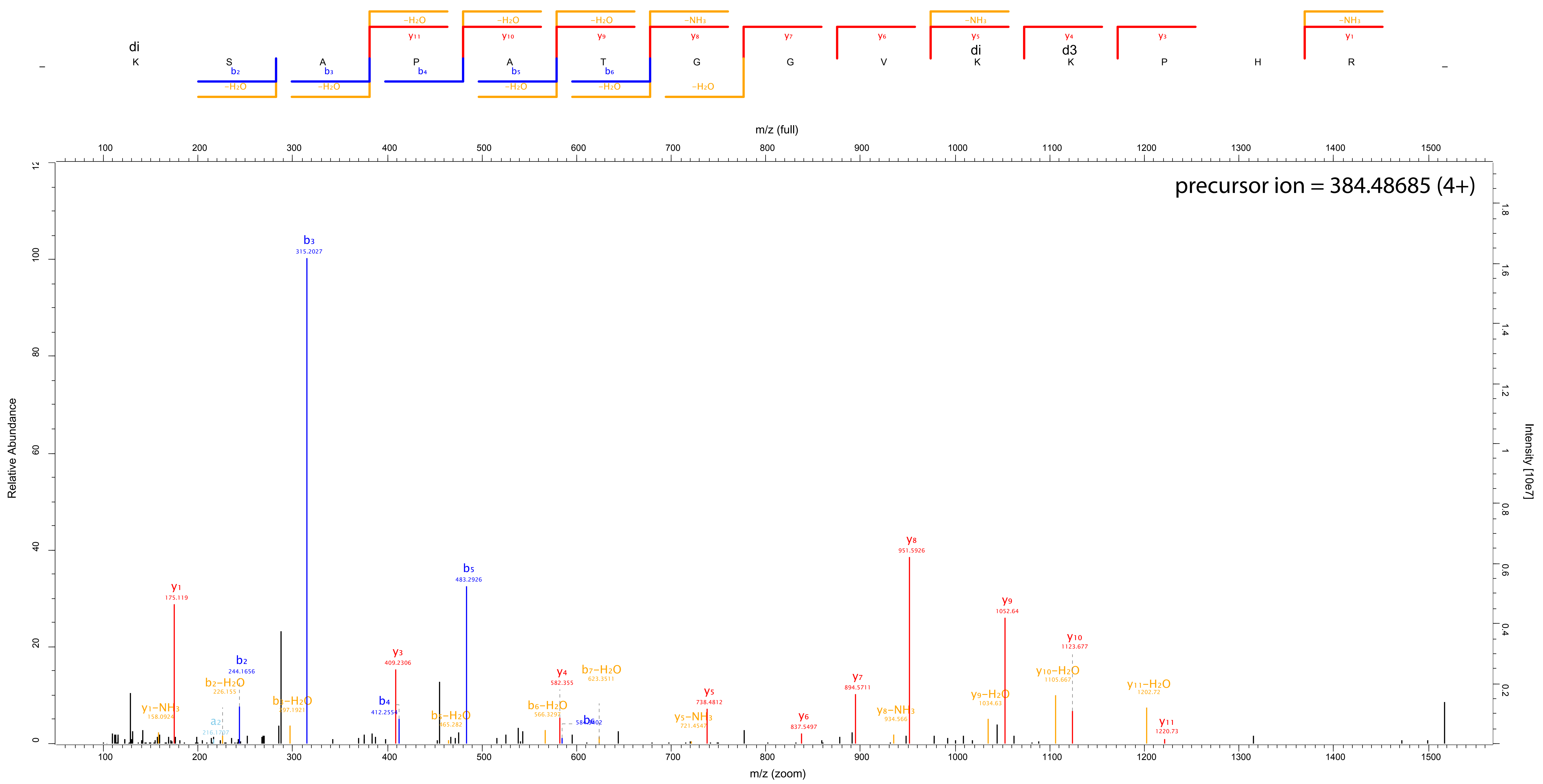

H3 K27me1K36me1K37me1

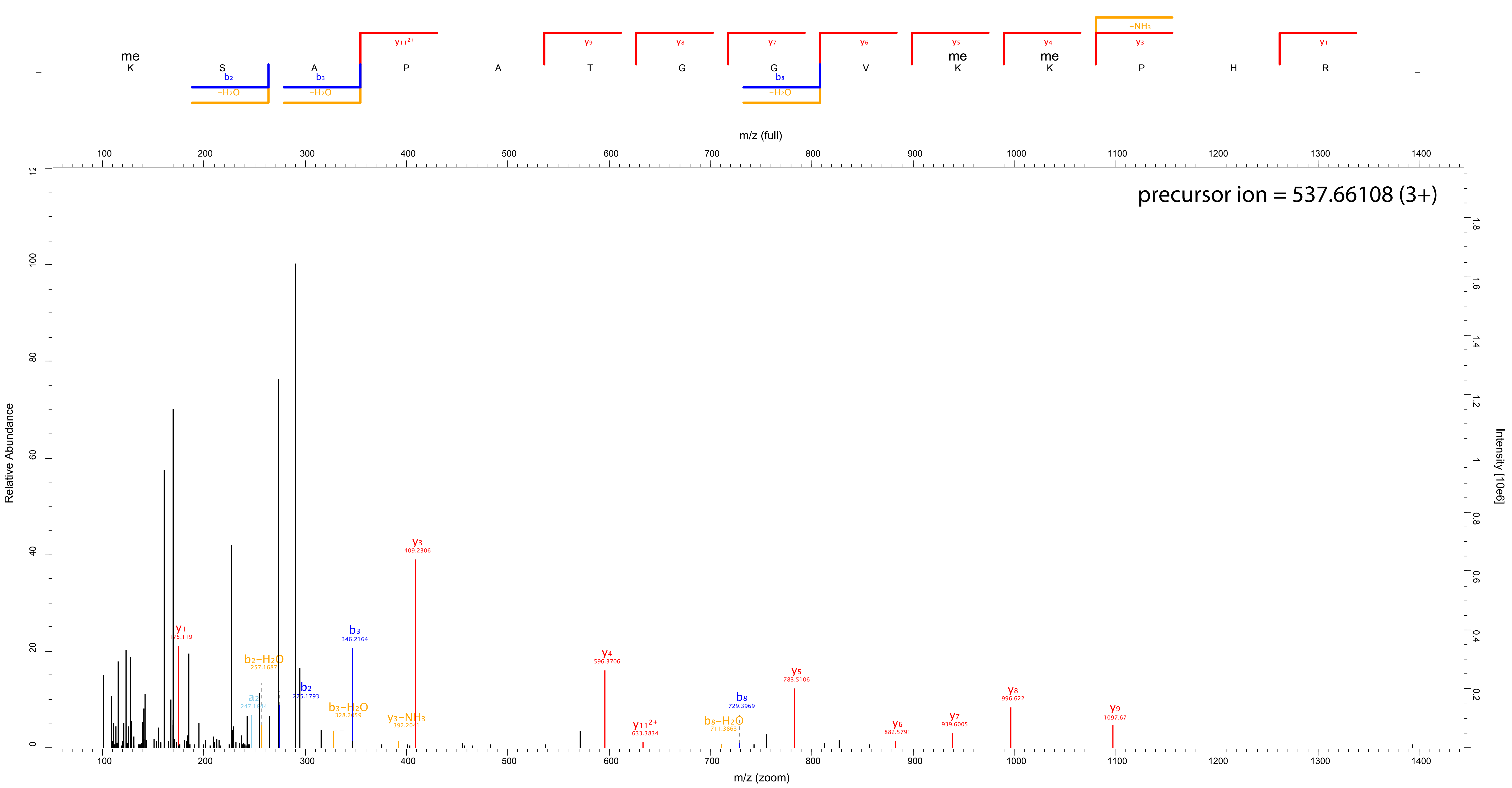

H3 K27me1K36me2K37me1

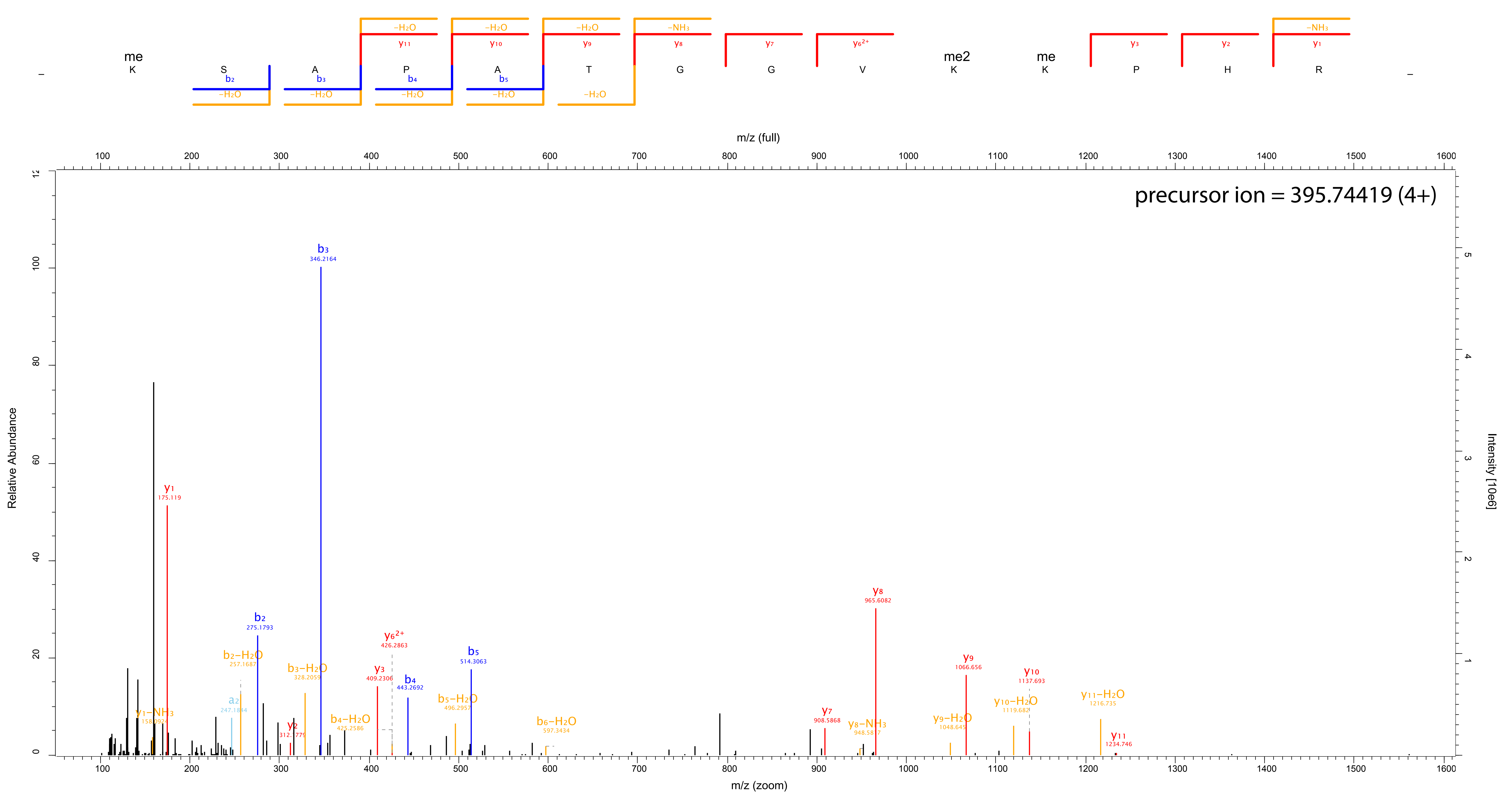

H3 K27me2K36me1K37me2

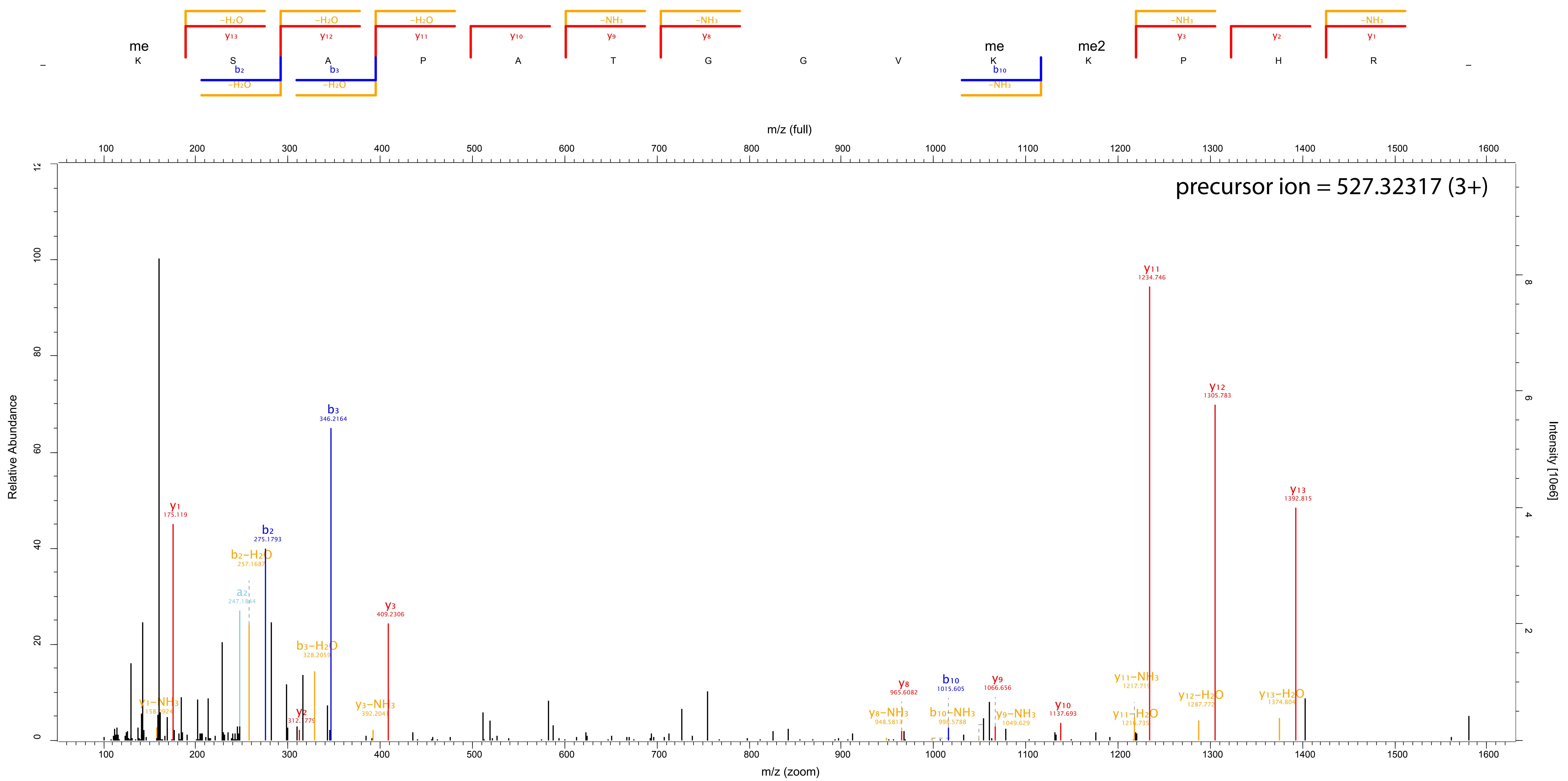

H3 K27me2K36me1K37me1

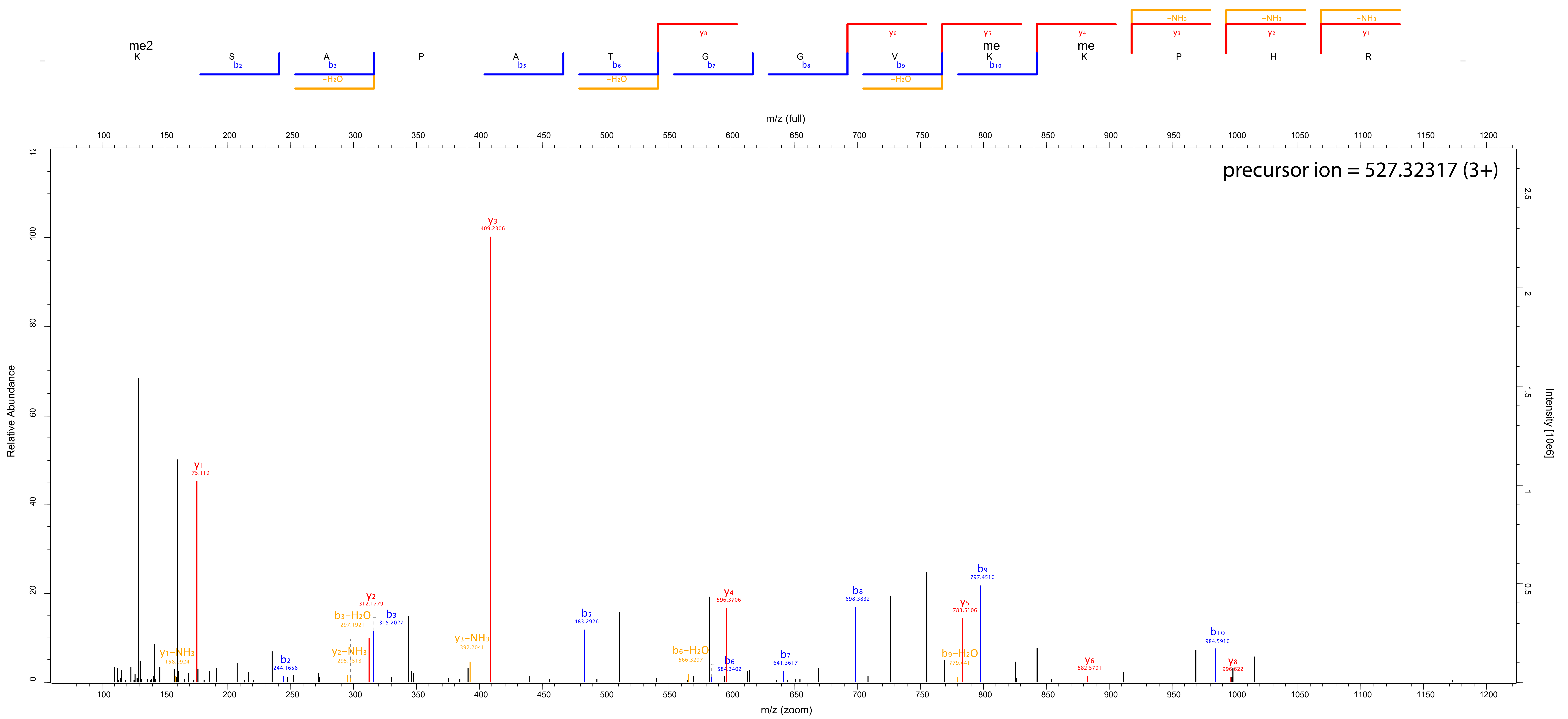

H3 K27ac

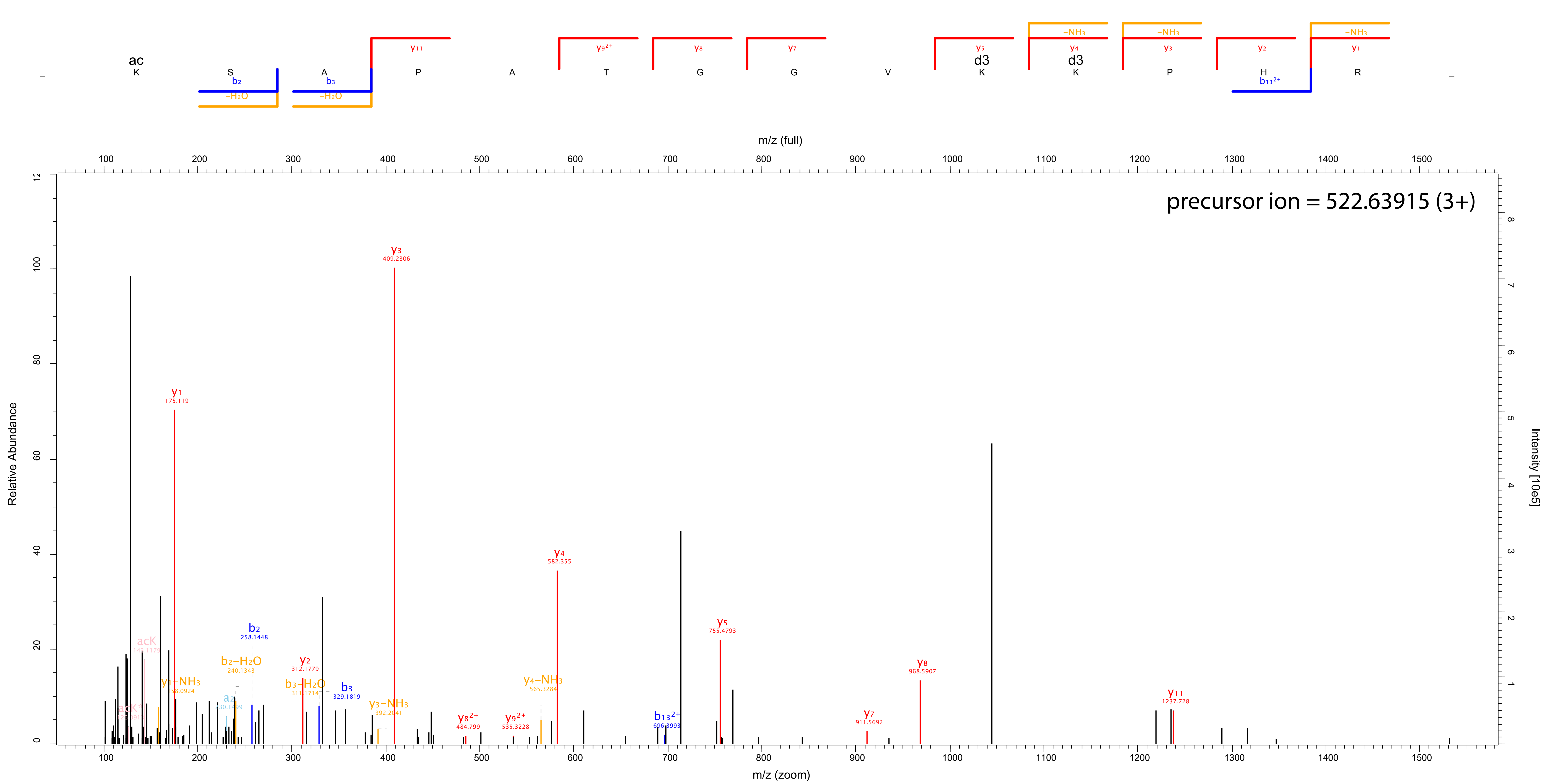

ac
K S A P A T G G V K K P H R
d3
d3
b2
b3
-H2O
y11
y9 2+
y8
y7
y5
y4
y3
y2
y1
-NH3
b13 2+
m/z (full)
precursor ion = 522.63915 (3+)
y3
409.2306
y1
175.119
y4
582.355
y5
755.4793
y8
968.5907
y11
1237.728
y7
911.5692
y2
312.1779
b2
258.1448
b3
329.1819
b2-H2O
240.1343
b3-H2O
y1-NH3
y3-NH3
y4-NH3
565.3284
y8 2+
484.799
y9 2+
535.3228
b13 2+
acK
Relative Abundance
Intensity [10e5]
m/z (zoom)

H3 K27me1K36ac

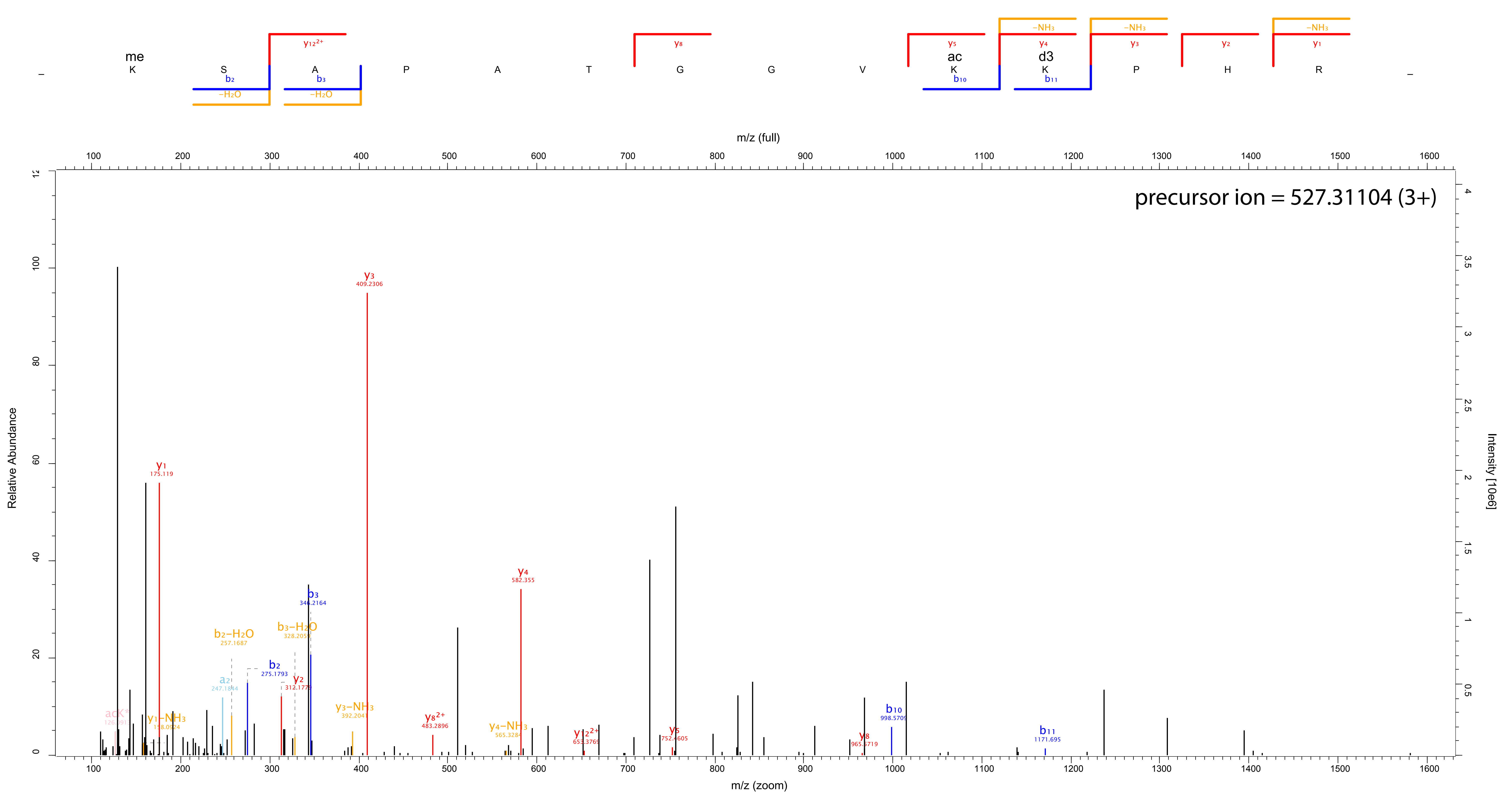

H3 K27me1K37ac

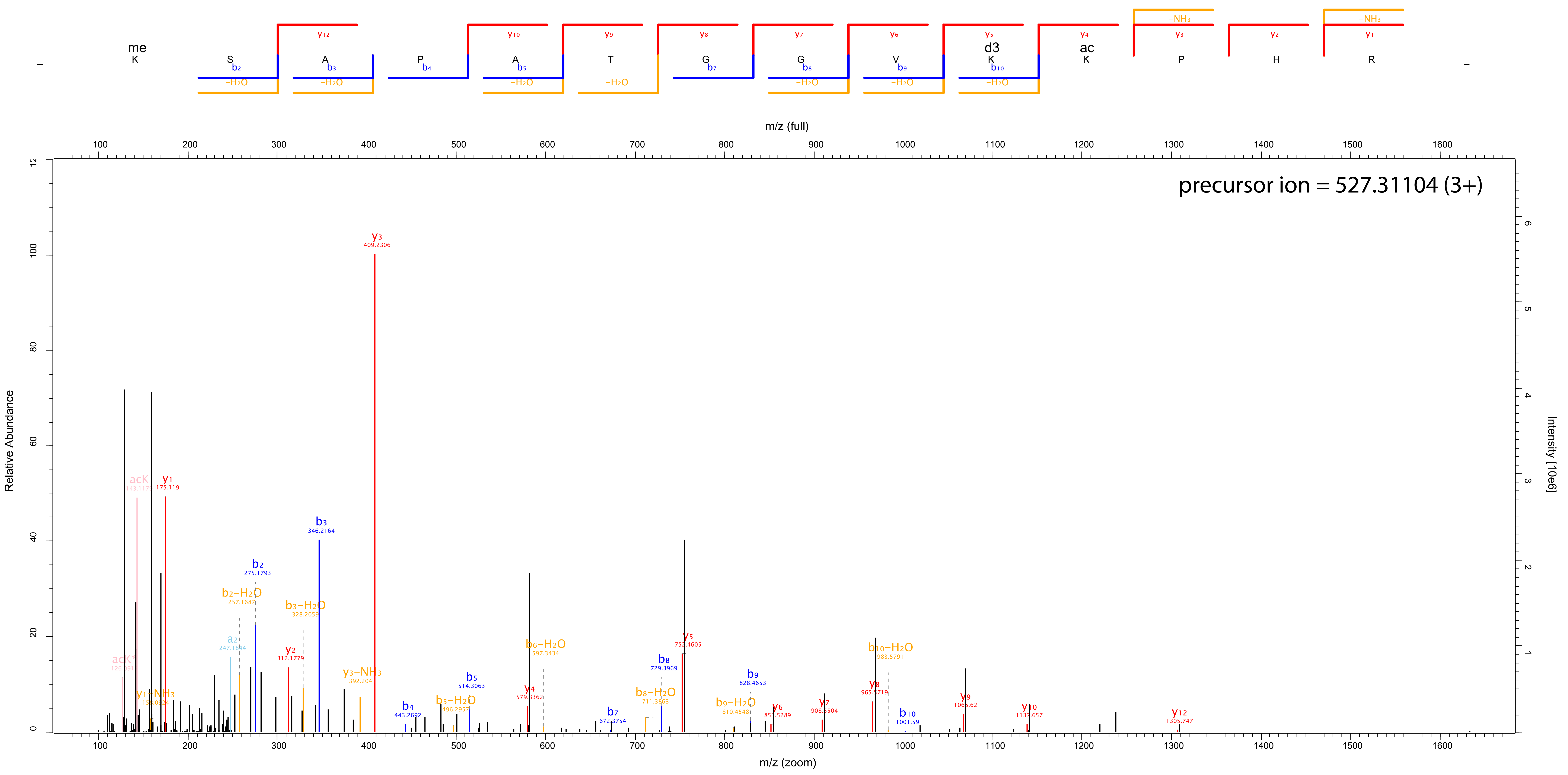

H3 K36form

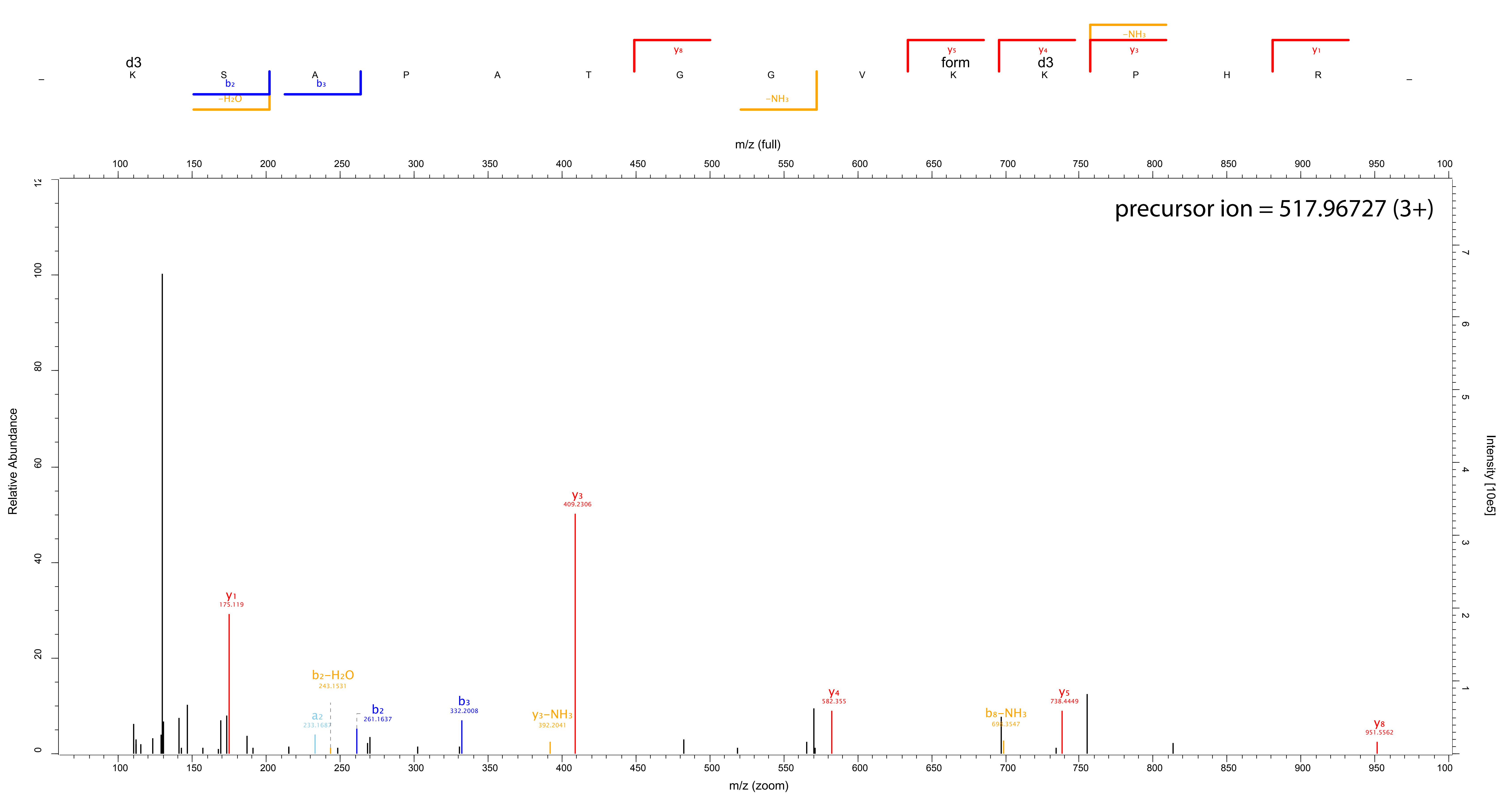

precursor ion = 517.96727 (3+)
d3
form
d3
K S A P A T G G V K K P H R
b₂
b₃
-H₂O
-NH₃
y₈
y₅
y₄
y₃
y₁
m/z (full)
m/z (zoom)
Relative Abundance
Intensity [10e5]
y₁ 175.119
b₂-H₂O 243.1531
a₂ 233.1687
b₂ 261.1637
b₃ 332.2008
y₃-NH₃ 392.2041
y₃ 409.2306
y₄ 582.355
b₈-NH₃
y₅ 738.4449
y₈ 951.5562

H3 K79me1

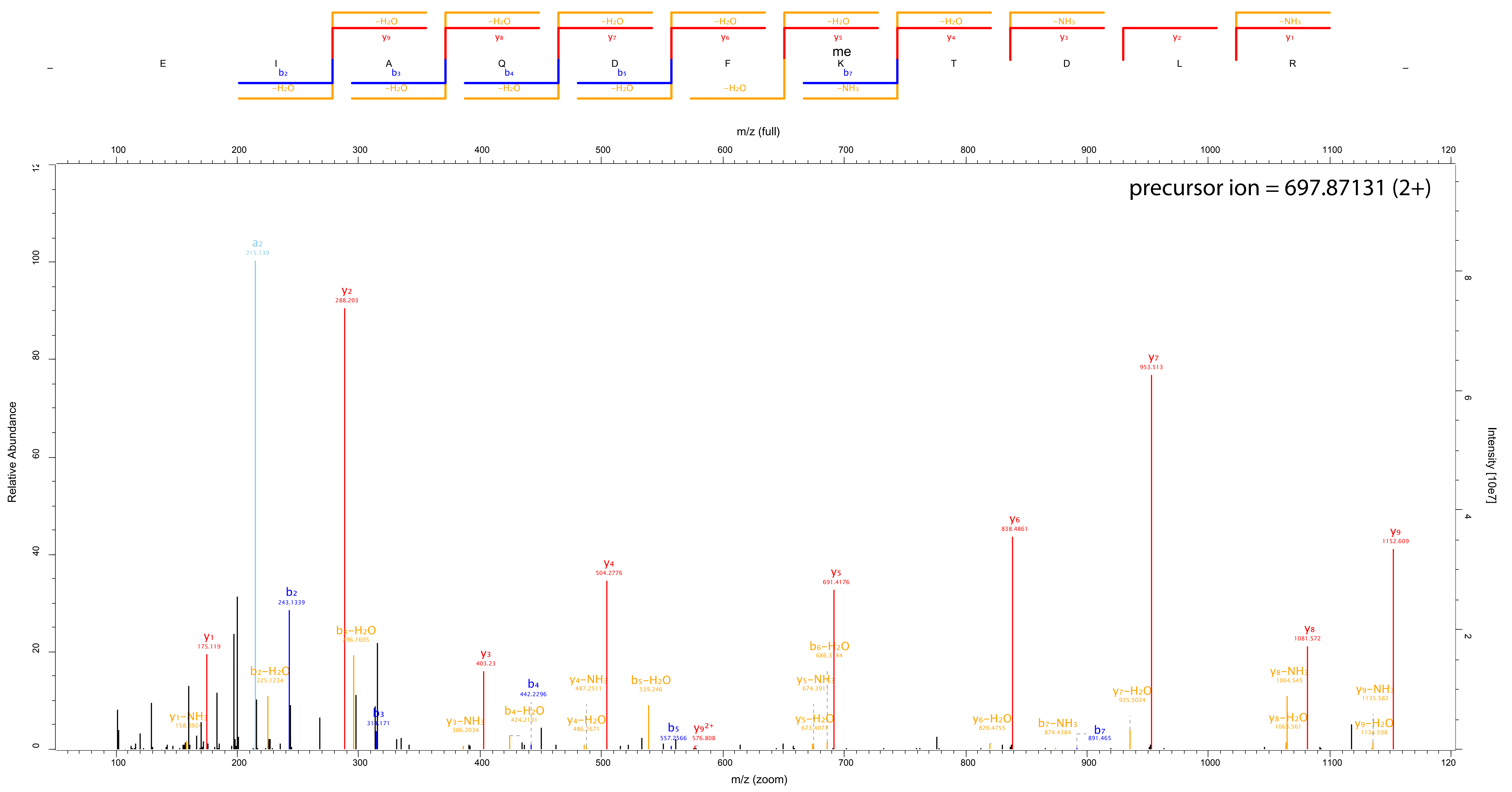

E I A Q D F K T D L R
me
m/z (full)
precursor ion = 697.87131 (2+)
Relative Abundance
Intensity [10e7]
m/z (zoom)
a2 215.139
y2 288.203
y7 953.513
y6 838.4861
y9 1152.609
y4 504.2776
y5 691.4176
b2 243.1339
y1 175.119
y3 403.23
y8 1081.572
b4 442.2296
b5 557.2566
b7 891.465

H3 K79me2

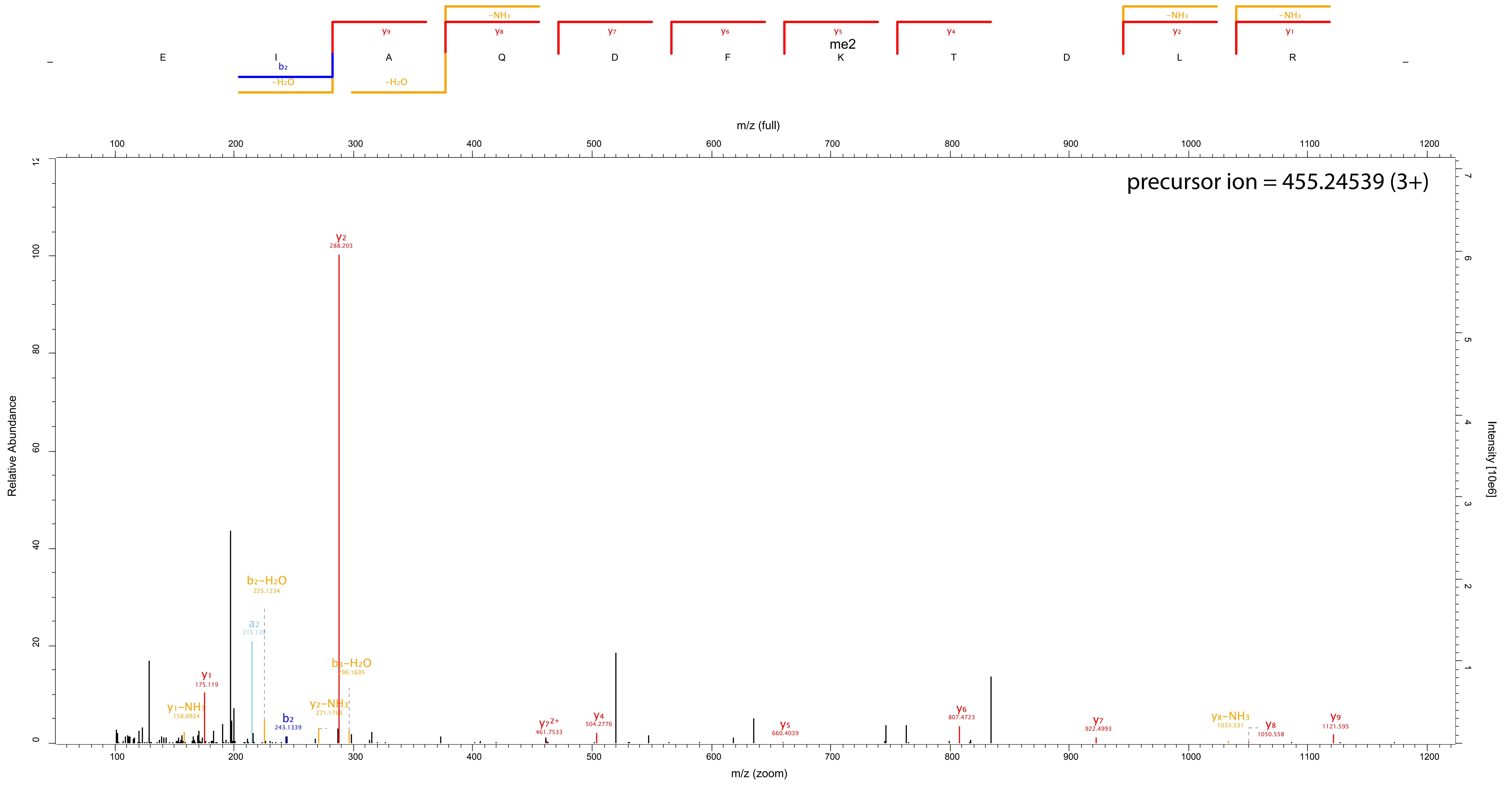

E I A Q D F K T D L R
me2
b₂
−H₂O
y9 y8 y7 y6 y5 y4 y2 y1
−NH₃
m/z (full)
precursor ion = 455.24539 (3+)
Relative Abundance
Intensity [10e6]
y₂ 288.203
b₂−H₂O 225.1234
a₂ 215.139
y₁ 175.119
y₁−NH₃ 158.0924
b₂ 243.1339
y₂−NH₃ 271.1765
b₃−H₂O 296.1605
y₇²⁺ 461.7533
y₄ 504.2776
y₅ 660.4039
y₆ 807.4723
y₇ 922.4993
y₈−NH₃ 1033.531
y₈ 1050.558
y₉ 1121.595
m/z (zoom)

H3 K79ac

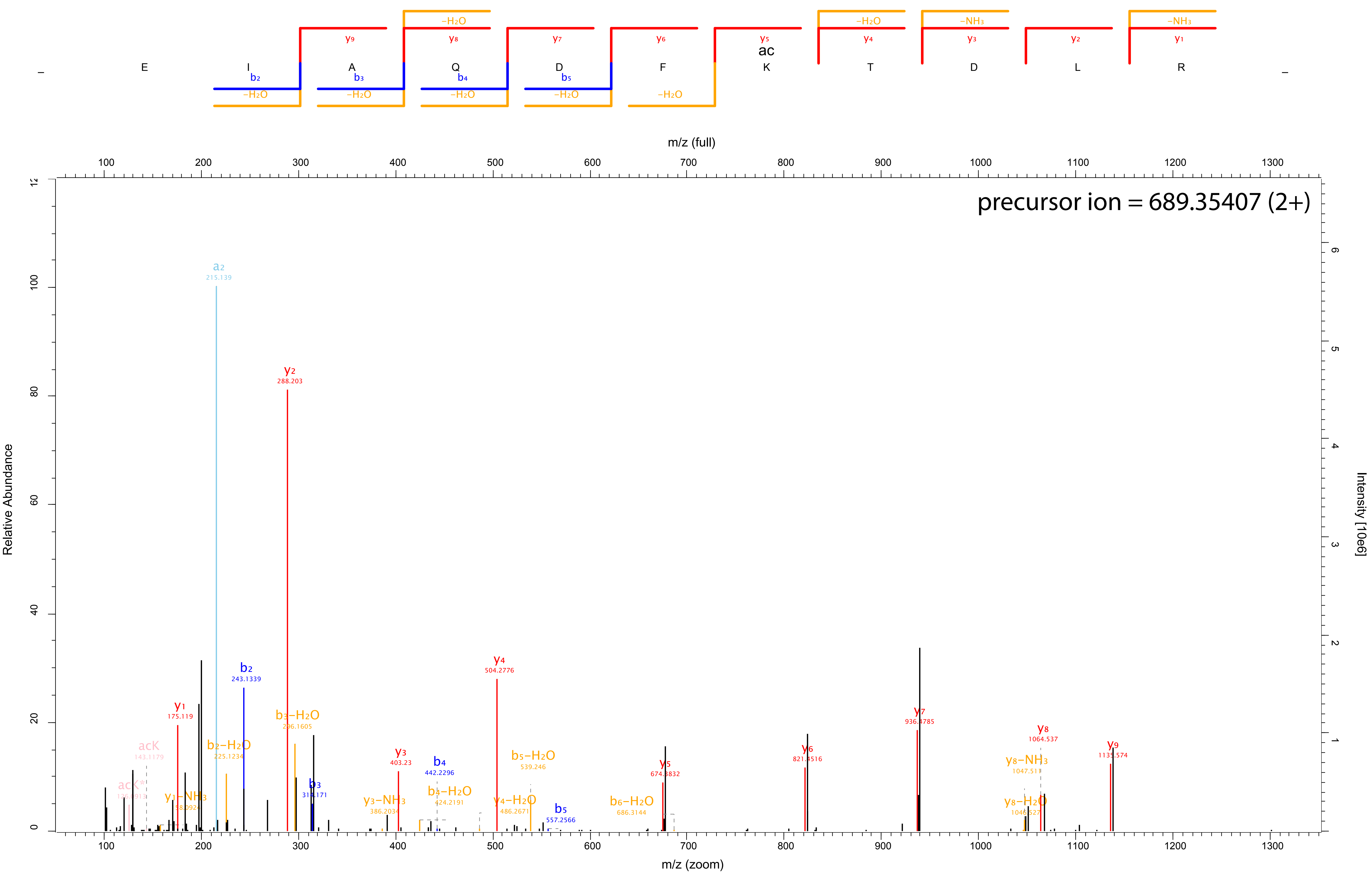

E I A Q D F K T D L R
ac
y9 y8 y7 y6 y5 y4 y3 y2 y1
b2 b3 b4 b5
-H2O
-NH3
m/z (full)
precursor ion = 689.35407 (2+)
Relative Abundance
Intensity [10e6]
m/z (zoom)
a2 215.139
y2 288.203
b2 243.1339
y1 175.119
acK 143.1179
b2-H2O 225.1234
b3-H2O 296.1605
b3 314.171
y3 403.23
b4 442.2296
y4 504.2776
b5-H2O 539.246
y3-NH3 386.2034
b4-H2O 424.2191
y4-H2O 486.2671
b5 557.2566
b6-H2O 686.3144
y5 674.3832
y6 821.4516
y7 936.4785
y8 1064.537
y8-NH3 1047.511
y8-H2O
y9 1135.574

H3 K79form

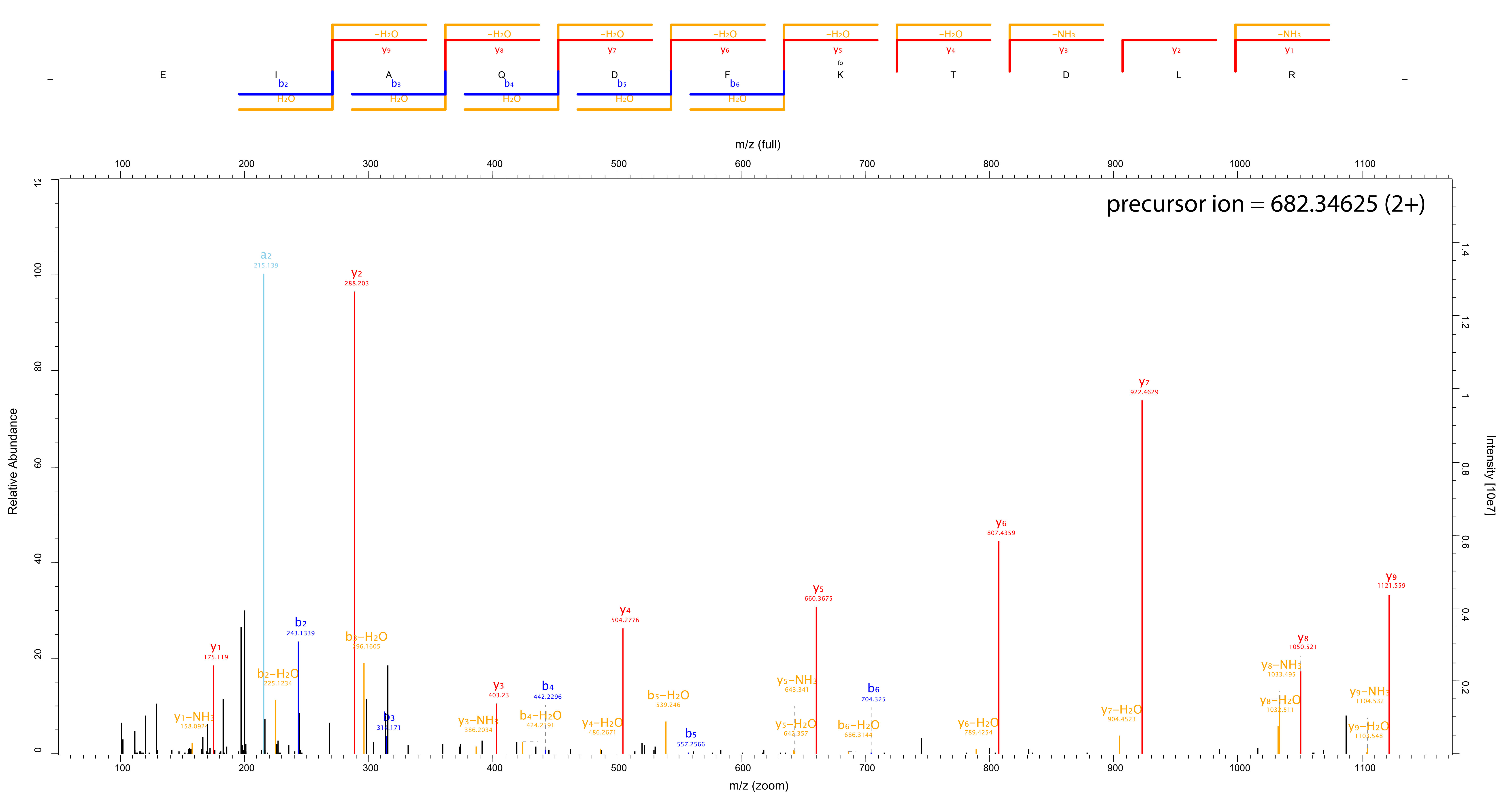

E I A Q D F K T D L R
fo
precursor ion = 682.34625 (2+)
m/z (full)
m/z (zoom)
Relative Abundance
Intensity [10e7]
a2 215.139
y2 288.203
y7 922.4629
y6 807.4359
y5 660.3675
y4 504.2776
y9 1121.559
y8 1050.521
b2 243.1339
y1 175.119
b3-H2O 296.1605
b2-H2O 225.1234
y3 403.23
b4 442.2296
b6 704.325
b5-H2O 539.246
y5-NH3 643.341
y8-NH3 1033.495
y9-NH3 1104.532
y1-NH3 158.0924
y3-NH3 386.2034
b4-H2O 424.2191
y4-H2O 486.2671
y5-H2O 642.357
b6-H2O 686.3144
y6-H2O 789.4254
y7-H2O 904.4523
b5 557.2566

H4 K16ac

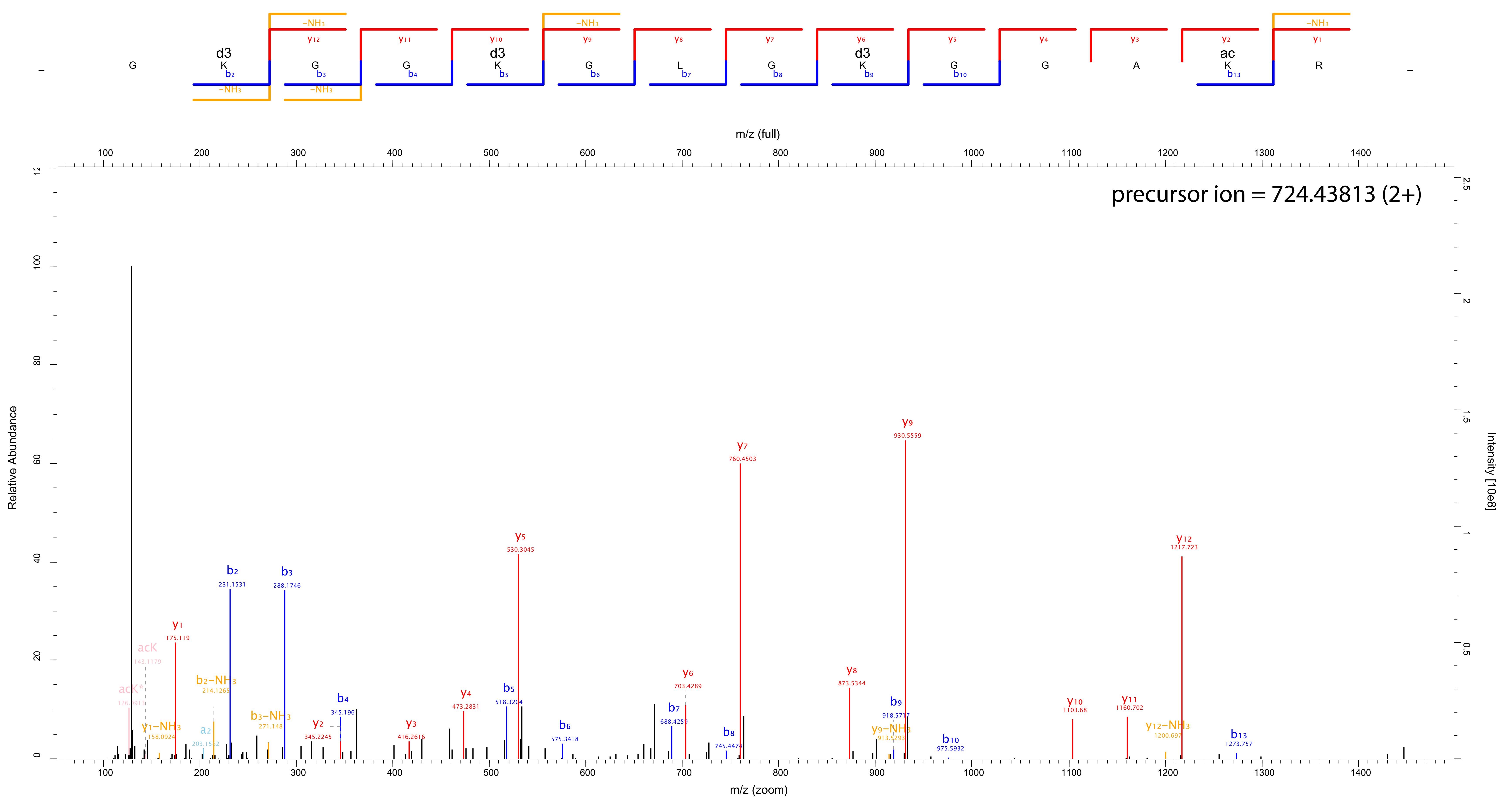

precursor ion = 724.43813 (2+)
m/z (full)
m/z (zoom)
Relative Abundance
Intensity [10e8]

H4 K12ac

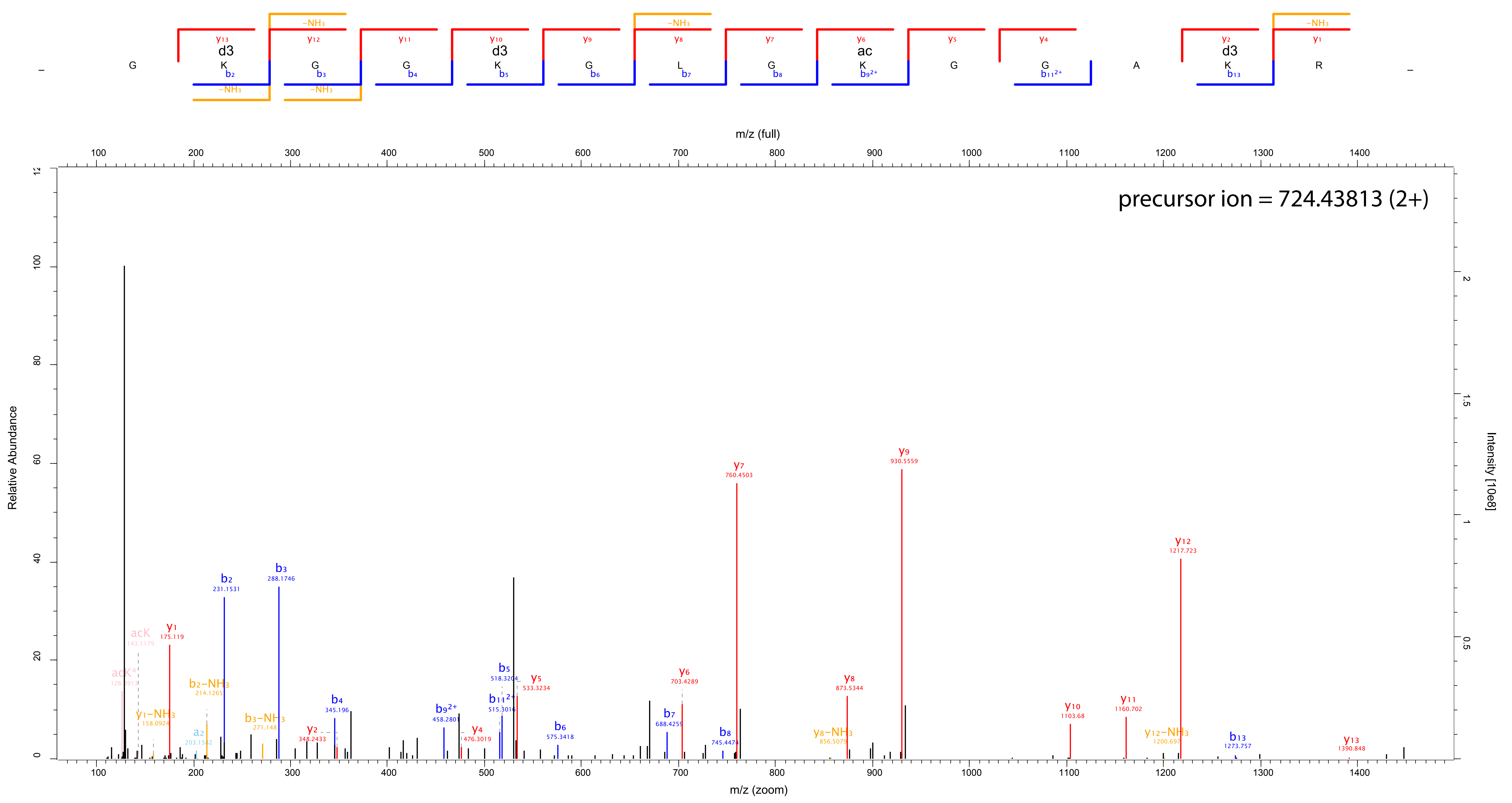

precursor ion = 724.43813 (2+)
m/z (full)
m/z (zoom)
Relative Abundance
Intensity [10e8]
G K G G K G L G K G G A K R
y1 175.119
y2 348.2433
y4 476.3019
y5 533.3234
y6 703.4289
y7 760.4503
y8 873.5344
y9 930.5559
y10 1103.68
y11 1160.702
y12 1217.723
y13 1390.848
b2 231.1531
b3 288.1746
b4 345.196
b5 518.3204
b6 575.3418
b7 688.4259
b8 745.4474
b9 2+ 458.2801
b11 2+ 515.3016
b13 1273.757
b2−NH3 214.1265
b3−NH3 271.148
y1−NH3 158.0924
y8−NH3 856.5079
y12−NH3 1200.697
a2 203.1532
acK 143.1179
acK*

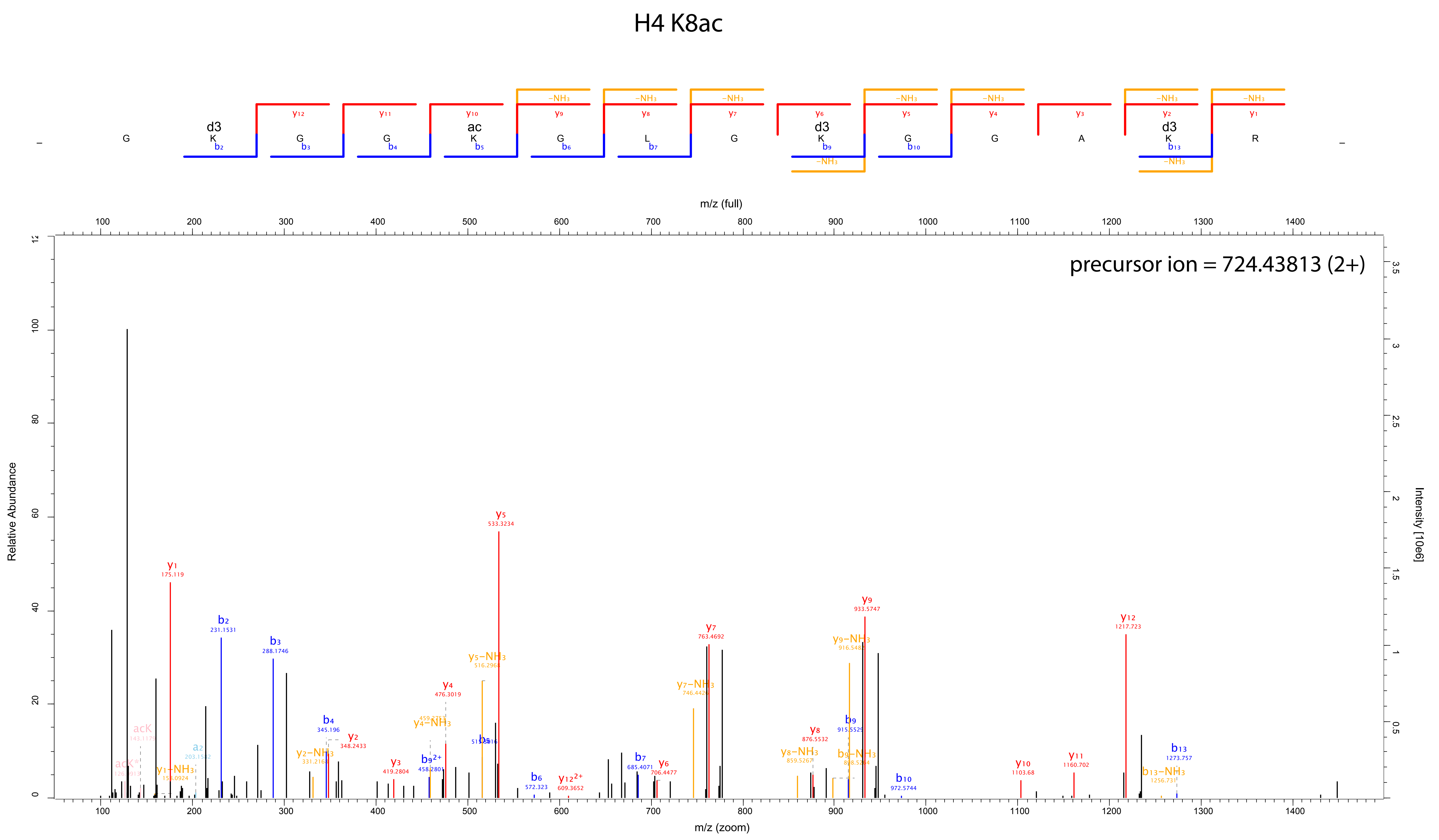
H4 K8ac
precursor ion = 724.43813 (2+)
m/z (full)
m/z (zoom)
Relative Abundance
Intensity [10e6]
y5
533.3234
y1
175.119
b2
231.1531
b3
288.1746
y9
933.5747
y7
763.4692
y12
1217.723
y4
476.3019
b4
345.196
y2
348.2433
y3
419.2804
b6
572.323
b7
685.4071
y6
706.4477
y8
876.5532
b9
915.5529
b10
972.5744
y10
1103.68
y11
1160.702
b13
1273.757

H4 K8acK16ac

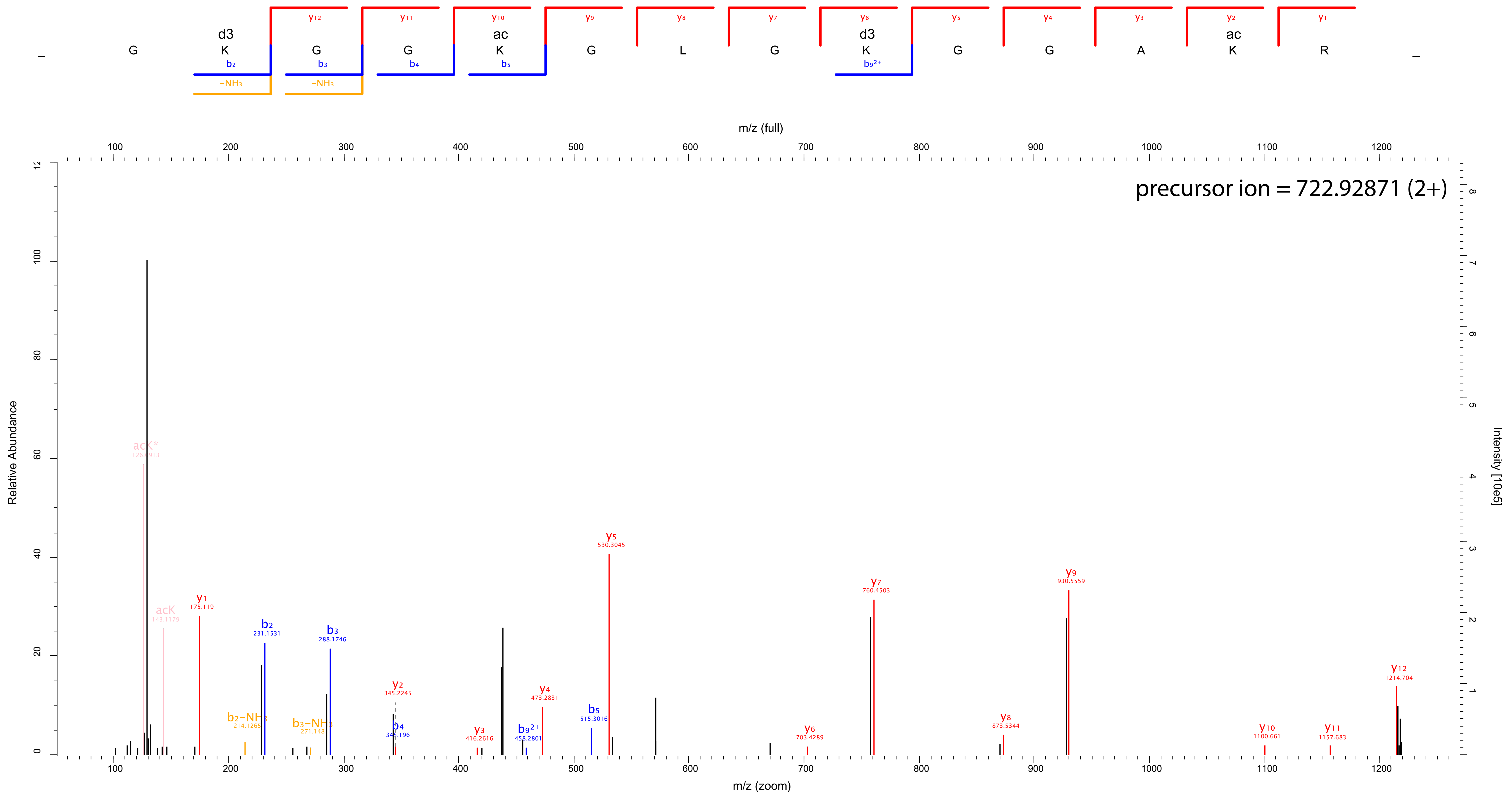

H4 K12acK16ac

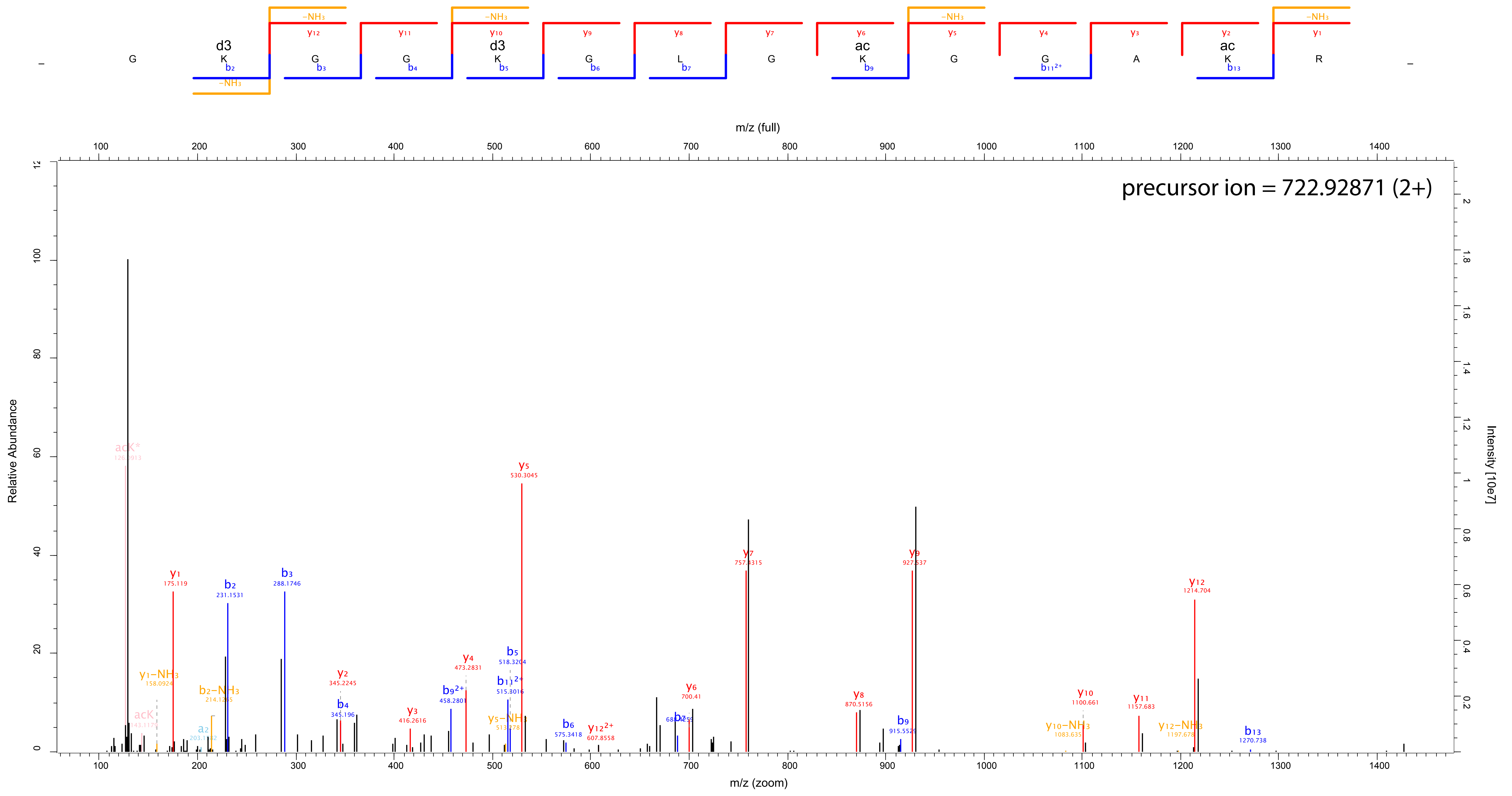

H4 K5acK12acK16ac

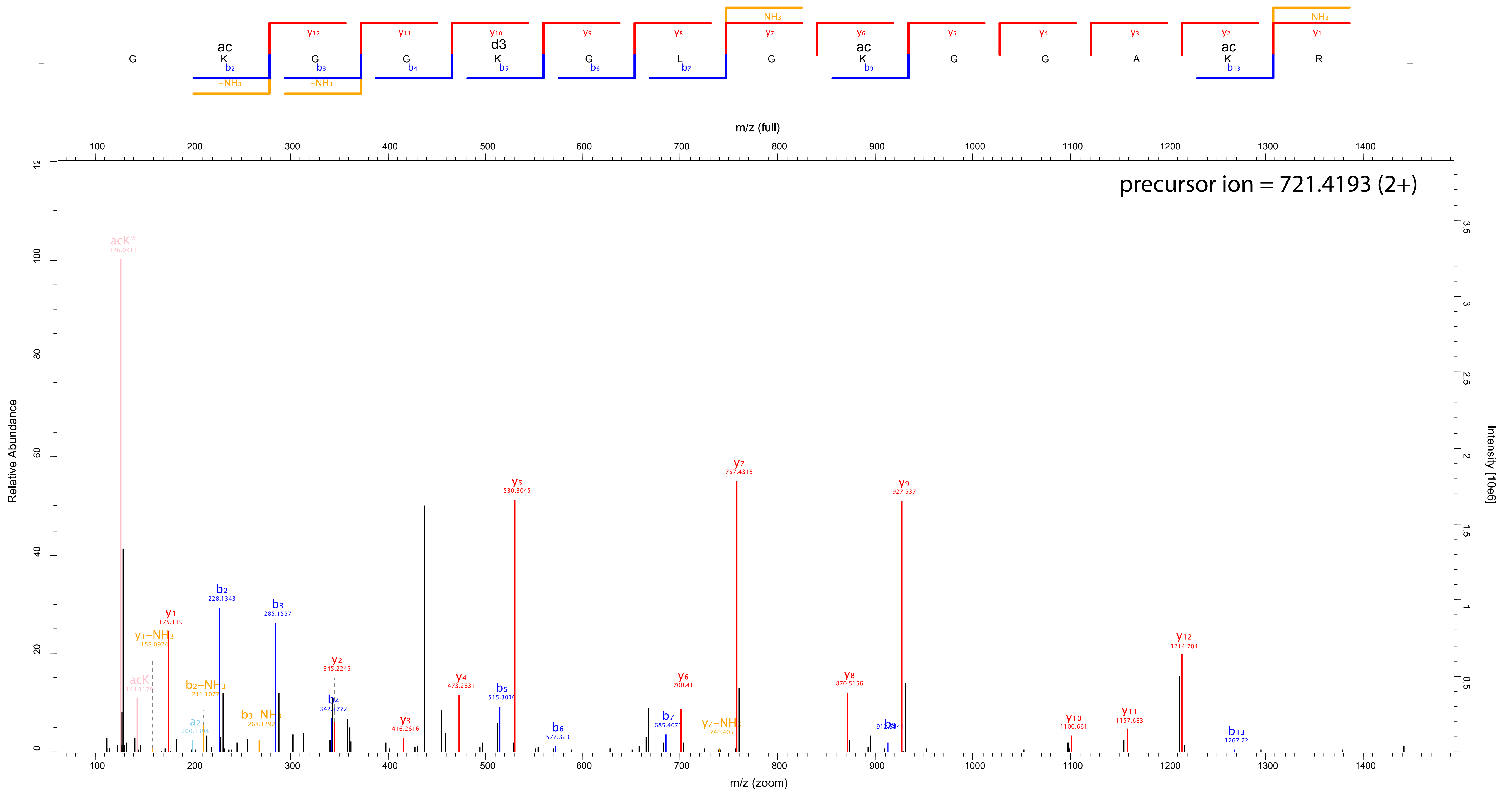

H4 K5me1

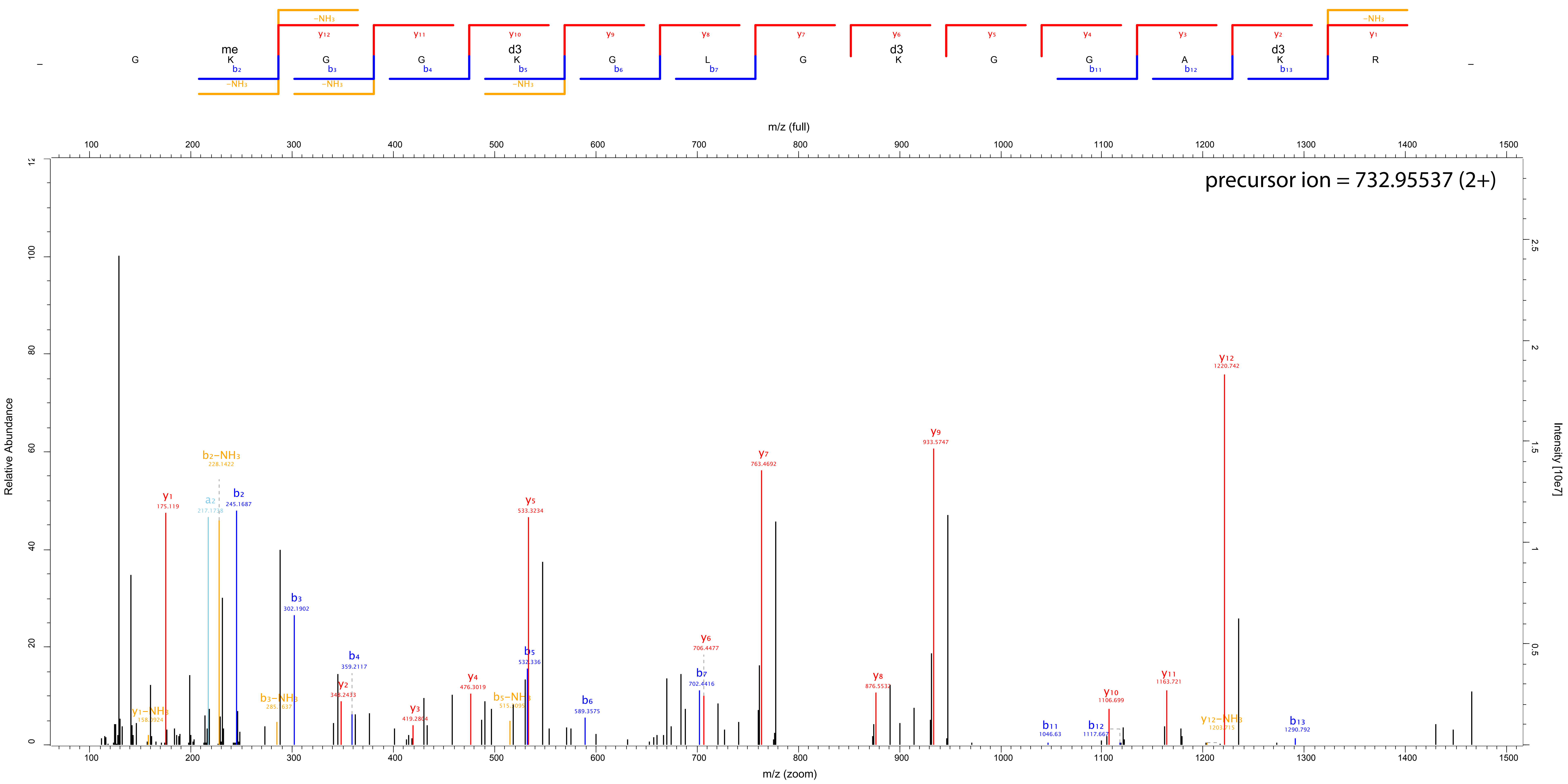
precursor ion = 732.95537 (2+)
m/z (full)
m/z (zoom)
Relative Abundance
Intensity [10e7]

H4 K8me1

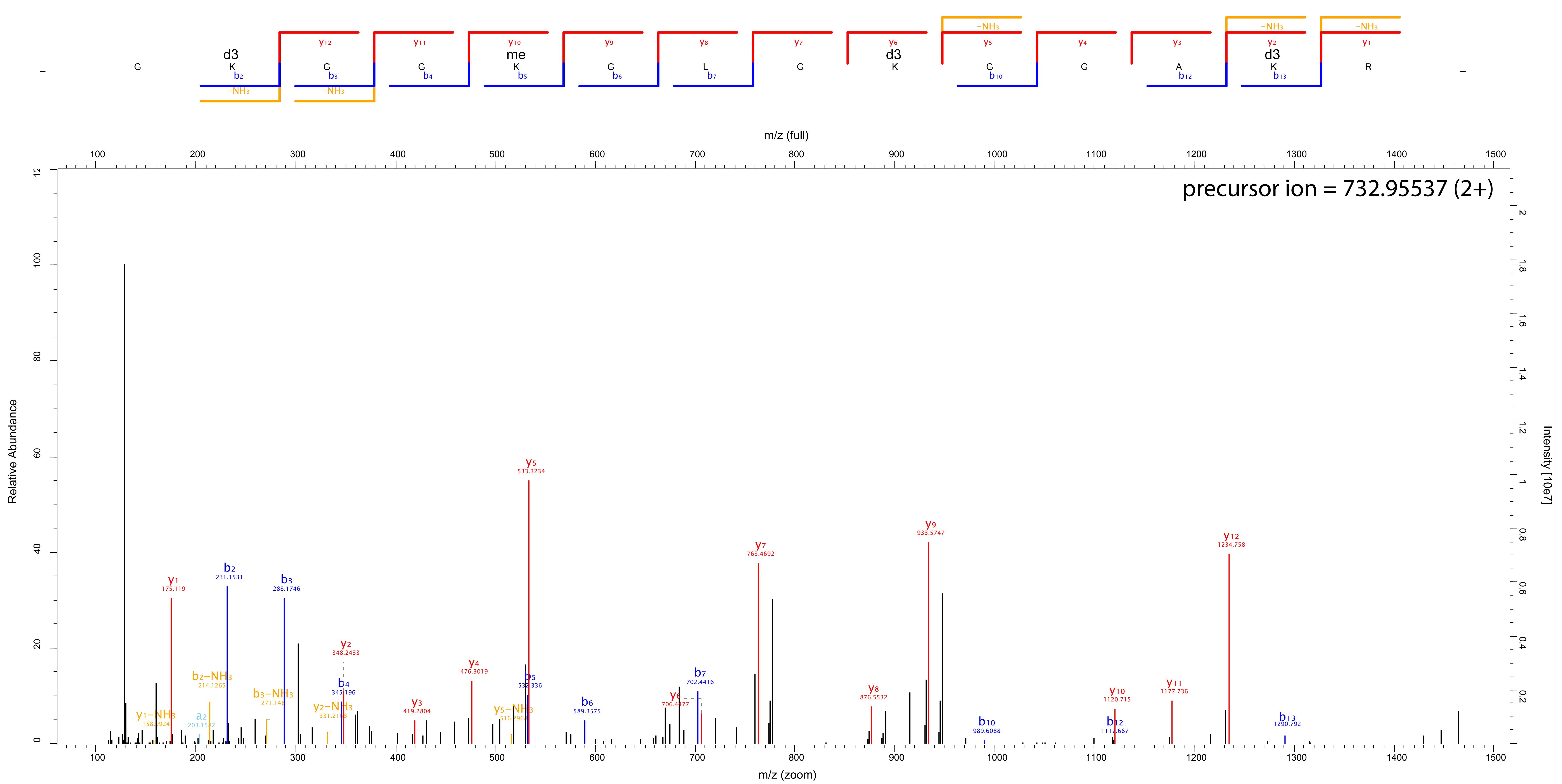

precursor ion = 732.95537 (2+)
m/z (full)
m/z (zoom)
Relative Abundance
Intensity [10e7]
G K G G K G L G K G G A K R
d3
me
d3
d3
y1 175.119
b2 231.1531
b3 288.1746
y2 348.2433
b4 345.1196
y3 419.2804
y4 476.3019
y5 533.3234
b5 532.336
b6 589.3575
y6 706.4877
b7 702.4416
y7 763.4692
y8 876.5532
y9 933.5747
b10 989.6088
y10 1120.715
b12 1117.667
y11 1177.736
y12 1234.758
b13 1290.792
b2−NH3 214.1265
b3−NH3 271.148
y1−NH3 158.0924
a2 203.1582
y2−NH3 331.2168
y5−NH3 516.2969

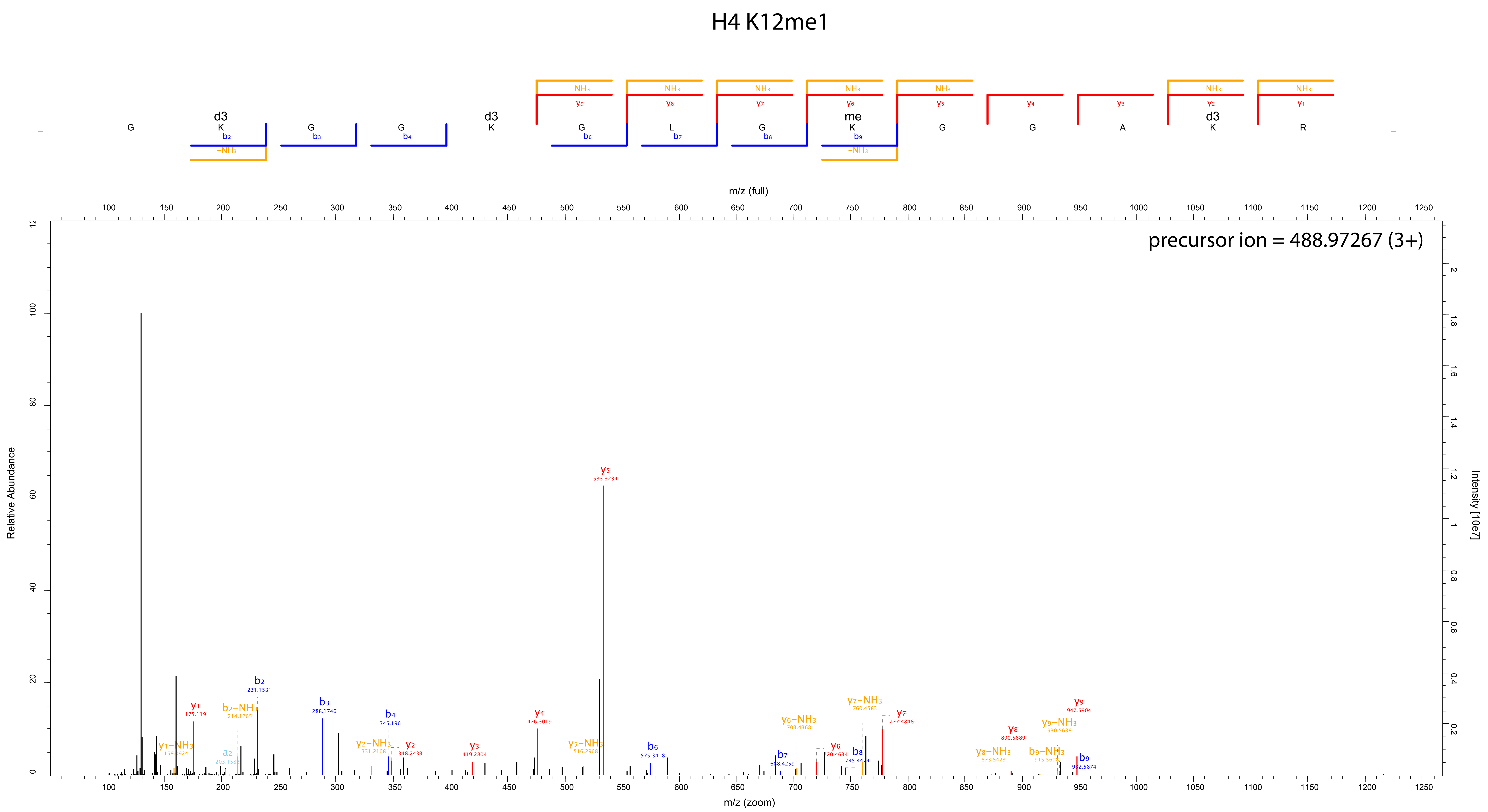

H4 K12me1
precursor ion = 488.97267 (3+)
m/z (full)
m/z (zoom)
Relative Abundance
Intensity [10e7]
y5 533.3234
y4 476.3019
b2 231.1531
b3 288.1746
b4 345.196
y1 175.119
y2 348.2433
y3 419.2804
y7 777.4848
y8 890.5689
y9 947.5904
b9 932.5874

H4 K5me1

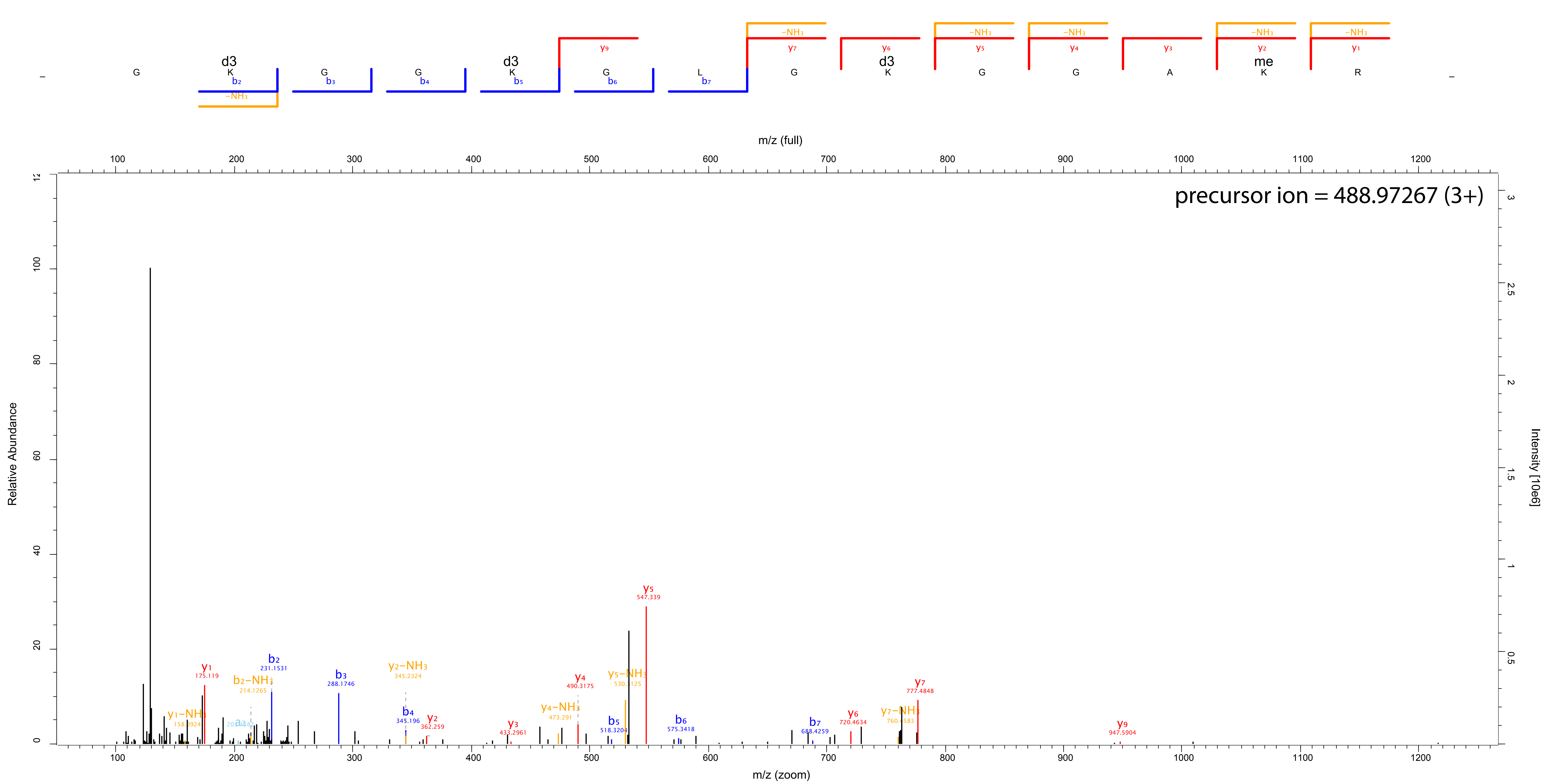

G d3 K G G d3 K G L G d3 K G G A me K R
b2 b3 b4 b5 b6 b7
y9 y7 y6 y5 y4 y3 y2 y1
-NH3
m/z (full)
precursor ion = 488.97267 (3+)
Relative Abundance
Intensity [10e6]
y1 175.119
y1-NH3 158.0924
b2-NH3 214.1265
b2 231.1531
b3 288.1746
y2-NH3 345.2324
b4 345.196
y2 362.259
y3 433.2961
y4-NH3 473.291
y4 490.3175
b5 518.3204
y5-NH3 530.3125
y5 547.339
b6 575.3418
b7 688.4259
y6 720.4634
y7-NH3 760.4583
y7 777.4848
y9 947.5904
m/z (zoom)

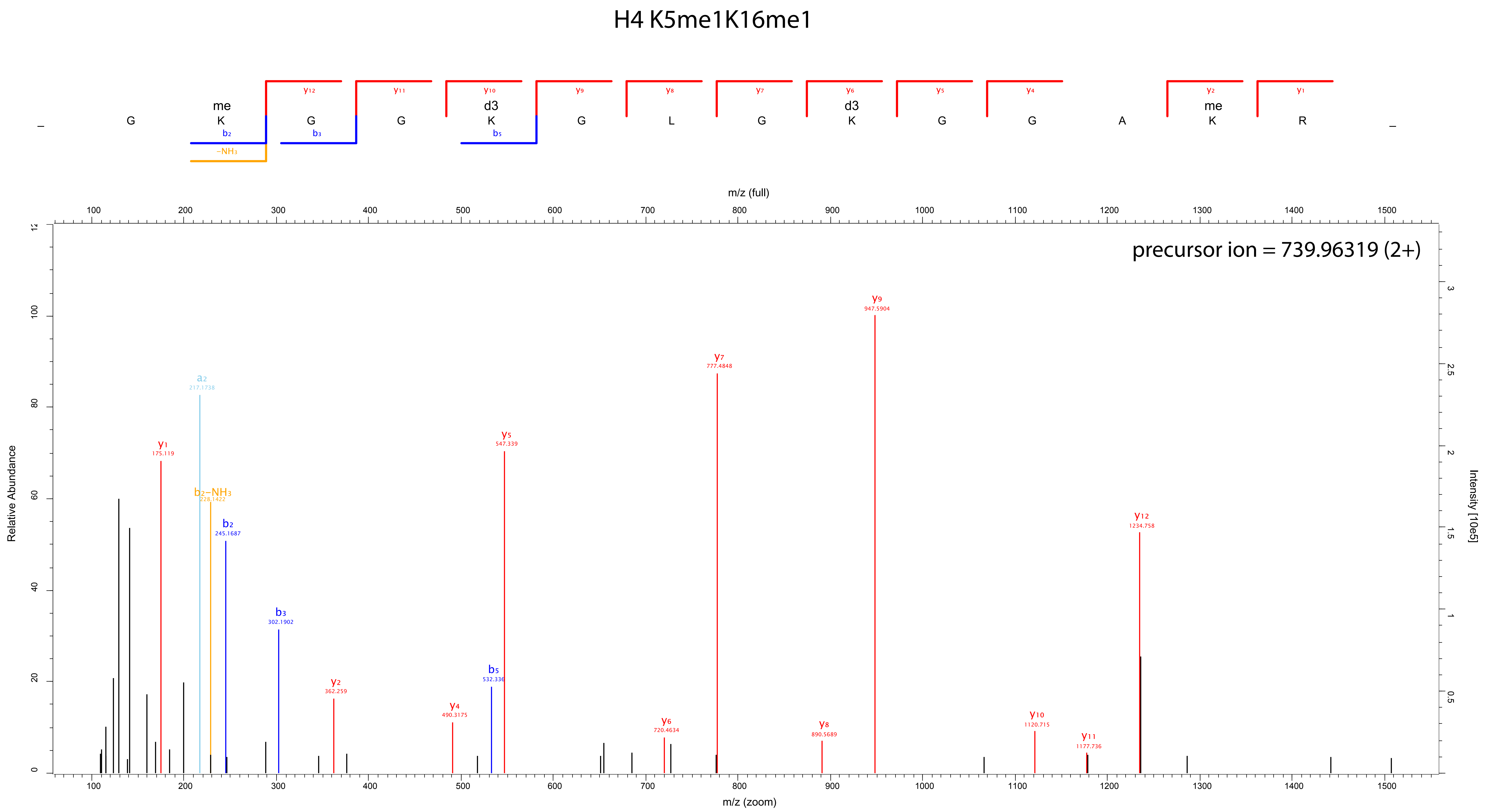
H4 K5me1K16me1
precursor ion = 739.96319 (2+)
m/z (full)
m/z (zoom)
Relative Abundance
Intensity [10e5]
y1 175.119
a2 217.1738
b2-NH3 228.1422
b2 245.1687
b3 302.1902
y2 362.259
y4 490.3175
b5 532.336
y5 547.339
y6 720.4634
y7 777.4848
y8 890.5689
y9 947.5904
y10 1120.715
y11 1177.736
y12 1234.758

H4 K8me1K12me1

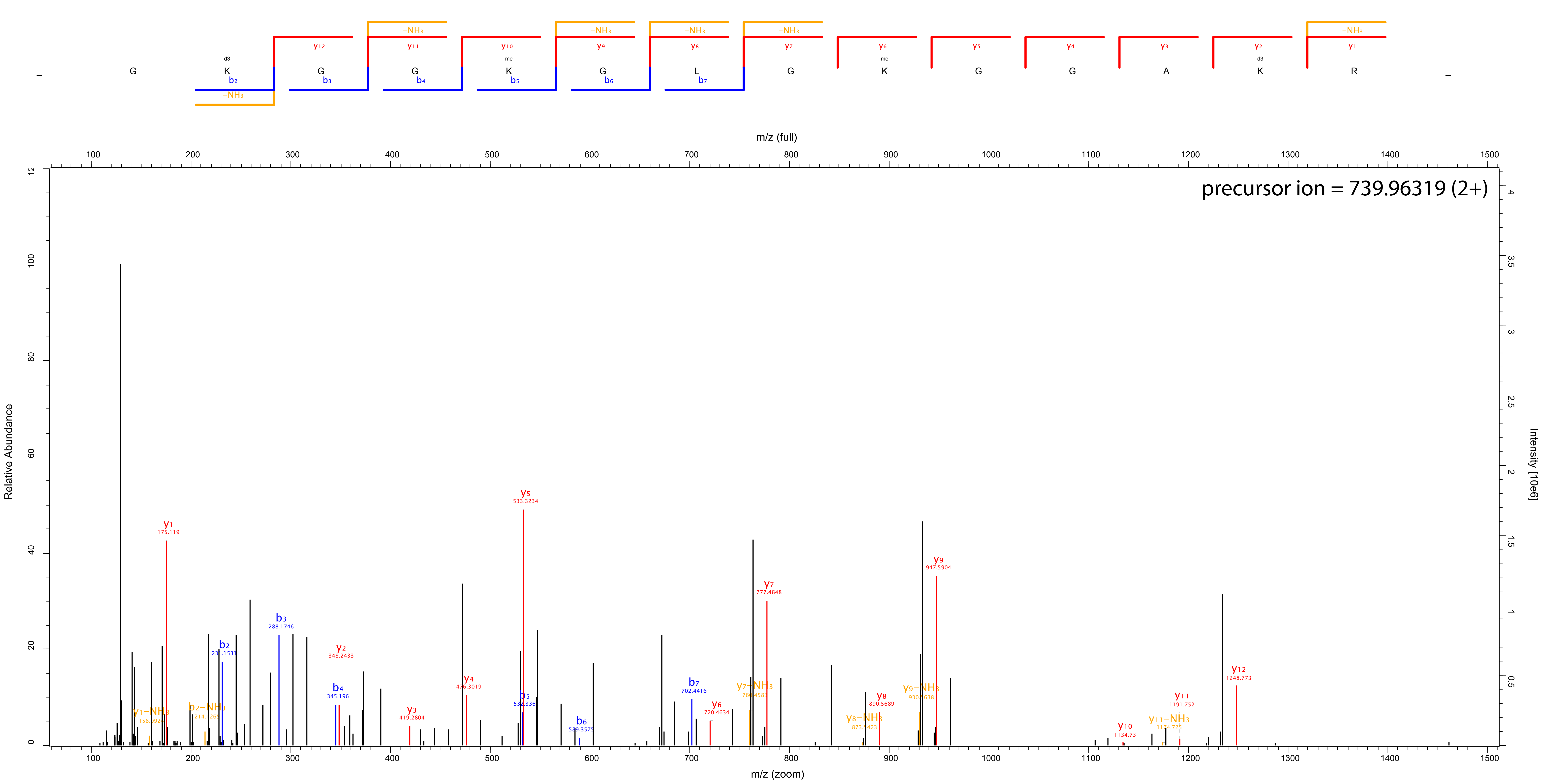

H4 K5me1K15ac

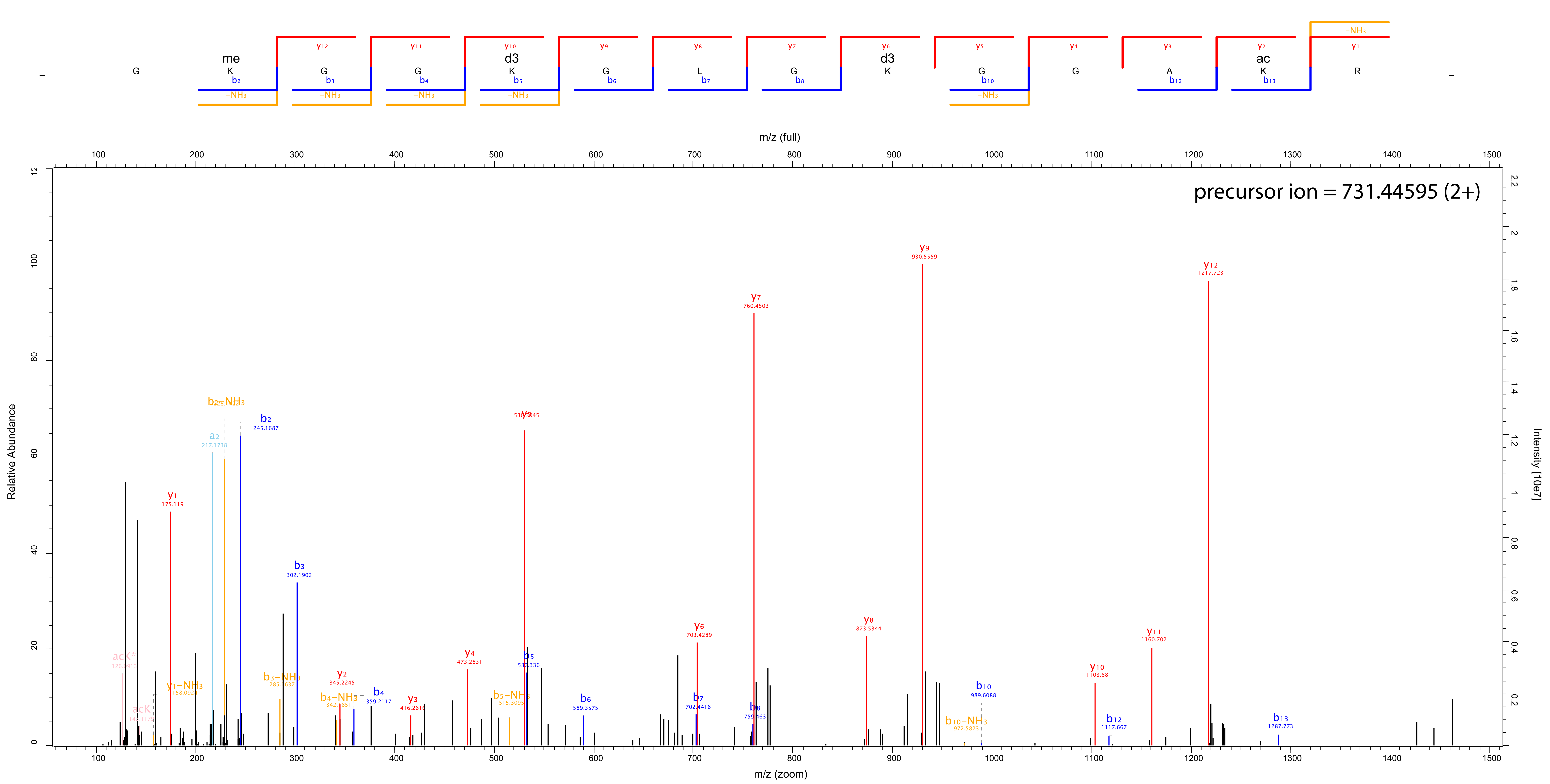

H4 K8me1K16ac

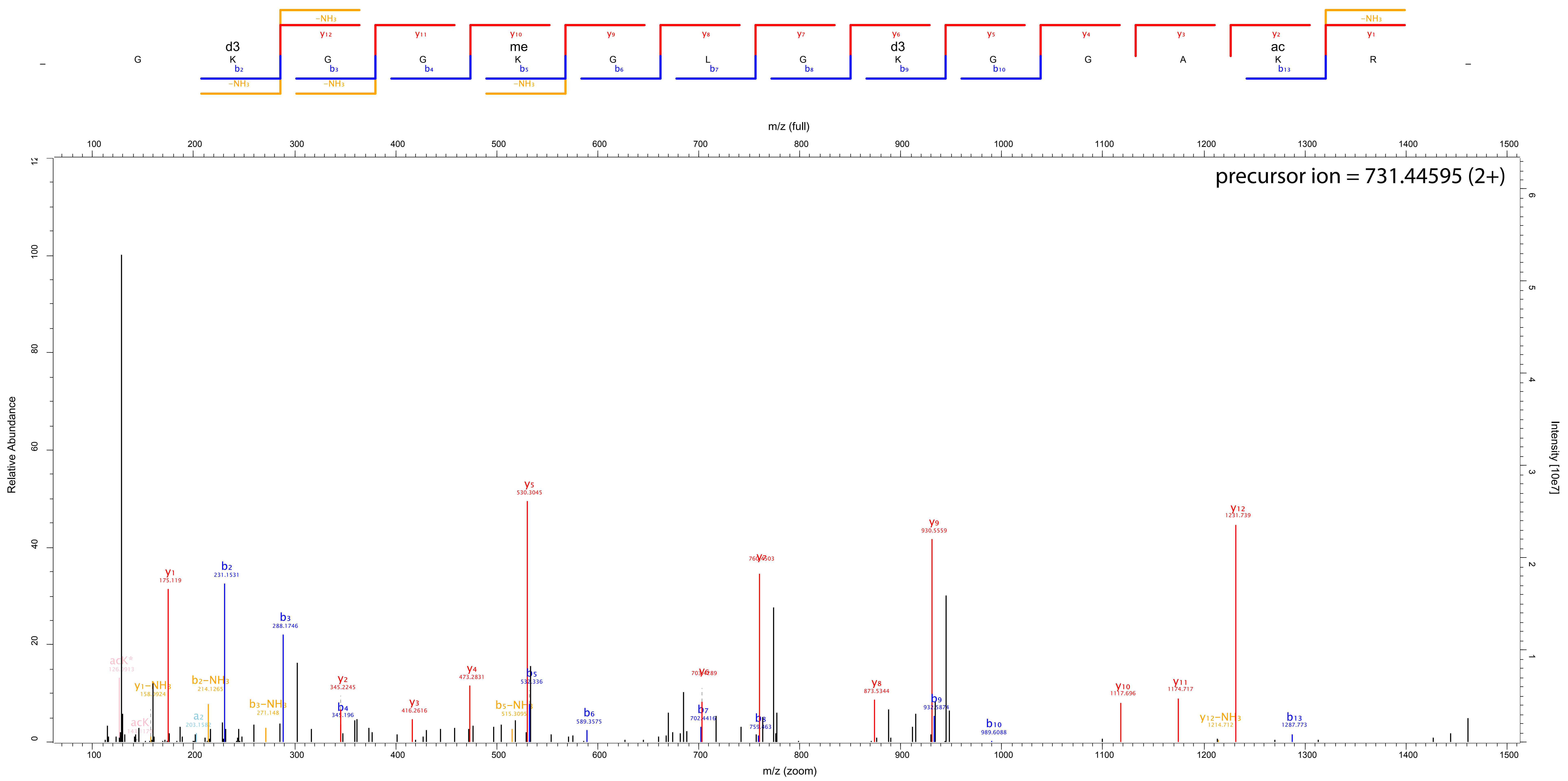

H4 K12acK16me1

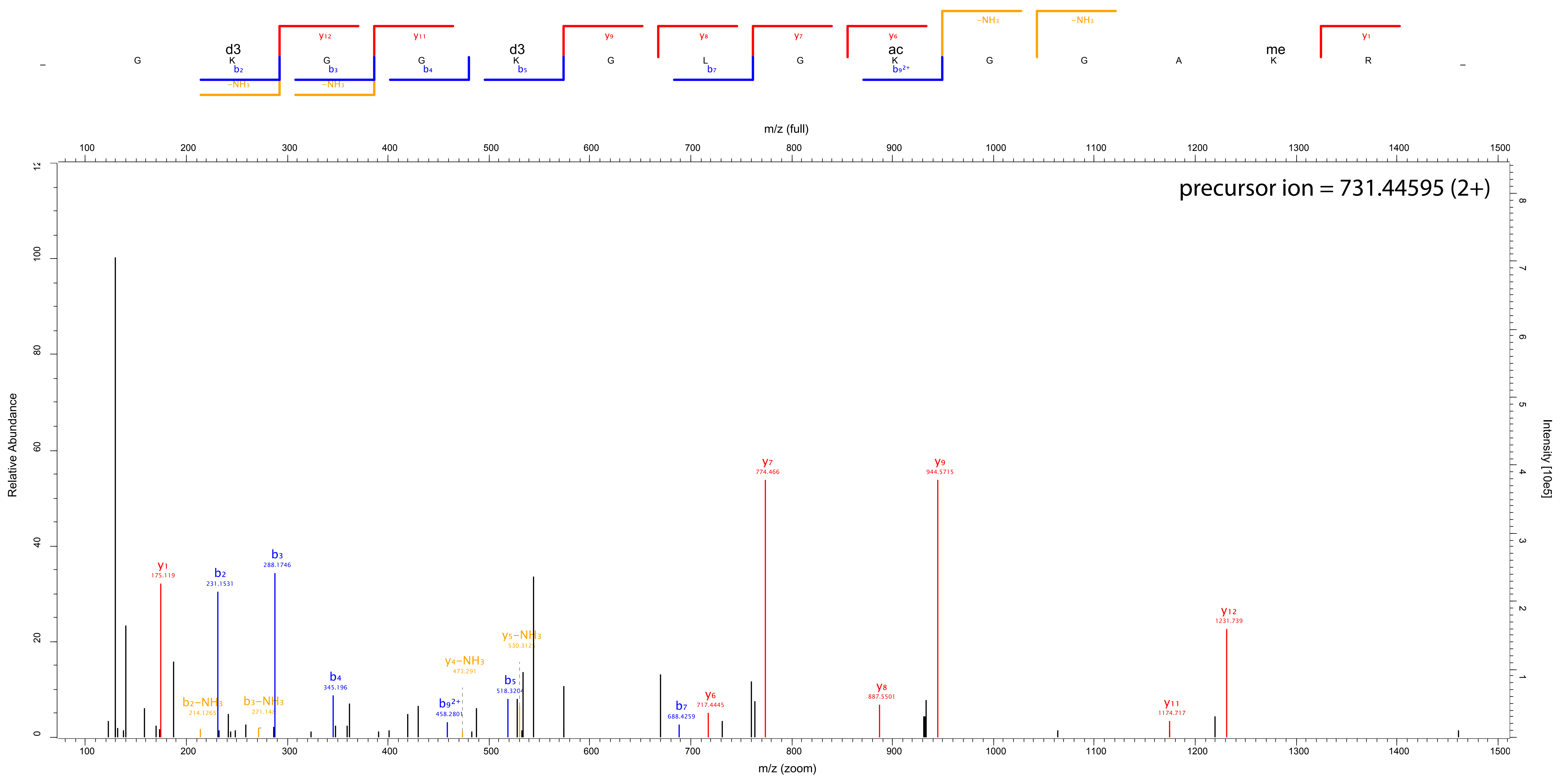

H4 K5form

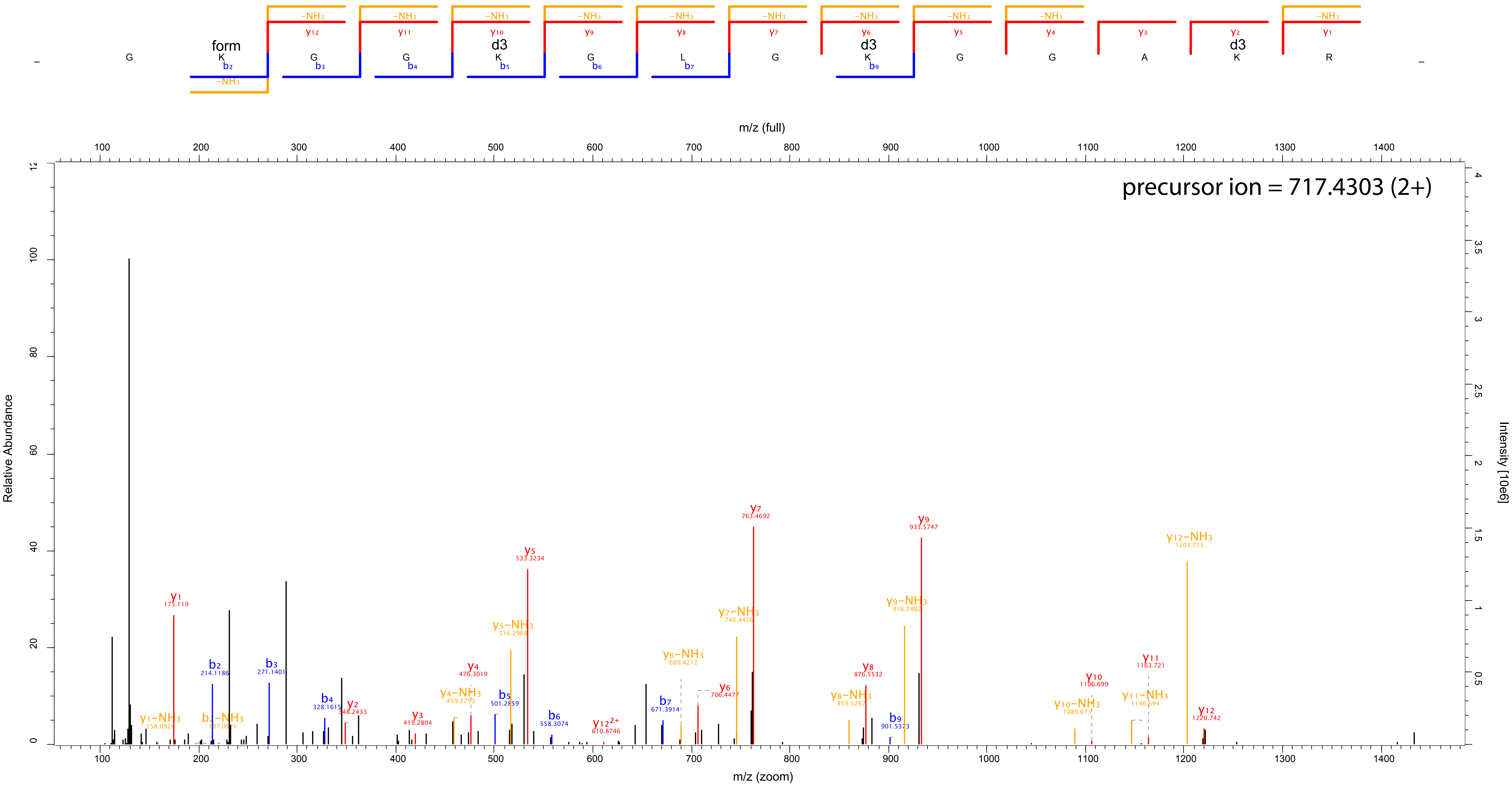

G
form
K
G
G
d3
K
G
L
G
d3
K
G
G
A
d3
K
R
m/z (full)
precursor ion = 717.4303 (2+)
Relative Abundance
Intensity [10e6]
m/z (zoom)
y1 175.119
y1−NH3 158.0924
b2 214.1186
b3 271.1401
b4 328.1615
y2 348.2433
y3 419.2804
y4 476.3019
y4−NH3 459.2753
b5 501.2859
y5 533.3234
y5−NH3 516.2968
b6 558.3074
y12 2+ 610.8746
b7 671.3914
y6−NH3 689.4212
y6 706.4477
y7 763.4692
y7−NH3 746.4426
y8 876.5532
y8−NH3 859.5267
b9 901.5373
y9 933.5747
y9−NH3 916.5482
y10 1106.699
y10−NH3 1089.673
y11 1163.721
y11−NH3 1146.694
y12−NH3 1203.715
y12 1220.742

H4 K8form

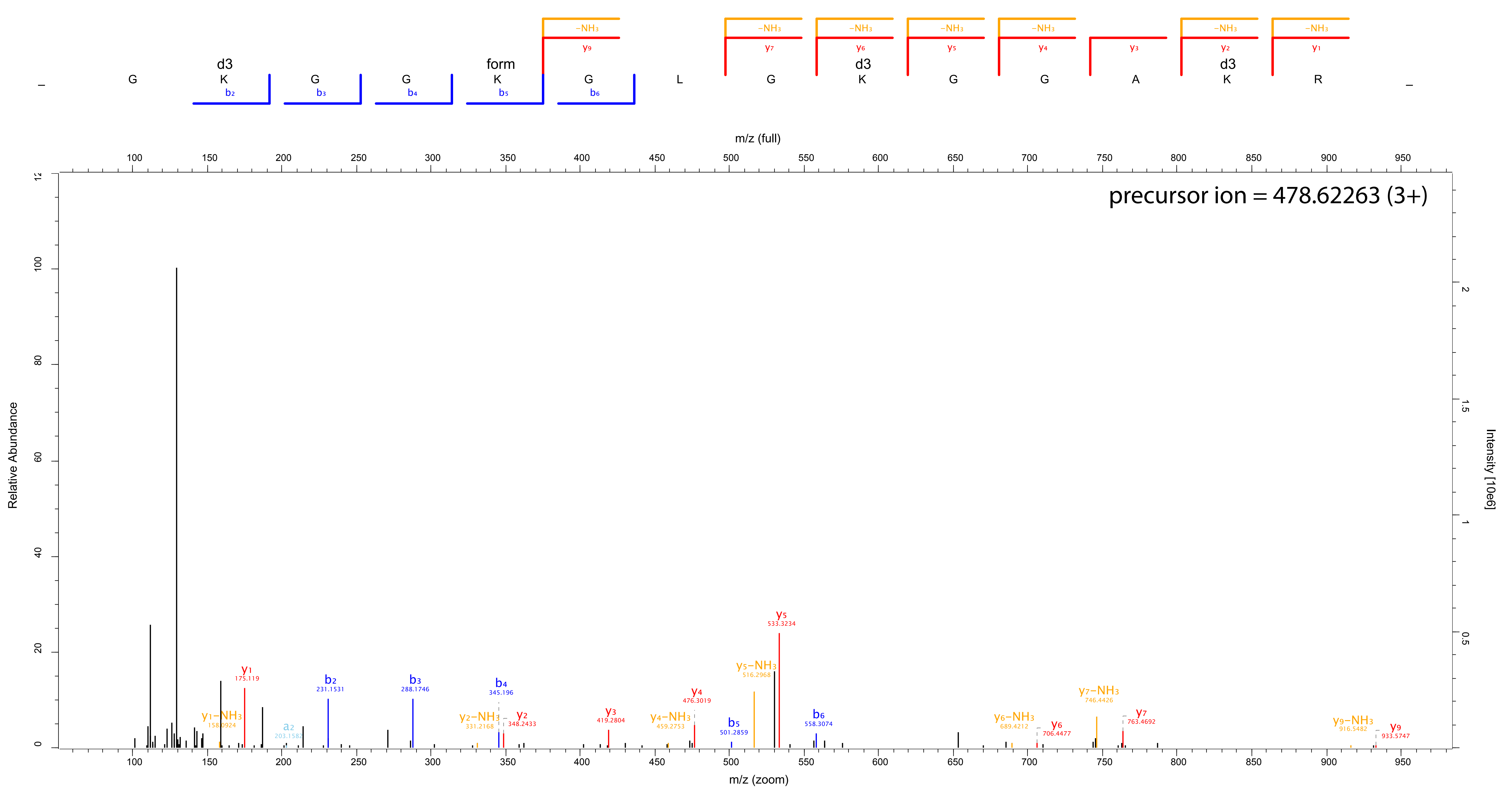

precursor ion = 478.62263 (3+)
G d3K G G formK G L G d3K G G A d3K R
b2 b3 b4 b5 b6
y9 y7 y6 y5 y4 y3 y2 y1
-NH3
m/z (full)
m/z (zoom)
Relative Abundance
Intensity [10e6]
y1 175.119
y1-NH3 158.0924
a2 203.1582
b2 231.1531
b3 288.1746
b4 345.196
y2-NH3 331.2168
y2 348.2433
y3 419.2804
y4 476.3019
y4-NH3 459.2753
b5 501.2859
y5-NH3 516.2968
y5 533.3234
b6 558.3074
y6-NH3 689.4212
y6 706.4477
y7-NH3 746.4426
y7 763.4692
y9-NH3 916.5482
y9 933.5747

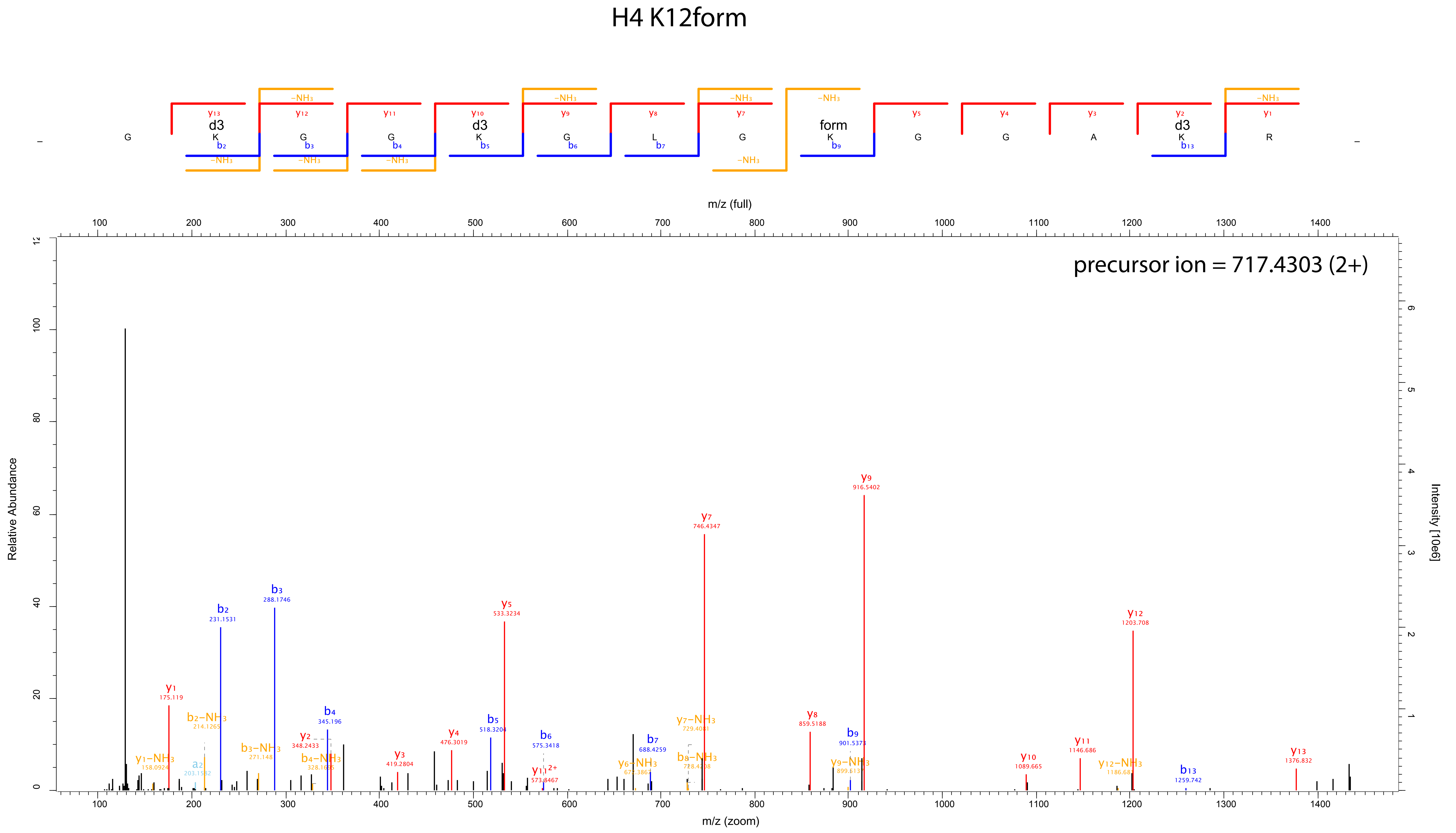
H4 K12form
precursor ion = 717.4303 (2+)
m/z (full)
m/z (zoom)
Relative Abundance
Intensity [10e6]
y9
916.5402
y7
746.4347
y12
1203.708
b3
288.1746
b2
231.1531
y5
533.3234
y1
175.119
b4
345.196
y8
859.5188
b5
518.3204
b9
901.5373
y4
476.3019
b6
575.3418
b7
688.4259
y3
419.2804
y10
1089.665
y11
1146.686
y13
1376.832
b13
1259.742
y2
348.2433
y7−NH3
729.4081
b2−NH3
214.1265
b3−NH3
271.148
y1−NH3
158.0924
b4−NH3
328.1695
y12−NH3
1186.68
y9−NH3
899.6137
b8−NH3
728.4208
y6−NH3
672.3867
a2
203.1532
y11 2+
573.8467
